# Water-Stable Upconverting Coordination Polymer Nanoparticles for Transparent Films and Anticounterfeiting Patterns with Air-Stable Upconversion

**DOI:** 10.1021/acsami.2c16354

**Published:** 2023-02-01

**Authors:** Junda Zhang, Daniel Ruiz-Molina, Fernando Novio, Claudio Roscini

**Affiliations:** † Catalan Institute of Nanoscience and Nanotechnology (ICN2), CSIC and BIST Campus UAB, Bellaterra 08193, Barcelona, Spain; ‡ Departament de Química, Universitat Autònoma de Barcelona (UAB), Campus UAB, 08193 Cerdanyola del Vallès, Spain

**Keywords:** triplet−triplet annihilation, upconversion, coordination polymer nanoparticles, metal−organic framework, luminescent solar concentrators

## Abstract

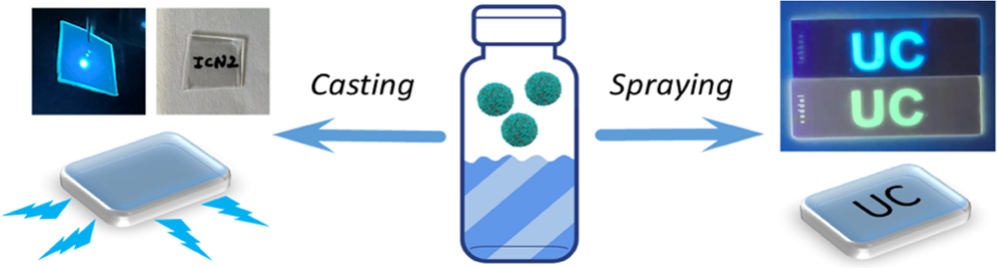

Photon upconversion (UC) based on triplet–triplet annihilation is a very promising phenomenon with potential application in several areas, though, due to the intrinsic mechanism, the achievement of diffusion-limited solid materials with air-stable UC is still a challenge. Herein, we report UC coordination polymer nanoparticles (CPNs) combining sensitizer and emitter molecules especially designed with alkyl spacers that promote the amorphous character. Beyond the characteristic constraints of crystalline MOFs, amorphous CPNs facilitate high dye density and flexible ratio tunability. To show the universality of the approach, two types of UC-CPNs are reported, exhibiting highly photostable UC in two different visible spectral regions. Given their nanoscale, narrow size distribution, and good chemical/colloidal stability in water, the CPNs were also successfully printed as anticounterfeiting patterns and used to make highly transparent and photostable films for luminescent solar concentrators, both showing air-stable UC.

## 1 Introduction

Photon energy upconversion (UC) is a process in which a high-energy emission is generated through the absorption of two or more lower-energy photons.^1−3^ So, materials exhibiting this feature have raised much attention for their potential applicability in several fields such as bioimaging,^4,5^ biotherapy,^6^ solar cells and energy storage,^7,8^ photocatalysis (to remove air and/or water contaminants upon solar light irradiation),^9^ photopolymerization,^10^ optoelectronic devices (e.g., luminescent solar concentrators, OLEDs),^11−13^ and anticounterfeiting.^14^ To date, most of the reported UC materials have been represented by rare-earth nanoparticles and sensitizer–emitter molecular organic dye pairs undergoing UC through triplet–triplet annihilation (TTA-UC).^15^ The former group relates to inorganic bulk and nanostructured materials,^1,16,17^ mainly made by lanthanide ions embedded in an inorganic crystalline host lattice, though typical drawbacks are the very narrow ion absorption bands, low extinction coefficients, high radiation power densities required (∼W cm^–2^), and relatively low UC quantum yields, besides low biocompatibility and toxicity. In contrast, organic-based TTA-UC benefits from the broad absorption of the sensitizers and the high fluorescence quantum yield of emitter organic molecules. Generally, sensitizer molecules are at first excited upon absorption of a photon forming the singlet excited state. After generating a triplet excited state (via intersystem-crossing), the sensitizer molecules undergo triplet–triplet energy transfer (TTET) to the energy acceptor (or emitter), yielding emitter units excited in their triplet state. Once enough excited molecules of the acceptor are produced, the triplet–triplet annihilation (TTA) process occurs forming both ground-state and highly excited emitter molecules, which release their energy by emitting photons from the singlet excited state (S1), i.e., of higher energy than that used to excite the photosensitizer (see Scheme S1 in the Supporting Information).^1^

While inorganic UC systems are normally obtained as solid bulk materials or nanoparticles,^18,19^ a still ongoing research study is challenging the achievement of air-stable molecular-based UC solid materials for practical applications.^20,21^ The main challenges derive from (i) quenching of the involved excited triplet states by the oxygen in the air atmosphere, which reduces the UC efficiency^22−24^ and (ii) the need for dye pairs to diffuse and approach within 1 nm for the bimolecular processes involved in UC (TTET and TTA) to efficiently take place via the Dexter energy transfer mechanism.^25,26^ Moreover, it is often desired to have these materials as colloidal and chemically stable nanoparticles in aqueous media, for their biological and environmentally friendly applications, for instance as bioimaging markers or water-based ink formulations, and/or redispersed in polymeric matrices as transparent emitting devices (OLEDs, luminescent solar concentrators).^12,13,27,28^

During the years, several strategies have been developed to accomplish such TTA-UC bulk and nanostructured solids: direct dye dissolution in rubber-like low-*T*_g_ polymers (e.g., polyurethane),^29,30^ functionalization of the glassy polymer backbone (e.g., polymethyl methacrylate) or epoxy resins with the dye pairs,^31,32^ gels (hydro- and organogels),^33−35^ ionic liquid-containing or highly dye-concentrated epoxy resins,^36^ polymer or silica liquid-core micro- and nanocapsules,^4,22,37,38^ and liquid nanodroplets confined in polymer materials. More recently, TTA-UC was extended to highly limited diffusion materials, with efficient triplet self-exchange or triplet energy migration between highly concentrated and/or aligned chromophores. In this respect, we reported the TTA-UC in solid-state paraffin waxes (with nearly quantitative TTET), where the aromatic dye molecules (sensitizers and emitters) were forced to coprecipitate and aggregate in the solid-state aliphatic paraffin, due to their limited solubility.^3^ A similar approach relies on the formation of binary crystals, where dense dye packing guarantees efficient triplet migration and good UC efficiency.^14,39^ However, even if several approaches have been developed, the synthesis of solid TTA-UC materials still represents a challenge.

With this aim, recently, coordination chemistry has emerged as a promising alternative. In addition to their rich chemistry and a broad range of useful ligands, metal coordination minimizes molecular migration and phase segregation normally observed in dye-loaded nanoparticles or polymer matrices. In this context, metal–organic frameworks (UC-MOFs) emerge as excellent candidates promoting high density and suitable alignment of the emitter molecules within the framework, with efficient triplet migration and TTA properties.^5,40−44^ These materials take advantage of their open-framework structure to physically trap the sensitizer within the pores, or sometimes even on the surface, to promote TTA-UC, though this configuration might verify leaching issues if the interaction with the metal center is not too strong, preventing control over two important features for the TTET efficiency, which are the sensitizer-to-emitter ratio and/or the sensitizer/emitter distance, especially when these particles are dispersed in other media. The only exception is given by recent work by I. Roy et al.,^43^ which reports for the first time a MOF with both the sensitizer and the emitter being part of the chemical structure of the MOFs. However, due to the inherent structural properties of the MOFs, the local emitter/sensitizer ratio is fixed and depends on the coordination properties of the metal ion. Efficient TTET is normally achieved with a large excess of the emitter; therefore, the local emitter/sensitizer ratio is an important parameter that cannot be controlled above a certain limit with MOF structures. On top of that, most of the reported materials are based on microstructured MOFs, unsuitable for certain applications where colloidal stability and optical transparency are required.

**Figure 1:**
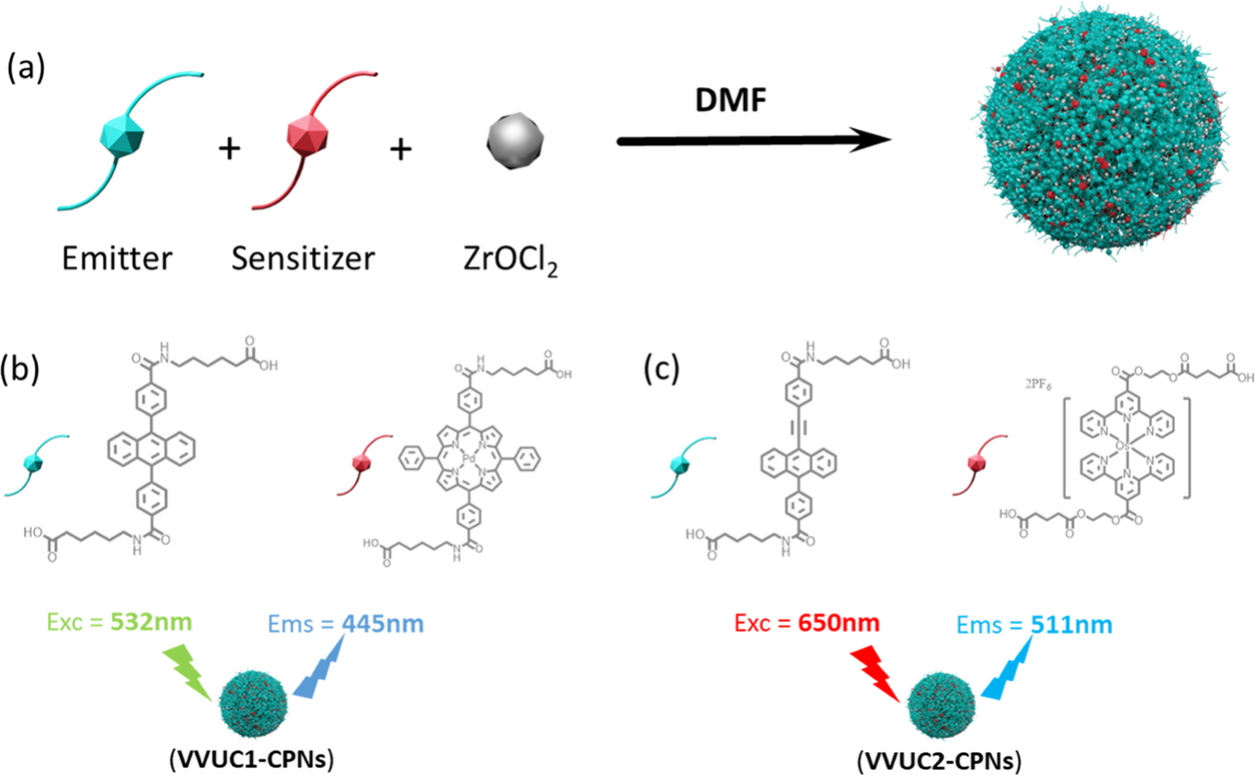
(a) Scheme of the synthesis of UC-CPNs using an emitter and a sensitizer chemically modified with a spacer as monomers, and Zr^2+^ as a cross-linker. (b, c) Chemical structures of the sensitizers (**Pd-S-COOH** and **Os-S-COOH**) and emitters (**DPA-S-COOH** and **CAEBD-S-COOH**) functionalized with COOH-terminated spacers and used to prepare **VVUC1-CPNs** and **VVUC2-CPNs**.

Herein, we hypothesize that these challenges can be successfully faced with the use of amorphous coordination polymer nanoparticles (CPNs). These nanostructures, in addition to hosting both sensitizer and emitter molecules as constitutive building blocks of the polymeric network, guarantee dye density and flexible ratio tunability, i.e., a broad control of the number of emitter molecules surrounding the sensitizer, normally required to achieve UC (Figure 1a). Moreover, these nanoparticles have already been shown to exhibit good chemical and colloidal stability in water,^45,46^ which opens new venues for the application of these nanostructures in biology and other environmentally friendly applications.

## 2 Results and Discussion

### 2.1 Upconverting Coordination Polymer Nanoparticles

To demonstrate the viability of our approach, we designed two pairs of dyes, as shown in Figure 1b,c: the introduction of the alkyl chain contributes the necessary flexibility to have amorphous systems without significantly diluting the UC active dye weight mass in the nanoparticles. This is very relevant to ensure the high dye density required for an efficient energy transfer and triplet migration to take place, even when the particles are embedded in other solid media (e.g., polymers). The chemical design of the ligands leads to obtaining monodisperse and water-stable (chemically and colloidal) nanoparticles that exhibit photostable UC in different visible spectral regions, upon irradiation at λ_exc_ = 532 nm (**VVUC1-CPNs**) and λ_exc_ = 650 nm (**VVUC2-CPNs**), respectively.

**Figure 2:**
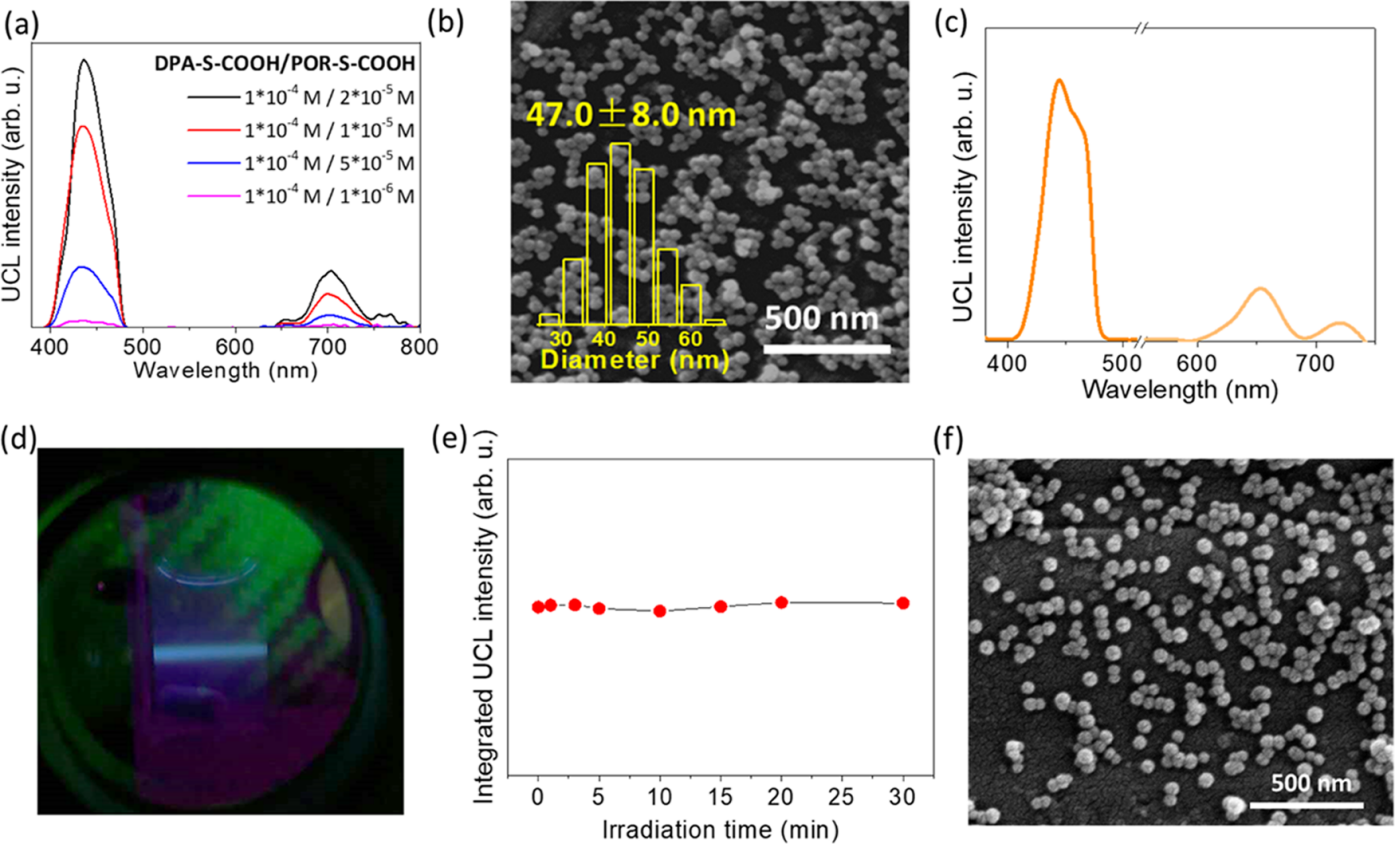
(a) Emission spectra of DMF solutions of **DPA-S-COOH/Pd-S-COOH** at different molar ratios (λ_exc_ = 532 nm, N_2_ atmosphere). (b) SEM image, (c) emission spectrum, and (d) photograph of the emission of the aqueous suspension of **VVUC1-CPNs** obtained from **DPA-S-COOH/Pd-S-COOH** = 1281 (Table S1, entry 3; λ_exc_ = 532 nm, N_2_ atmosphere). A magenta subtractive dichroic color filter was used to cut the 532 nm excitation beam. (e) Integrated UC intensities of **VVUC1-CPNs** (1.0 mg/mL) upon prolonged irradiation (up to 30 min) with a CW 532 nm laser (1.2 W/cm^2^). (f) SEM image of **VVUC1-CPNs** after 30 irradiation with a CW 532 nm laser (1.2 W/cm^2^).

First, we synthesized chemically functionalized palladium(II) meso-tetraphenylporphyrin (**Pd-S-COOH**) and 9,10-diphenylanthracene (**DPA-S-COOH**) ligands, whose parent molecules are already well-known dye pairs (sensitizer and emitter) that manifest visible-to-visible UC (see the SI). They were synthesized with 6 (1.8% overall yield) and 4 (41.2%) steps, respectively, from commercial starting materials. The dyes core units were functionalized with two 5-carbon carboxylic acid-terminated chains, the non-rigid spacers (S), through the formation of an amide bond (Figure 1b). The detailed synthetic routes and chemical characterization of the compounds are described in the SI (Sections S2 and S3). The absorption and emission spectra of **DPA-S-COOH** and **Pd-S-COOH** DMF solutions resembled those reported for the parent DPA (λ_max_^abs^ = 375 nm; λ_max_^em^ = 433 nm, Figure S1a,b in Section 6 in the SI) and PdTPP,^47^ demonstrating that the functionalization did not significantly affect the optical properties and the electronic state energies of the dyes. The emission quantum yield (QY) of **DPA-S-COOH** was 98.9%, similar to what were reported for the parent dye in different solvents.^48^ The DMF solution of **Pd-S-COOH** showed Q-band absorption and phosphorescence (under a N_2_ atmosphere) maxima at λ_max_ = 523 and 700 nm, respectively, and a phosphorescence lifetime of 503 μs (Figure S1c,d). The maximum phosphorescence at 700 nm recorded at room temperature allowed us to estimate a triplet state energy of **Pd-S-COOH** of 1.77 eV. Noticeably, degassed *N*,*N-*dimethylformamide (DMF) solutions of these novel dyes dissolved in different molar ratios did show successful anti-Stokes emission (λ_max_ = 435 nm, 2.85 eV) when irradiated with a 532 nm (2.33 eV) laser (i.e., anti-Stokes shift, Δ*E*_UC_ = 0.52 eV), indicating that the pair is suitable for UC (Figure 2a). From the 0–0 vibronic band of the absorption spectrum of the **DPA-S-COOH** acceptor (λ_0–0_ = 396 nm), the energy of the first singlet excited state was estimated as high as 3.13 eV, quite similar to the reported energy of the parent DPA, determined by TD-DFT and spectral properties.^49,50^ From the thermodynamic driving force of TTA (2 × *E*(T_1_)^**DPA-S-COOH**^ > *E*(S_1_)^**DPA-S-COOH**^) and the sensitizer-to-emitter TTET (*E*(T_1_^**DPA-S-COOH**^) < *E*(T_1_^**Pd-S-COOH**^)), the triplet state energy of the emitter was also determined in the range of 1.56 eV < T_1_^**DPA-S-COOH**^ < 1.77 eV, which lays around the value calculated for the lowest triplet energy of the parent DPA (1.72 eV).^50^ These results showed that the chemical functionalization did not affect significantly the lowest excited-state energies of the emitter.

Visible-to-visible **VVUC1-CPNs** were thus straightforwardly obtained by mixing **Pd-S-COOH** and **DPA-S-COOH**, with different relative initial concentrations, in anhydrous DMF for 5 min at room temperature (Table S1). Afterward, zirconium oxychloride was added leaving the mixture at 90 °C for an additional 15 h. After this period, a precipitate was obtained, centrifuged, and isolated by freeze-drying. Zr^2+^ was chosen as a cross-linking metal ion, as it provides very high robustness to the systems against water^51,52^ but does not provide novel absorption bands, which might affect the optical properties of the CPNs. In all cases, scanning electron microscopy (SEM) corroborated the formation of spherical nanoparticles with an average size of around 50 nm (Figures 2b and S2). High-performance liquid chromatography (HPLC) was used to determine the sensitizer/emitter ratio as well as to quantify the amount of emitter molecules per CPN (see Section 1 in the SI for more details). Inductively coupled plasma-mass spectrometry (ICP-MS) was used to quantify the concentration of sensitizers (through the Pd center) and Zr^2+^ ions. The sensitizer/emitter ratio before and after the reaction for each batch is given in Table S1. Final sensitizer/emitter ratios for the different reactions follow the trend initially used for the reaction, though the quantitative values are twice as high, suggesting the non-equal coordination abilities of the two dyes.

UC emission was investigated in degassed aqueous suspensions, under irradiation with a continuous wave (CW) 532 nm laser. Noticeably, an intense UC emission with λ_max_ = 445 nm was measured for all suspensions, with also a residual phosphorescence contribution at λ_max_ = 653 nm (Figure S3). The integrated UC/phosphorescence ratio changed significantly among all samples, confirming the sensitizer/emitter ratio influence (Table S1 and Figure S3). Increasing the amount of the emitter in the CPNs yielded a higher UC/Ph ratio, possibly due to a more favored TTA process. However, above a certain **DPA-S-COOH** amount, the UC/Ph ratio decreased, which we ascribed to an excessive aggregation of the emitter molecules, causing the emission quenching (vide infra). Among all of them, **VVUC1-CPNs** obtained from an initial **DPA-S-COOH/Pd-S-COOH** = 1281 ratio (Table S1, entry 3) yielded reproducibly the highest UC-to-phosphorescence integrated intensity ratio (6.54) and little residual phosphorescence, suggesting a quite highly efficient sensitizer-to-emitter TTET (Figure 2c). In fact, nearly pure blue-color fluorescence of the **VVUC1-CPNs** suspension could be detected by the naked eye upon irradiation with a CW 532 nm laser (Figure 2d). Noticeably, even after prolonged irradiation time (30 min) at a relatively high power density (1.2 W/cm^2^), the UC intensity did not suffer any decrease, suggesting very good photostability of the CPNs (Figures 2e and S4), which also preserved their spherical nanostructuration (Figure 2f).

Due to the good UC properties, these nanoparticles were further characterized. First, relevant information gathered about this batch of nanoparticles is that they can be easily resuspended in Milli-Q water with good colloidal stability, without forming aggregates at least for 24 h, as confirmed by dynamic light scattering (DLS) analysis (60.0 ± 23.0 nm) over this period of time (Figure S5). Fourier transform infrared spectroscopy (FT-IR) showed a band at 1650 cm^–1^ and another medium intensity band at 1371 cm^–1^; the intensity and frequency of the bands indicate that they correspond to νCO_asym_ and νCO_sym_ vibrations, respectively. The former is shifted from 1705 cm^–1^ and the latter from 1440 cm^–1^, observed in **DPA-S-COOH**, or from 1688 and 1441 cm^–1^ of **Pd-S-COOH** free ligands, indicating the coordination of the carboxyl groups of the ligands to the metal center (Figure S6). X-ray powder diffraction (XRD) showed the absence of diffraction peaks, which confirms the amorphous nature of the solid nanoparticles. This corroborates that the 5-carbon alkyl chain is a sufficient length to break the crystalline order (Figure S7).

The efficiency of the TTA-UC process is marked by the excitation threshold intensity (*I*_th_) parameter, which indicates the excitation power density at which TTA becomes the main decay channel and the quantum yield of UC (Φ_UC_) is maximized.^26,53^ In our case, an *I*th value of 90 mW·cm^–2^ was found from the logarithm plot of the integrated UC intensity against the incident light power density (intersection point between the fitting lines of the quadratic—at low intensities—and linear—at higher intensities—trends, see Figure S8). It must be mentioned, though, that the accurate definition of this value is not straightforward in these systems, as differently from homogeneous solutions, data might be affected by the scattering provided by the suspended nanoparticles, which prevents the exact evaluation of *I*th. Several measurements carried out with different equipment and using CPN concentrations that minimized light scattering and self-absorption, while providing enough UC emission to be detected, allowed us to estimate such a value of *I*th that is in the range of those reported for other TTA-UC solid materials,^20,21,54^ comparable to the light intensity of 1 sun, and much lower than the power density required for lanthanide-based bulk materials and UCNPs.^55^ At this power density and considering a maximum Φ_UC_ of 50% due to the bimolecular character of the UC process, a final relative Φ_UC_ of 0.65% (against Ru(bpy)_3_Cl_2_, Φ_F_ = 0.04 in H_2_O)^56^ was measured.^25,26,57^ Noticeably, UC emission was observed in these CPNs despite the sensitizer and emitter molecules being in lower molar amounts than the Zr ion. This result evidences the good approach of amorphous CPNs. The flexibility of the polymeric chain allows the intra/interchain π–π interactions between the aromatic moieties of the chromophores and thus the proximity to favor the TTET and TTA through triplet migration (compensating the lack of dye diffusion in the CPNs), which finally yield the UC emission.

It is worth mentioning that this value most likely is modulated downward by light scattering and DPA aggregation (and self-quenching) due to the high dye density, which lowers the overall value of the UC. To bring more light to this fact, further spectroscopic data were obtained for these CPNs. The absorption spectrum of these **VVUC1-CPNs** was measured in water, at different concentrations, from 3.0 to 0.01 mg/mL (Figure S9). The most concentrated suspensions (1.0–3.0 mg/mL) reveal an absorption band at 524 nm, associated with **Pd-S-COOH**, despite its low concentration in the CPNs, as indicated by the final polymer molecular formula (**DPA-S-COOH**)_2900_(**Pd-S-COOH**)_1_Zr_5703_. The progressive increase of the absorption signal at higher energies, particularly in the most concentrated suspensions, was due to the light scattering provoked by the nanoparticles, which becomes stronger at higher concentrations and shorter wavelengths (Figure S9a,b). The lowest concentrated suspensions showed the characteristic vibronic progression of the absorption band of anthracene-based emitter molecules (**DPA-S-COOH**). Similar results (i.e., scattering effects and spectral shape of **DPA-S-COOH**, Figure S9c,d) were observed for CPNs made by only the emitter (**DPA-S-CPNs**, 45.5 ± 5.1 nm, by SEM image, Figure S10).

The normalized absorption spectrum of **DPA-S-COOH** in **VVUC1-CPNs** (1.0 mg/mL) was compared to the absorption spectra of the same dye in DMF solution and **DPA-S-CPNs** (1.0 mg/mL). Despite all of them showing the characteristic vibronic structure of **DPA-S-COOH**, the bands of **VVUC1-CPNs** and **DPA-S-CPNs** were red-shifted (λ_0–0_ = 400 nm), broadened, and less defined (the peak-to-valley ratio decreased) than the spectrum of the molecular dye in solution (Figure S11a), indicating the formation of ground-state aggregates of the emitter molecules in both types of CPNs. As expected, the full overlap of the spectra of **VVUC1-CPNs** and **DPA-S-CPNs** suggests that the small amount of **Pd-S-COOH** molecules in the former does not affect the aggregation of the emitter compound. Worth to mention is the lack of variation of the normalized absorption spectra of **VVUC1-CPNs** and **DPA-S-CPNs** suspensions, measured at different concentrations, which shows that the spectral shape is independent of the dilution and that the aggregation is produced within the CPNs (Figure S11b,c). The same conclusions were extrapolated from the excitation spectra (recorded at λ_emi_ = 450 nm, Figure S12a) of **VVUC1-CPNs** and **DPA-S-CPNs**, broader, less defined (smaller peak-to-valley ratio), and more red-shifted than the spectrum of the molecular solution of the ligand. Finally, dye aggregation was confirmed from the slightly red-shifted emission (recorded at λ_exc_ = 375 nm, Figure S11b) of both types of CPN suspensions (λ_emi_^max^ = 440 nm), as well as from the reduction of the absolute **DPA-S-COOH** emission quantum yield (Figure S13) in **VVUC1-CPNs** (18.0–34.2%) and **DPA-S-CPNs** (18.5–37.0%), with respect to the free ligand solution (84.2%). These spectral analyses, especially those related to the absorption and excitation spectra, corroborate the presence of ground-state aggregates of the emitter molecules in the nanoparticles, though the presence of excimer species could not be discarded at this stage, as the emission spectra variation could be related to both ground- and excited-state aggregates.

We also analyzed the excitation and emission spectral features of **Pd-S-COOH** in **VVUC1-CPNs** (the absorption spectrum of **Pd-S-COOH** in the nanoparticles was too affected by the scattering) and compared them to those of the free ligand in solution and in CPNs made by only the sensitizer **Pd-S-CPNs**, synthesized as reference (64.6 ± 10.0 by SEM, Figure S14). The spectral shifts of the excitation and emission bands of **Pd-S-COOH** (λ_exc_^max^ = 533 nm) in both CPNs, compared to the spectrum of the free ligand, indicated that the **Pd-S-COOH** porphyrin molecules also undergo homo/heteromolecular aggregation within the nanoparticles (Figure S15).

Finally, by nanoseconds laser spectroscopy, we investigated the lifetime of the UC emission. Interestingly, the pulsed laser irradiation at 532 nm produced a UC signal, with practically no rise detectable in the monitored timescale, possibly due to a very fast TTET from the donor to the acceptor, which later decays very rapidly, mostly within the first 50 ns (Figure S16).

These results indicate that the nanostructuration of coordination polymers made of the sensitizer and emitter monomers allows for the TTET process in the diffusion-limited solid state, which is required for the UC to occur. In the future, in-depth studies will be carried out to accomplish more details on the types of aggregates formed within the CPNs and the phenomena regulating the efficiency of TTA and TTET. These studies will allow the design and proper modification of the structure of the emitter molecules to prevent their self-quenching, which should yield, in the last instance, higher UC quantum yield values.

### 2.2 Generality of the Strategy

To demonstrate the universality of our approach, we also designed and synthesized nanoparticles upconverting light in a different spectral range and absorbing at lower energies (>600 nm), of interest for different applications (**VVUC2-CPNs**). For this, we aimed to use sensitizers forming their triplets upon direct singlet-to-triplet (S–T) absorption, without passing through the ISC that competes unfavorably with vibrational relaxation modes, especially for low-energy singlet excited states (S_1_).^58−61^ The monomers of choice for this were the terpyridine osmium complex functionalized with succinic acid attached via an ester bond (**Os-S-COOH**) (sensitizer) and the 4-((10-(4-carboxyphenyl)-anthracene-9-yl)-ethynyl)-benzoic derivative (emitter), chemically modified with a carboxylic acid-terminated alkyl chain via an amide bond (**CAEBD-S-COOH**) (Figure 1c). The synthetic strategy and the chemical characterization of the final products and the intermediates are shown in the SI (Sections S4 and S5). Overall, the sensitizer and the emitter were obtained in 2 (12.6% yield) and 7 (23.4%) steps from the initial commercial compounds, respectively. In DMF, **Os-S-COOH** showed absorption bands at λ_max_ = 490 and 680 nm (with the tail reaching 730 nm) and phosphorescence at λ_max_ = 773 nm (1.60 eV), while **CAEBD-S-COOH** absorption and fluorescence bands were observed at λ_max_ = 410 nm (with the λ_0–0_ band = 436 nm, 2.84 eV) and 460 nm, respectively (Figure S17). Last but not least, irradiation of a degassed DMF solution of the sensitizer (5 × 10^–4^ M) and the emitter (10^–3^ M) with a CW 650 nm laser (1.91 eV) yielded the upconverted emission at λ_max_ = 475 nm (2.61 eV) and an anti-Stokes shift of Δ*E*_UC_ = 0.67 eV (Figure S18). From the driving force of the TTA and TTET processes, we estimated the triplet energy of the emitter in the range of 1.42–1.60 eV, matching with the values reported in the literature for CAEBD derivatives (1.43–1.49 eV), determined experimentally or by TD-DFT calculations.^61,62^

**Figure 3:**
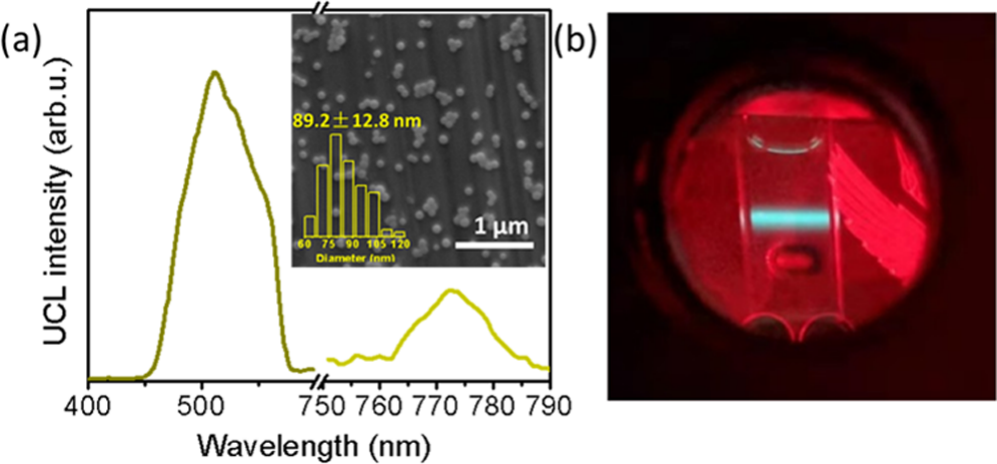
(a) Emission spectrum, SEM image (inset), and (b) photograph of **VVUC2-CPNs** aqueous suspension (λ_exc_ = 650 nm) obtained from **CAEBD-S-COOH/Os-S-COOH** = 120 (Table S2, entry 2). A short-pass filter (λ_cut-off_ = 650 nm) was used.

Considering these promising results, **VVUC2-CPNs** were synthesized through the reaction of **Os-S-COOH** and **CAEBD-S-COOH**, using different initial ratios, with zirconium oxychloride in DMF, employing the same experimental conditions as for **VVUC1-CPNs**. The results are summarized in Table S2. Irradiation of Milli-Q water suspensions of different **VVUC2-CPNs** with a CW 650 nm laser, under a N_2_ atmosphere, resulted in all cases of UC emission with λ_max_ = 511 nm, with different intensity ratios compared to the Os phosphorescence (Figures 3a and S19 and Table S2). After detailed analysis, **VVUC2-CPNs**, of size 89.2 ± 12.8 nm by SEM (Figure 3a, inset and S20), obtained from a **CAEBD-S-COOH/Os-S-COOH** = 120 ratio (Table S2, entry 2), and with molecular formula (**CAEBD-S-COOH**)_89_(**Os-S-COOH**)_1_Zr_235_, showed the highest integrated emission intensity ratio. The UC emission (detectable by the naked eye upon irradiation with a CW 650 nm laser, Figure 3b) was also stable over 30 min of strong irradiation with a CW 650 nm laser (6.6 W/cm^2^, Figure S21), after which the CPNs preserved their spherical shape and size (Figure S22).

Also in this case, the amorphous structure of the polymer promotes π–π interactions between the aromatic portions of the chromophores located in the same or different polymeric chains. These interactions ultimately allow TTET and TTA processes (via triplet migration) to occur in the solid CPNs, originating the final UC emission.

Again, the UC emission of **CAEBD-S-COOH** in the CPNs is slightly red-shifted compared to the dye solution fluorescence, possibly due to the dye aggregation. A detailed spectroscopic study was also carried out for these CPNs. The absorption spectra of **Os-S-COOH** in **VVUC2-CPNs** could not be detected even at high CPN concentrations, due to the combination of light scattering, a low amount of the Os complex, and its lower extinction coefficient (2.12 × 10^4^ M^–1^ cm^–1^) due to the less favored S–T absorption (Figure S23). The absorption spectra of **VVUC2-CPNs** and the reference CPNs made by only the emitter (**CAEBD-S-CPNs**, 82.8 ± 11.3 nm, by SEM, Figure S24) were significantly broader and less defined than the spectral band of the monomeric free ligand in solution (Figure S25a). The presence of such aggregates in the CPNs was also manifested by the large bathochromic shift, compared to the free ligand, of the **CAEBD-S-COOH** emission and the absolute fluorescence emission quantum yield of the CPNs (Φ_F_ = 3.0%), significantly lower than the free ligand (Φ_F_ = 57.3%). The emission red shift in **VVUC2-CPNs** (λ_emi_^max^ = 510 nm) was observed upon both direct excitation and through delayed UC (Figure S25b), indicating that the emission derives from the same emitter species. The detected little spectral difference was ascribed to self-absorption of the higher energy photons, observed in the UC experiments.

### 2.3 Air-Stable UC Printed Patterns and Transparent Films Based on UC-CPNs

Once the synthesis of UC-CPNs for different spectral ranges was achieved, we aimed to explore their use for practical applications. Their low dimensions (<100 nm) and narrow size distribution prevent light scattering, allowing us to explore first the fabrication of nanoparticles-containing highly transparent polymeric films, as demonstrated for other low-size oil photochromic nanodroplets^63^ and fluorescent solid lipid nanoparticles.^64^ These UC transparent polymeric films could be of interest to OLEDs, solar cells, and luminescent solar concentrators.^12,13,27,28^ As a proof of concept for this, we explored the water-soluble poly(vinyl alcohol) (PVA) as a matrix because (i) it is an excellent film-forming polymer^63−67^ and (ii) it has been shown as one of the most efficient oxygen barrier films (which prevents the triplet quenching).^59,68^ The colloidal stability of our CPNs in water allowed us to integrate molecular UC materials in such a performing polymer, otherwise impossible for hardly water-soluble UC organic dyes.

**Figure 4:**
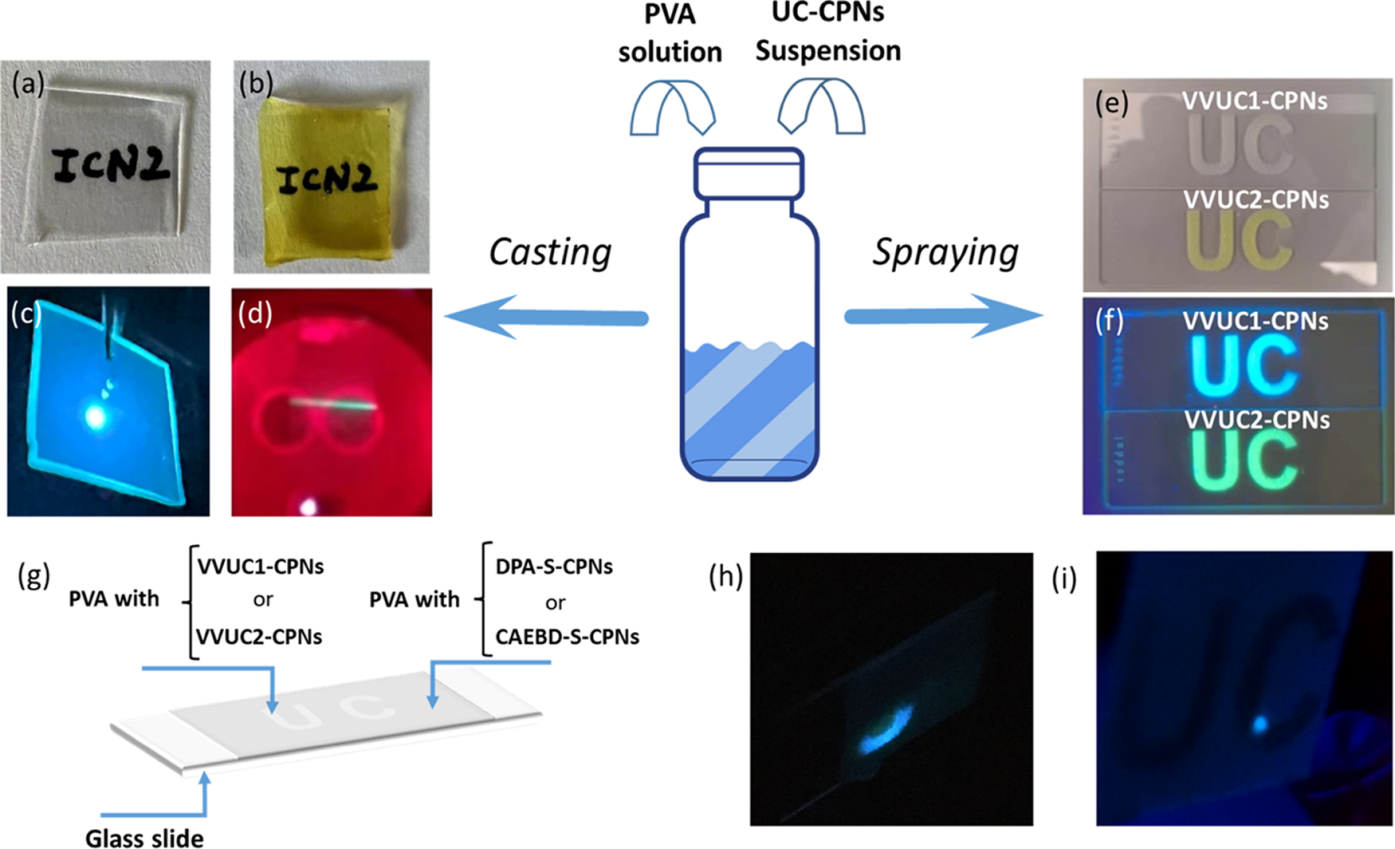
Photographs of transparent (a) **VVUC1-CPNs@PVA** and (b) **VVUC2-CPNs@PVA** films under ambient light; photographs of (c) **VVUC1-CPNs@PVA** and (d) **VVUC2-CPNs@PVA** films showing UC emission under 532 and 650 nm CW laser irradiation, respectively; photographs of **VVUC1-CPNs** and **VVUC2-CPNs** spray-coated patterns under (e) ambient light and (f) UV irradiation (λ_exc_ = 365 nm); (g) scheme of the preparation of the material with a UC-based encrypted message; screenshots of the videos showing the UC emission of the spray-coated (h) **VVUC1-CPNs**- and (i) **VVUC2-CPNs**-based patterns while scanning with 532 nm (pulsed Nd:YAG) and 650 nm (CW) laser irradiation, respectively. UC emission only appears when the laser beam scans over the “UC” pattern. The beam spot of the 532 nm pulsed laser is large enough (1 cm diameter) to cover a surface area with and without **VVUC1-CPNs**, developing UC emission selectively in the area patterned with the UC nanoparticles (e.g., the bottom part of the “U” of the “UC” pattern as shown in Figure 4h).

PVA films were then easily prepared by drop-casting in a mold **VVUC1-CPNs** or **VVUC2-CPNs** suspensions containing PVA and letting the water evaporate at room temperature. Flexible, free-standing, and highly transparent films could be pealed out from the container (Figures 4a,b and S26). Thanks to not only their small dimensions but also their homogeneous distribution within the polymer, the resulting films showed high transparency (%*T*_555 nm_ = 84 and 75%*T* against air, respectively, Figure S27). Most importantly, **VVUC1-CPNs@PVA** and **VVUC2-CPNs@PVA** films showed blue and green emissions, respectively, upon irradiation with 532 and 650 nm CW lasers, under an air atmosphere, confirming the generation of air-stable UC photons (Figure 4c,d). From here, three facts deserve to be emphasized. First, the UC/phosphorescence ratio of **VVUC1-CPNs** was even higher than the corresponding colloidal suspensions (Figure S28), possibly due to better protection from oxygen quenching by PVA, which prevents the need for additional protective coatings, e.g., nanocellulose, or antioxidants, normally used to inhibit oxygen diffusion and/or quenching of the triplet states.^22−24,69^ Second, UC was observable using a relatively low amount of CPNs (0.67 wt % with respect to PVA), without needing rubber-type polymers or high dye concentrations (as normally happens in UC polymeric matrices). Indeed, the structure of the CPNs minimizes dye migration and phase separation and assures that the high local concentration of the dyes within a particle is maintained once embedded in the final film. Third, the photostability study of the films in aerated conditions showed that the UC emission not only could be preserved during long irradiation times (30–40 min) at strong power densities (1.2 mW/cm^2^, 532 nm and 6.6 mW/cm^2^, 650 nm respectively) but also increased up to a photostationary state (Figure S29). Such enhancement was ascribed to the consumption of O_2_ present in the films, which was trapped during their fabrication or slowly diffused inside over time. At the beginning of irradiation, residual oxygen quenches the photoinduced triplet states of the dye molecules and thus the overall UC, forming ^1^O_2_ or other reactive oxygen species. With time, these species irreversibly react with the dyes of the CPNs, which consume the residual quenching oxygen source. When all oxygen is consumed, the quenching process is inhibited and UC intensity quickly increases. The UC enhancement over time indicates that (i) the amount of dye molecules irreversibly degraded by the reactive oxygen species is negligible compared to the dyes remaining available for the UC process, and (ii) the oxygen consumption is far faster than the potential diffusion of further atmospheric air, due to the extremely good PVA oxygen barrier effect. Such a nanocomposite structure could also be extended to other natural polymers, e.g., cellulose, which was recently used to obtain stable and recyclable UC films in the air atmosphere, due to similar oxygen barrier properties.^70^

Finally, as a proof-of-concept application, we explored the use of these films as UC luminescent solar concentrators, as UC is desirable to avoid the typical dye reabsorption of re-emitted light during its traveling to the edges.^13,27,28^

Successfully, localized irradiation with a CW 532 nm laser at the center of the **VVUC1-CPNs@PVA** film yielded intense UC emission at the edges of the film (Figures 4c and S30). These results demonstrate that CPNs are small enough to minimize the scattering of light, which concentrates at the edges through total internal reflection.

The good colloidal dispersion of our nanoparticles in aqueous suspensions was also successfully used to prepare water-based security inks. Both **VVUC1-CPNs** and **VVUC2-CPNs** could be easily patterned, in the presence of the PVA binder, onto the glass substrate through spray-coating and using a prepatterned mask (with “UC” shape, Figure 4e), preserving their fluorescent properties under UV irradiation (λ_exc_ = 365 nm, Figure 4f) and UC emissions under 532 and 650 nm light (Figure S31). To prepare an encrypted label, a glass substrate was first spray-coated all over the surface with a suspension made of **DPA-S-CPNs** or **CAEBD-S-CPNs** (containing only **DPA-S-COOH** or **CAEBD-S-COOH**) and PVA, obtaining a uniform printed UV active substrate (background) with rough surfaces due to the PVA microcapsules formed upon spraying (Figure S32). On top of this, the PVA/**VVUC1-CPNs** or PVA/**VVUC2-CPNs** aqueous suspensions were spray-coated on the respective prepared substrate, interposing the prepatterned “UC” mask between the nozzle and the substrate (Figure 4g). Irradiation with UV light (λ_exc_ = 365 nm) over the samples showed the emission of **DPA-S-COOH** or **CAEBD-S-COOH**, present in both the background and the patterned material (Video S1). In this way, the “UC” label remains encrypted. On the other hand, when 532 and 650 nm CW lasers were used, the corresponding UC emissions were activated only in the regions where the CPNs were deposited (Figures 4h,i, S33, and 34). Videos S2–S4 show the scanning of the substrate surface with 532 and 650 nm CW lasers and a pulsed 532 nm beam. UC emission only appears when the beam scans over the “UC” pattern, decrypting the hidden message.

## 3 Conclusions

In summary, the introduction of an alkyl chain within emitter and sensitizer ligands allowed us to synthesize photostable UC amorphous nanoscale coordination polymers with high dye densities and easy dye ratio tunability. This approach prevents dye migration and phase segregation of the dyes in the polymer material (matrix) and allows the optimization of the dye interactions and the consequent energy transfer processes involved in TTA-UC. We proved that the approach can be generalized to upconvert photons from/to different spectral regions and through distinct mechanisms (intersystem-crossing or direct singlet-to-triplet absorption). Moreover, given their chemical, colloidal, and photostability in water, these CPNs could be considered the organic counterpart of the lanthanide-based inorganic UCNPs, but with lower excitation intensity thresholds. The system might be further improved through the addition of a bulky group in the **DPA-S-COOH** structure to reduce aggregation while preserving the high density in the CPNs to favor TTET and TTA. Finally, the combination with the water-soluble and oxygen barrier PVA polymer allowed us to obtain UC waterborne inks and transparent films where UC is preserved even at low CPN concentrations. The interest in these platforms was exemplified by the development of anticounterfeiting patterns and luminescent solar concentrators.

## 4 Experimental Section

### 4.1 Synthesis of UC-CPNs

Appropriate amounts of dyes (see Table S1 in Section 6, for the exact amounts and relative ratio of the sensitizer and the emitter) were dissolved in 2 mL of anhydrous DMF with final addition of ZrOCl_2_ (6 mg). The resulting mixture was stirred and heated at 90 °C for 15 h. The solid nanoparticles were isolated by centrifugation (14 600 rpm, 15 min). The centrifuged material was washed three times with DMF and two times with water to remove unreacted compounds.

### 4.2 Preparation of UC films

A 0.1 mL Milli-Q water suspension (10 mg/mL) of UC-CPNs obtained after the synthesis and purification via centrifugation was mixed with 1.5 mL of a 10 wt % PVA 4–88 solution while stirring to improve the dispersion. The mixture was poured into a mold (1.7 × 1.7 × 0.5 cm^3^), and the water was allowed to evaporate. Upon precipitation of the polymer, the film was formed.

### 4.3 Spray Coating of the CPNs onto a Glass Substrate

In total, 4 mg of UC-CPNs were suspended in 0.2 mL of Milli-Q water. The resulting suspension was then mixed with 2 mL of a 10 wt % PVA 4–88 solution while stirring to improve the dispersion. The mixture was sprayed through an adapted spray dryer (Buchi 191 spray dryer) onto a glass slide kept at a distance of 16 cm from the nozzle and using a prepatterned mask.

## Supporting Information Available

General characterization methods; synthesis and characterization of the dyes for the CPNs and intermediates to obtain them; general procedures for the preparation of CPNs; films containing the CPNs and the printing of the CPNs; and supplementary schemes, tables, and figures (PDF)
Scanning with a UV beam over the **VVUC1-CPNs**-based spray-coated pattern deposited onto the **DPA-S-CPNs** background. The emission does not change all over the scanning as **DPA-S-COOH** emission is always activated (MOV)
Scanning with the 532 nm CW laser over the **VVUC1-CPNs**-based spray-coated pattern deposited onto the **DPA-S-CPNs** background. The UC emission only appears when the beam crosses the “UC” pattern (MP4)
Scanning with the 650 nm CW laser over the **VVUC2-CPNs**-based spray-coated pattern deposited onto the **CAEBD-S-CPNs** background. The UC emission only appears when the beam crosses the “UC” pattern (MP4)
Scanning with the 532 nm Nd:YAG nanosecond pulsed laser over the **VVUC1-CPNs**-based spray-coated pattern deposited onto **DPA-S-CPNs**. The UC emission only appears from the “UC” pattern (MP4)

## Supplementary Material

am2c16354_si_001.pdf

am2c16354_si_002.mov

## Abbreviations

CPNs: coordination polymer nanoparticles
UC: upconversion
UC-CPNs: upconverting CPNs
**DPA-S-COOH**, **Pd-S-COOH**, **CAEBD-S-COOH**, and **Os-S-COOH**: 9,10-diphenylanthracene, palladium(II) meso-tetraphenylporphyrin complex, [4-((10-(4-carboxyphenyl)-anthracene-9-yl)-ethynyl)-benzoic derivative, and terpyridine Os complex, respectively, all functionalized with carboxylic acid-terminated spacers (S-COOH)
**VVUC1-CPNs** and **VVUC2-CPNs**: CPNs upconverting from different energies (532 and 650 nm, respectively) of the visible spectral region
**DPA-S-CPNs**, **Pd-S-CPNs**, and **CAEBD-S-CPNs**: CPNs made of **DPA-S-COOH**, **Pd-S-COOH**, and **CAEBD-S-COOH**, respectively
PVA: poly(vinyl alcohol)
**VVUC1-CPNs@PVA** and **VVUC2-CPNs@PVA**: PVA films containing **VVUC1-CPNs**, **VVUC2-CPNs**, respectively
CW: continuous wave
