# Supplementary material for: Water-Stable Upconverting Coordination Polymer Nanoparticles for Transparent Films and Anticounterfeiting Patterns with Air-Stable Upconversion
Source: ACS Appl Mater Interfaces. 2023 Feb 1;15(6):8377–86. doi: 10.1021/acsami.2c16354 (PMC9940112; doi:10.1021/acsami.2c16354)
Supplement: Supplementary file 1 — am2c16354_si_001.pdf [file am2c16354_si_001.pdf]

Supporting Information

Water-stable upconverting coordination polymer nanoparticles for transparent films and anticounterfeiting patterns with air-stable upconversion

Junda Zhang, Daniel Ruiz-Molina, Fernando Novio, Claudio Roscini**

Catalan Institute of Nanoscience and Nanotechnology (ICN2), CSIC and BIST Campus UAB, Bellaterra, 08193 Barcelona, Spain.

E-mail: claudio.roscini@icn2.cat; fernando.novio@icn2.cat

Table of content

Section 1. Materials and methods 2

Section 2. Synthesis and characterization data of DPA-S-COOH and intermediates to obtain it 5

Section 2. Synthesis and characterization data of Pd-S-COOH and intermediates to obtain it 14

Section 3. Synthesis and characterization data of CAEBD-S-COOH and intermediates to obtain it 26

Section 4. Synthesis and characterization data of Os-S-COOH and intermediates to obtain it 37

Section 5: Preparation of nanoparticles and nanocomposite materials... Error! Bookmark not defined.

Section 6: Supplementary Schemes, Tables, Figures and videos 41

Section 1. Materials and methods

1.1 Materials

All chemicals were purchased from Sigma Aldrich and used without further purification. MilliQ® water is obtained using A10TOC MilliPore (Merck).

1.2 Characterization

Fourier transform infrared (FTIR) spectra were carried out with a Tensor 27/PMA50FTIR Spectrometer in a range of 4000–400 cm^{-1}. ^{1}H-NMR, ^{13}C-NMR, DEPT135 were recorded in Bruker Avance NEO 360 MHz. Dynamic light scattering (DLS) for the determination of the particle-size distributions and the zeta potential values were measured in a ZetaSizer nano ZS (ZEN3600, Malvern Instruments, Ltd., Malvern, UK). Scanning Electron Microscopy (SEM) images were obtained on a scanning electron microscope (FEI Quanta 650 FEG, The Netherlands). The samples were casted on aluminum holders followed by evaporation and later a thin platinum layer was sprayed to increase the conductivity of the samples. Ultraviolet–visible spectroscopy (UV-vis) study was carried out in the Agilent Cary 60 spectrophotometer, using 1 cm optical path quartz cuvette. Emission and excitation spectra were performed in the fluorimeter PTI Quantamaster 300. Unless differently specified, 1 mg/mL UC-CPNs were dispersed in MilliQ® water in sealable quartz fluorescence cuvette (1 cm optical path) and the suspension was degassed with N_2 for 1.5 h for upconversion measurement. UC and phosphorescence emission were measured after 1.5 h degassing.

Absolute fluorescence quantum yield values were obtained through Hamamatsu C9920-02G fluorimeter using an integrating sphere, after degassing the UC-CPNs suspensions. Relative UC emission quantum yield values were obtained measuring the emission of the reference

$Ru(bpy)_3Cl_2$ (λ_{exc} = 532 nm, Φ_{UC} = 0.04)[1] and using the equation $\Phi_{UC} = \Phi_f (A_f/A)(I/I_f)(\eta/\eta_f)$, where A = absorption at 532 nm, I = integrated emission intensity, η = refractive index of medium. Since UC derives from a bimolecular process, the maximum Φ_{UC} is 50%.

Phosphorescence lifetimes in the μs range were measured with the photomultiplier of the fluorimeter PTI Quantamaster 300, using a pulsed Xe lamp as excitation source (50-200 Hz) and 5 μs as integration time. Shorter lifetimes were measured using a nanoseconds Quantum Brilliant B Nd-YAG laser coupled with a doubling crystal to achieve 532 nm pulsed irradiation. In these measurements phosphorescence and UC spectra were recorded with the Andor ICCD camera at different time delays from the laser pulse. The integrated spectra were then plotted against the time delay. The scattering signal was used to find the time t = 0.

Irradiation to induce UC was carried out using CNI LED - 532 nm CW laser, Quantum Brilliant B Nd:YAG nanosecond pulsed 532 nm laser and a 650 nm CW laser.

1.3 HPLC methodology for the quantification of monomers in the UC-CPNs

Analyses were performed using a HPLC Waters 2695 separation module coupled to a water 2487 UV-Vis detector (suitable for dual detection 280 369nm). The column used was a Restek® C-18 (250 mm x 4.6 mm). Eluent A was a 0.1% (v/v) TFA aqueous solution and eluent B was acetonitrile absolute (HPLC grade). Before the analysis, the RP column was preequilibrated using the starting conditions of the method (99 % A (v/v)) for 10 min, followed by a gradual decrease of A from 100% to 0% (v/v) in first 15 min and lasting 4 min. Then, mobile phase was raised to 100 % A (v/v) in 3 min and lasting 3 min. Finally, mobile phase was reset to the initial conditions (A:B) 100:0 (v/v) and stayed for 10 min to equilibrate for the next injection. The flow rate was set at 1.0 mL/min and the column temperature was kept at 25 °C. The detection wavelengths were 280 and 369 nm. This method was used for the calibration curve of **DPA-S-COOH** and **CAEBD-S-COOH** and the quantification of these in the UC-CPNs.

1.4 ICP-AES analysis

The total Zr and Pd present in the UC-CPNs was determined using Varian 710-OES instrument equipment to detect the metal through Inductively Coupled Plasma Atomic Emission Spectroscopy. Samples of dried CPNs were dissolved in aqua regia and incubated at 80 ºC for

48 h. After incubation, samples were diluted (estimated concentration of Zr, Pd and Os ranging 0-400 ppb) in 0.5 % (v/v) HNO_3 and injected into the instrument using a peristaltic pump from different tubes placed in the autosampler. Calibration curves of Zr, Pd and Os were prepared with five different concentrations (0, 0.1, 0.5, 10, 100 and 200 ppb) by diluting certified reference metal ion solutions. The concentration of Zr and Pd present in samples were calculated from the corresponding calibration curve, previously adjusted to a linear regression model (R^2>0.99).

Section 2. Synthesis and characterization data of DPA-S-COOH and intermediates to obtain it

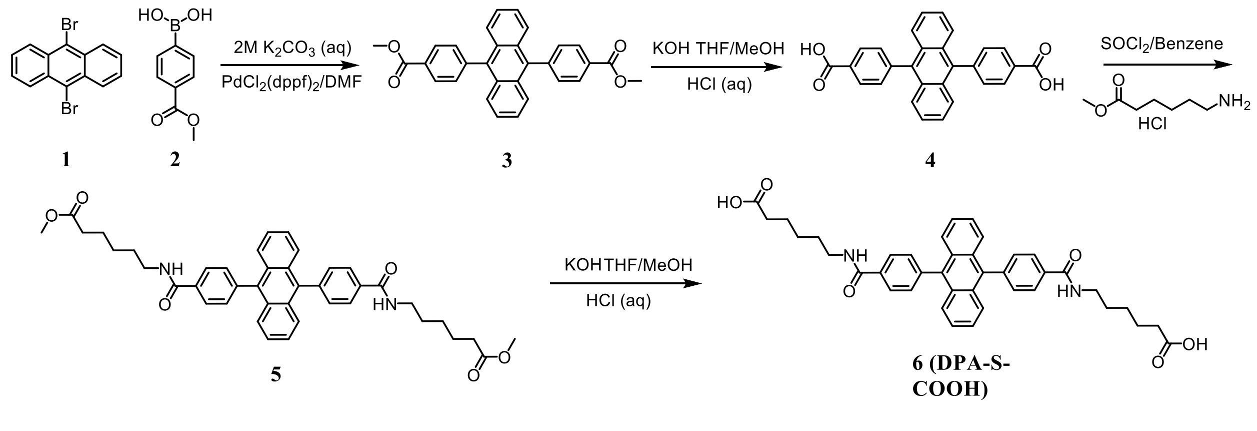

Synthetic route of **6** (**DPA-S-COOH)**

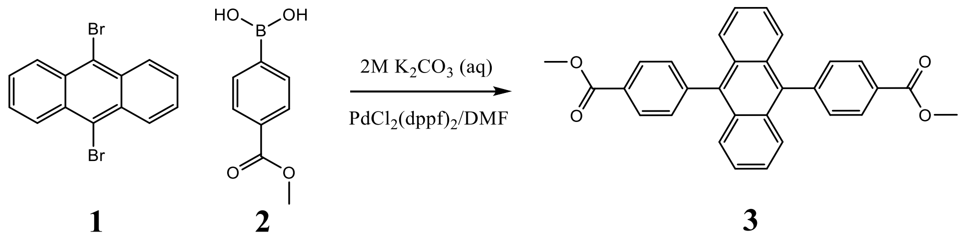

The mixture of **1** (9,10-dibromoanthracene, 250mg, 0.74mmol), **2** (4 - (methoxycarbonyl) phenyl) boronic acid, 335mg, 1.86mmol) and a catalytic amount of $PdCl_2(dppf)_2$ (54 mg, 0.074mmol) were dissolved in 20mL of degassed DMF. Then an aqueous K_2CO_3 solution (10 mL, 2 M) was added to the mixture and the reaction was heated to 155 ℃ for 22 h until the anthracene was consumed. The crude product was purified by column chromatography using CH_2Cl_2 to afford the molecule **3** as a white solid (224mg, 67.7 %).

^{1}H NMR (360 MHz, $CDCl_3$) δ: 8.35 – 8.23 (m, 4H, $H^{3,6,11,14}$), 7.60 (ddd, J = 8.2, 6.6, 2.4 Hz, 8H, $H^{15,16,18,19,21,22,24,25}$), 7.35 (dd, J = 6.9, 3.3 Hz, 4H, $H^{1,2,12,13}$), 4.03 (s, 6H, $H^{33,34}$).

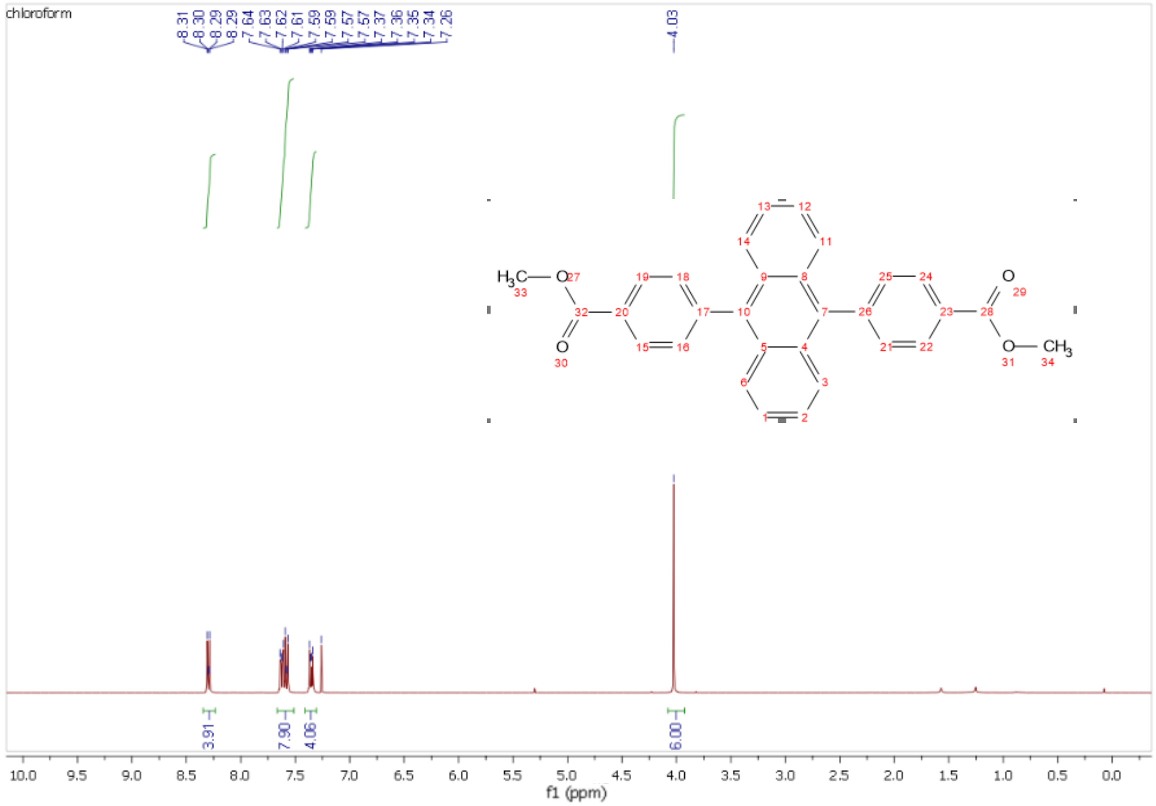

^1H NMR spectrum of **3**

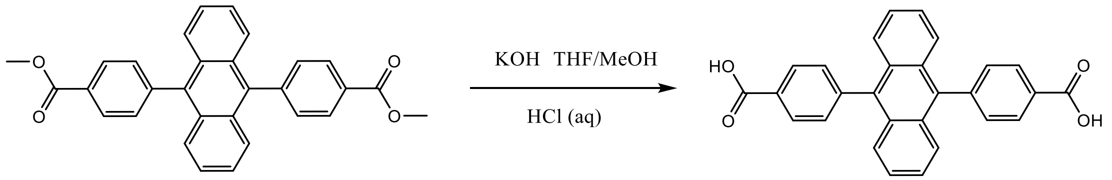

3 **4**

The 9,10-(methyl-4-carboxyphenyl) anthracene (217mg, 0.48mmol) was suspended in MeOH (15 mL), 1,4-dioxane (15 ml) and KOH aqueous (2M, 15 ml). The mixture was refluxed at 60 ℃ for 20 h. After cooling at room temperature, the mixture pH was changed to pH = 2 using hydrochloric acid (4 M). The precipitate was collected by filtration affording the molecule **4 (**DPA-COOH) as a white solid (183 mg, 90.1%)

^1H NMR (360 MHz, $(CD_3)_2SO$) δ: 13.17 (s, 2H, $H^{30,31}$), 8.22 (d, J = 8.2 Hz, 4H,$H^{3,6,11,14}$), 7.61 (d, J = 8.2 Hz, 4H, $H^{15,19,22,24}$), 7.58 – 7.52 (m, 4H, $H^{16,18,21,25}$), 7.48 – 7.42 (m, 4H, $H^{1,2,12,13}$).

^{13}C NMR (91 MHz, $(CD_3)_2SO$) δ: 167.67 (s, $C^{28,32}$), 143.30 (s, $C^{17,26}$), 136.43 (s, $C^{7,10}$), 131.75 (s, $C^{4,5,8,9}$), 130.76 (s, $C^{15,19,22,24}$), 130.10 (s, $C^{3,6,11,14}$), 129.30 (s, $C^{20,23}$), 126.66 (s, $C^{16,18,21,25}$), 126.38 (s, $C^{1,2,12,13}$).

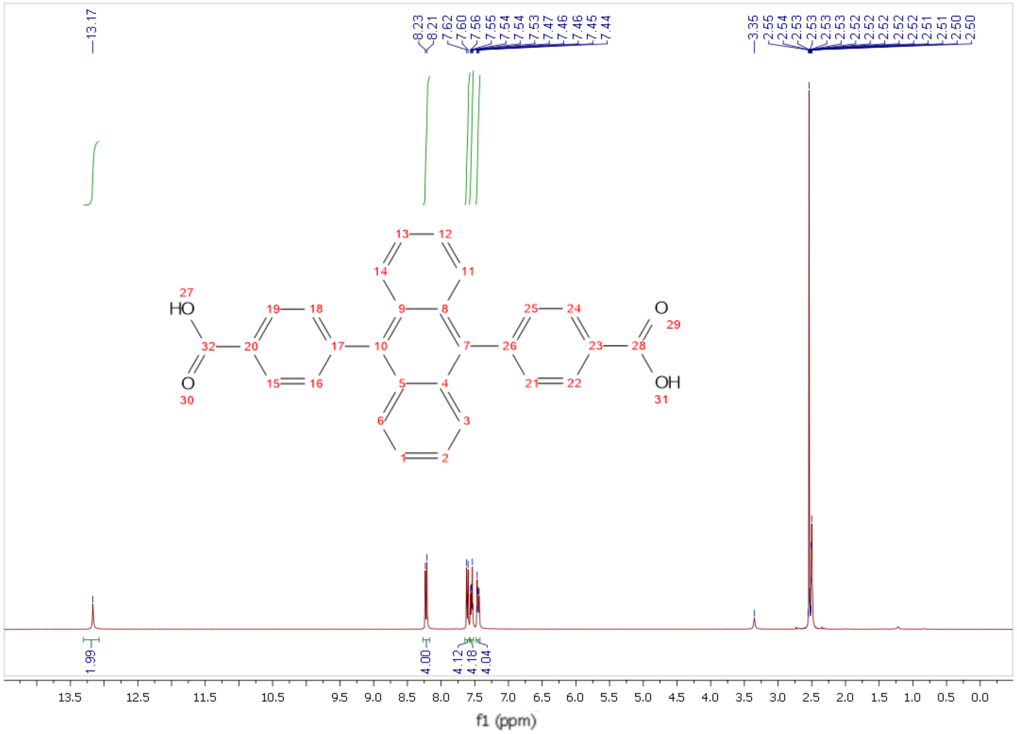

^{1}H NMR spectrum of **4**

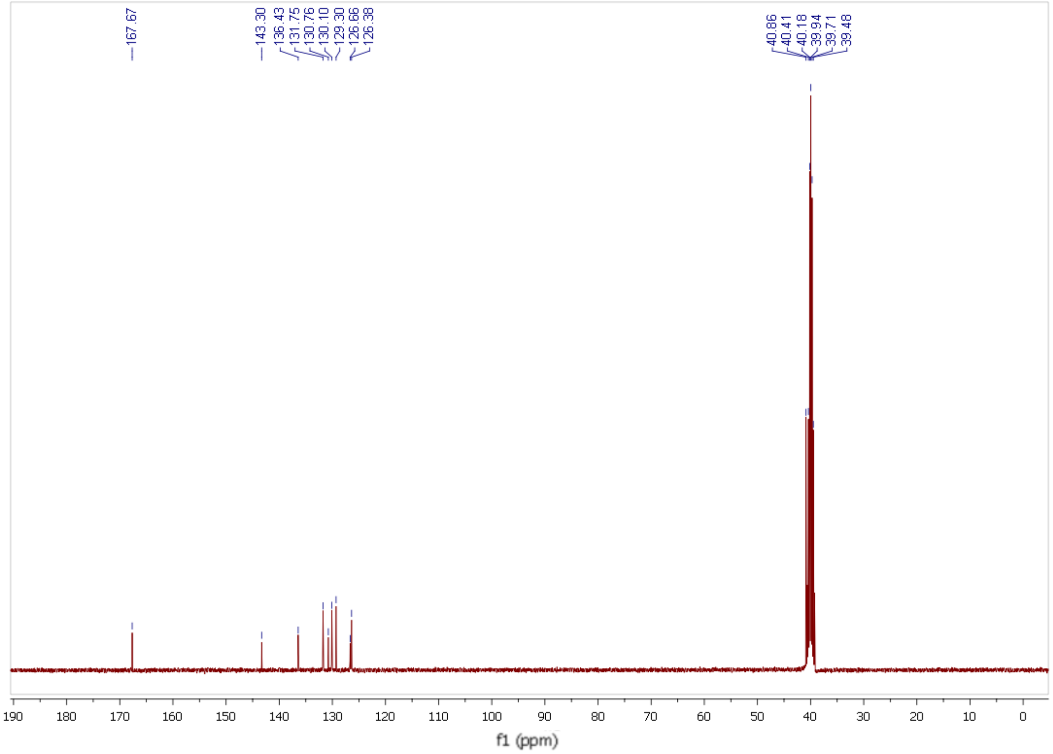

^{13}C NMR spectrum of **4**

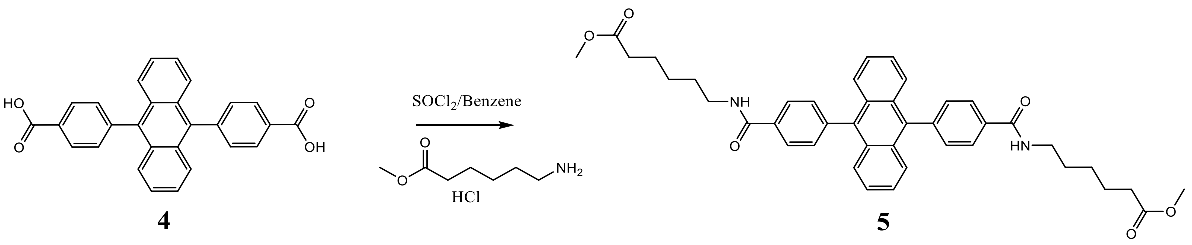

Compound **4** (4,4'-(anthracene-9,10-diyl) dibenzoic acid, 70 mg, 0.167 mmol) was placed in 10 mL 2-necked flask and benzene (5 mL) was added under N_2 atmosphere. Then thionyl chloride was added to the suspension and refluxed for 5 h with catalytic quantity of anhydrous DMF. After removal of redundant thionyl chloride and benzene, the residue was dilute by dehydrated CH_2Cl_2 and used directly for the next step affording the molecule **5** as a white solid (85 mg, 75.9%).

^{1}H NMR (360 MHz, $CDCl_3$) δ: 8.02 (d, J = 8.2 Hz, 4H, $H^{3,6,11,14}$), 7.63 (dd, J = 6.8, 3.3 Hz, 4H, $H^{15,19,22,24}$), 7.55 (d, J = 8.1 Hz, 4H, $H^{16,18,21,25}$), 7.33 (dd, J = 6.9, 3.2 Hz, 4H, $H^{1,2,12,13}$), 6.42 (t, J = 5.4 Hz, 2H, $H^{27,47}$), 3.68 (s, 6H, $H^{49,50}$), 3.57 (dd, J = 13.1, 6.8 Hz, 4H, $H^{37,39}$), 2.38 (t, J = 7.3 Hz, 4H, $H^{33,43}$), 1.77 – 1.69 (m, 8H, $H^{34,36,40,42}$), 1.57 – 1.44 (m, 4H, $H^{35,41}$).

^{13}C NMR (91 MHz, $CDCl_3$) δ: 174.16 (s, $C^{30,46}$), 167.43 (s, $C^{28,48}$), 142.38 (s, $C^{17,26}$), 136.30 (s, $C^{7,10}$), 134.09 (s, $C^{20,23}$), 131.58 (s, $C^{4,5,8,9}$), 129.56 (s, $C^{16,18,21,25}$), 127.09 (s, $C^{3,6,11,14}$), 126.66 (s, $C^{15,19,22,24}$), 125.40 (s, $C^{1,2,12,13}$), 51.60 (s, $C^{49,50}$), 39.84 (s, $C^{37,39}$), 33.87 (s, $C^{33,43}$), 29.36 (s, $C^{36,40}$), 26.43 (s, $C^{35,41}$),24.42 (s, $C^{34,42}$).

DEPT 135 (91 MHz, $CDCl_3$) δ: 131.58 (s, $C^{16,18,21,25}$),128.07 (s, $C^{3,6,11,14}$),126.66 (s, $C^{15,19,22,24}$),125.40 (s, $C^{1,2,12,13}$),51.59 (s, $C^{49,50}$),39.83 (s, ^{37,39}), 33.86 (s, $C^{33,43}$), 29.35 (s, $C^{36,40}$), 26.42 (s, $C^{35,41}$), 24.42 (s, $C^{34,42}$).

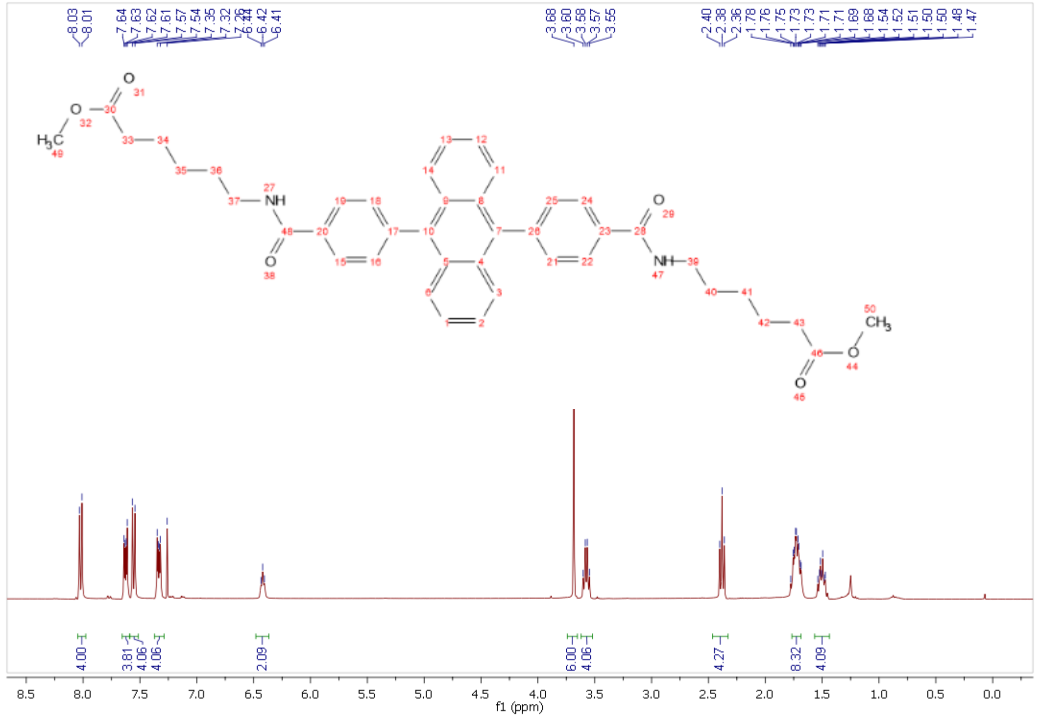

^{1}H NMR spectrum of **5**

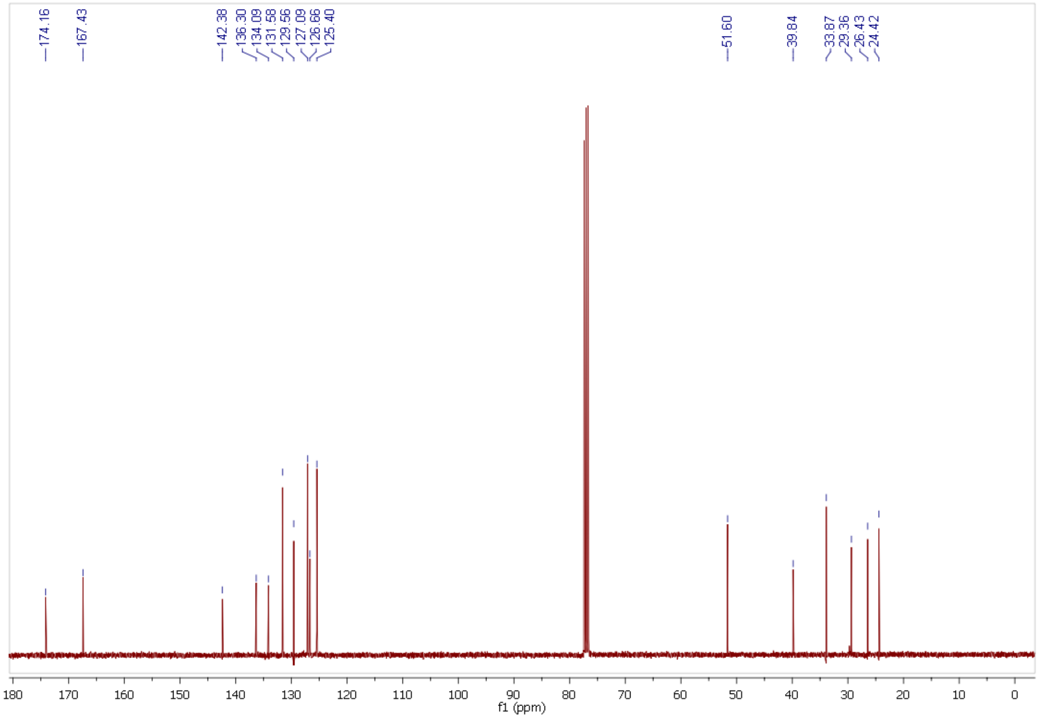

^{13}C NMR spectrum of **5**

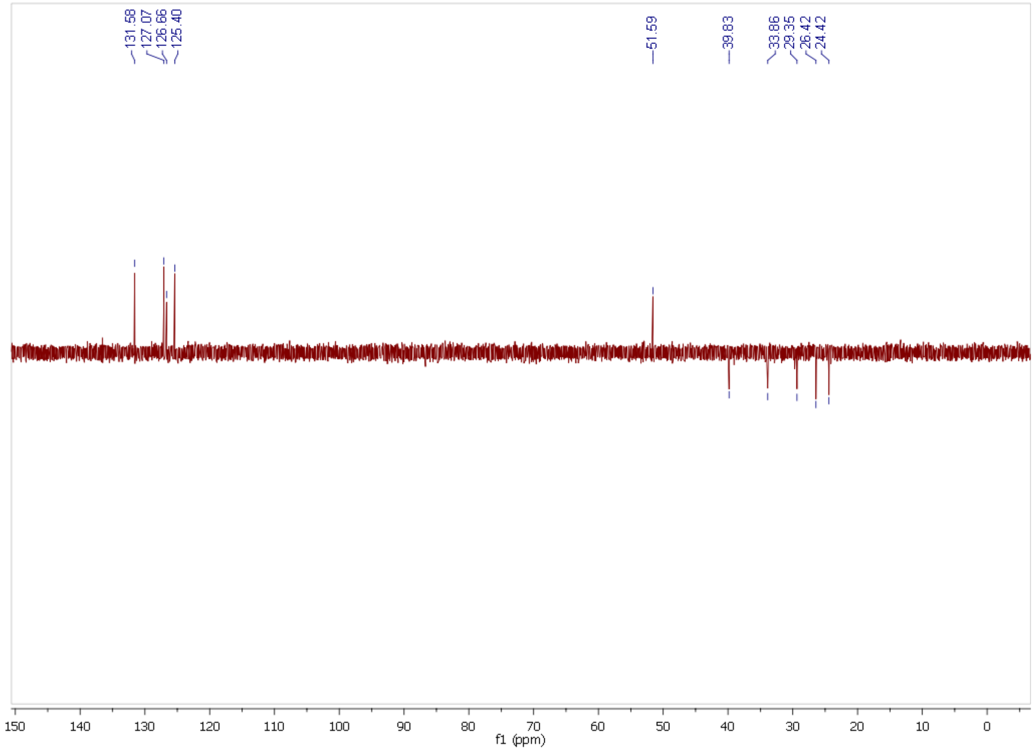

DEPT135 spectrum of **5**

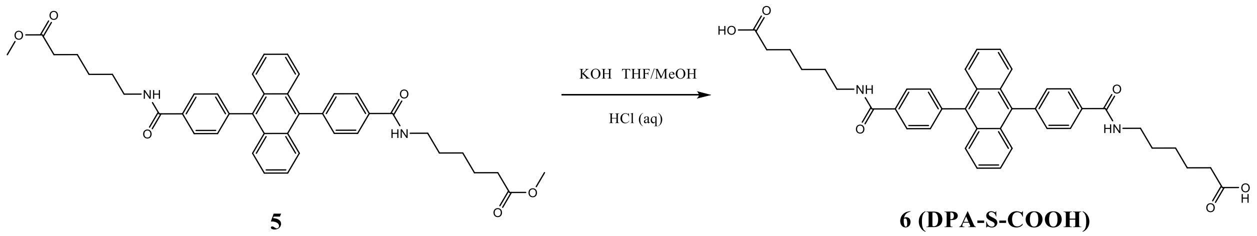

5 **6 (DPA-S-COOH)**

The compound **5** (85 mg, 0.13 mmol) was suspended in MeOH (15 mL), 1,4-dioxane (15ml) and KOH aqueous (2M, 15 mL). The mixture was refluxed at 60 ℃ for 20h. After cooling to room temperature, the mixture was tuned to pH = 2 using hydrochloric acid (4M). The precipitate was collected by filter affording the molecule **6** as a white solid (72.5 mg, 89.0%).

^{1}H NMR (360 MHz, *N, N*-dimethyl-formamide-d$_7$) δ: 12.36 (s, 2H, $H^{32,44}$), 8.75 (t, J = 5.3 Hz, 2H, $H^{27,47}$), 8.30 (d, J = 8.1 Hz, 4H, $H^{3,6,11,14}$), 7.72 – 7.60 (m, 8H, $H^{15,19,22,24,16,18,21,25}$), 7.51 (dd, J = 6.9, 3.1 Hz, 4H, $H^{1,2,12,13}$), 3.51 (dd, J = 12.6, 6.5 Hz, 4H, $H^{37,39}$), 2.36 (t, J = 7.3 Hz, 4H, $H^{33,43}$), 1.71 (dt, J = 15.3, 7.6 Hz, 8H, $H^{34,36,40,42}$), 1.57 – 1.43 (m, 4H, $H^{35,41}$).

^{13}C NMR (91 MHz, *N, N*-dimethyl-formamide-d$_7$) δ: 174.53 (s, $C^{30,46}$), 166.18 (s, $C^{28,48}$), 141.54 (s, $C^{17,26}$), 136.58 (s, $C^{7,10}$), 134.63 (s, $C^{20,23}$), 131.24 (s, $C^{4,5,8,9}$), 129.54 (s, $C^{16,18,21,25}$), 127.70 (s,

$C^{3,6,11,14}$), 126.54 (s, $C^{15,19,22,24}$), 125.82 (s, $C^{1,2,12,13}$), 39.58 (s, $C^{37,39}$), 35.08 (s $C^{33,43}$), 29.95 (s, $C^{36,40}$) 26.57 (s, $C^{35,41}$), 24.76 (s, $C^{34,42}$).

DEPT 135 (91 MHz, $CDCl_3$) δ: 131.24 (s, $C^{16,18,21,25}$),127.70 (s, $C^{3,6,11,14}$),126.54 (s, $C^{15,19,22,24}$),125.82 (s, $C^{1,2,12,13}$), 39.58 (s,^{37,39}), 33.76 (s, $C^{33,43}$), 29.40 (s, $C^{36,40}$), 26.57 (s, $C^{35,41}$), 24.77 (s, $C^{34,42}$).

MS-ESI (*m/z*): $[M]^-$ Calcd. for $C_{40}H_{40}N_2O_6^-$ 643.3, found 643.3.

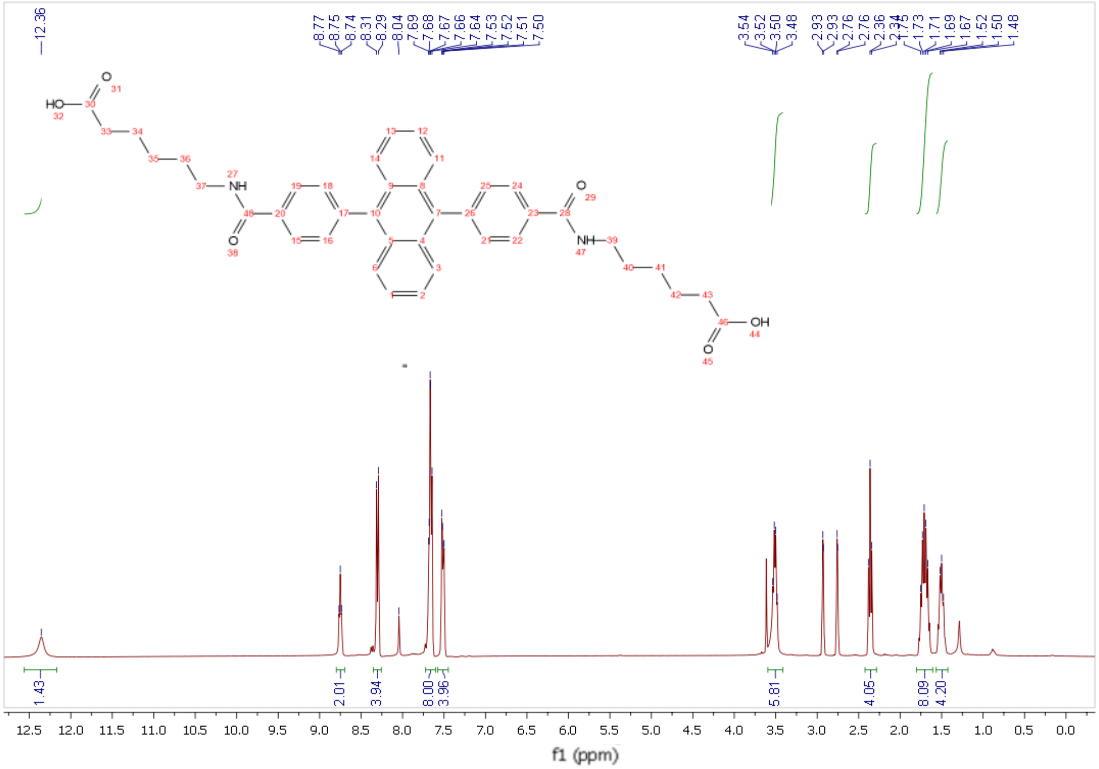

^1H NMR spectrum of **6** (**DPA-S-COOH**)

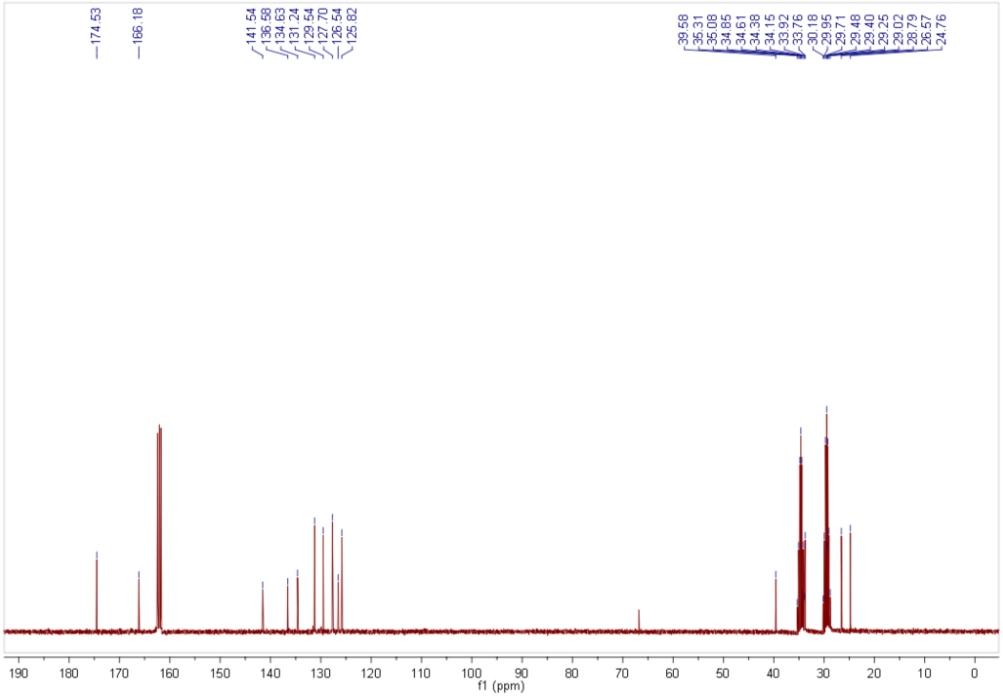

^{13}C NMR spectrum of **6** (**DPA-S-COOH**)

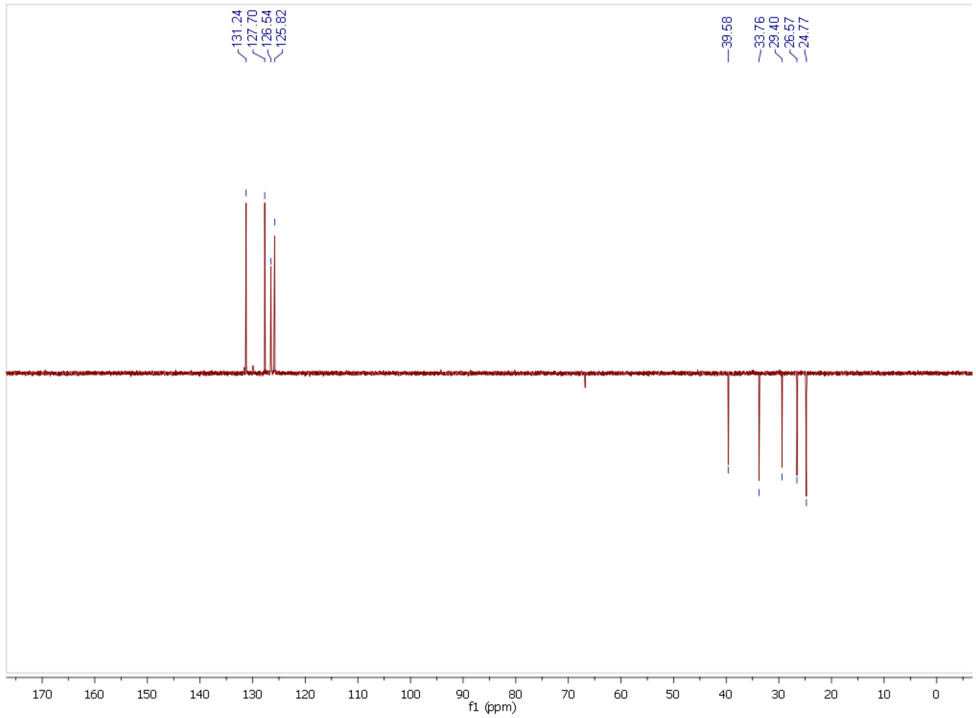

DEPT135 spectrum of **6** (**DPA-S-COOH**)

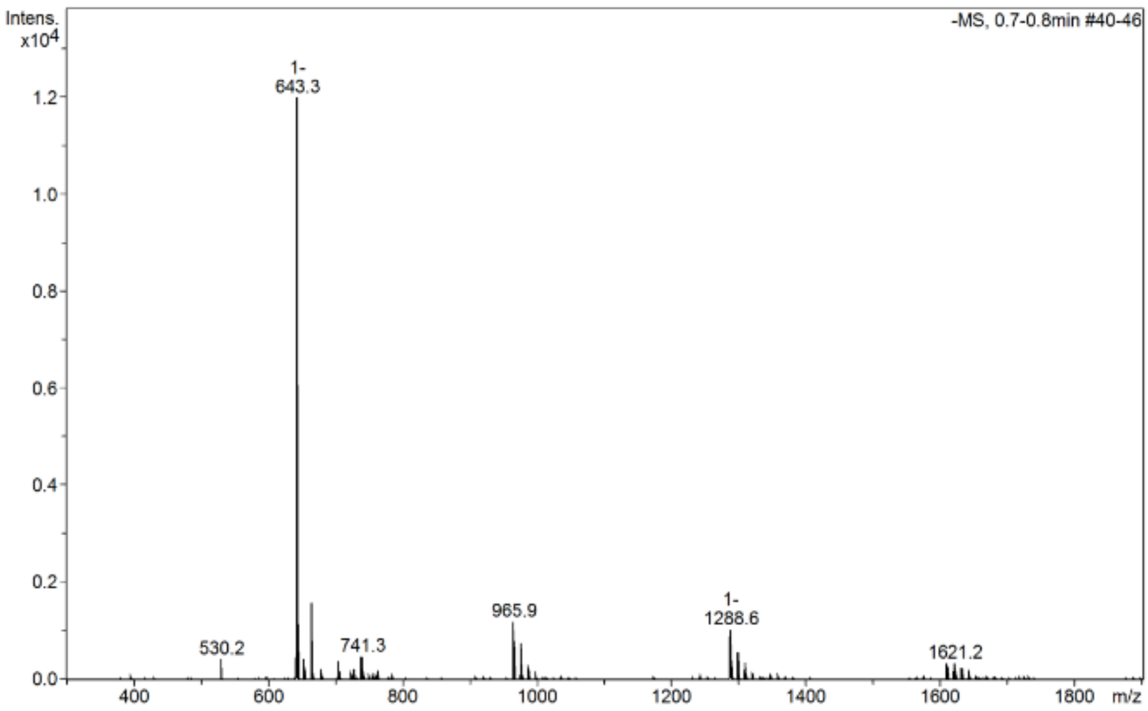

Mass spectrometry of **6** (**DPA-S-COOH**)

Section 2. Synthesis and characterization data of Pd-S-COOH and intermediates to obtain it

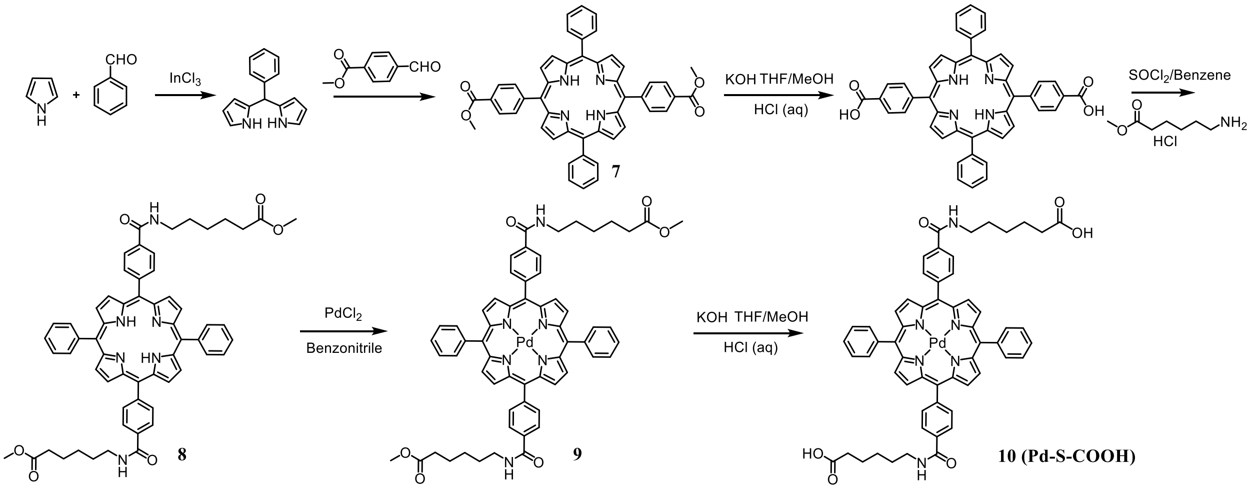

Synthetic route of **10 (Pd-S-COOH)**

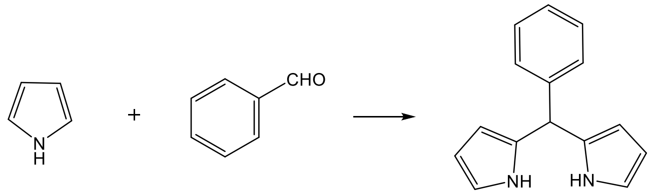

A mixture of benzaldehyde (2.0 g, 18 mmol) and pyrrole (30 mL, 0.44 mol) was treated with $InCl_3$ (0.2 g, 0.9 mmol) at room temperature under N_2 protection. After stirring for 6 h, NaOH (4.0 g, 0.1 mol) was added and the reaction mixture was stirred for 45 min. Excess of NaOH was filtered and excess of pyrrole was removed by distillation under reduced pressure. The resulting black solid was purified by silica gel column chromatography with CH_2Cl_2 to afford a dark-brown solid dipyrroliphenylmethane (3.2 g, 76.0%).

^{1}H NMR (250 MHz, $CDCl_3$) δ: 7.92 (s, 2H, $H^{5,10}$), 7.33 (d, J = 1.9 Hz, 1H, H^{15}), 7.31 – 7.26 (m, 2H, $H^{14,16}$), 7.25 – 7.20 (m, 2H, $H^{13,17}$), 6.70 (dt, J = 4.2, 2.1 Hz, 2H, $H^{1,9}$), 6.16 (dd, J = 5.9, 2.8 Hz, 2H, $H^{3,7}$), 5.97 – 5.88 (m, 2H, $H^{2,8}$), 5.48 (s, 1H, H^{11}).

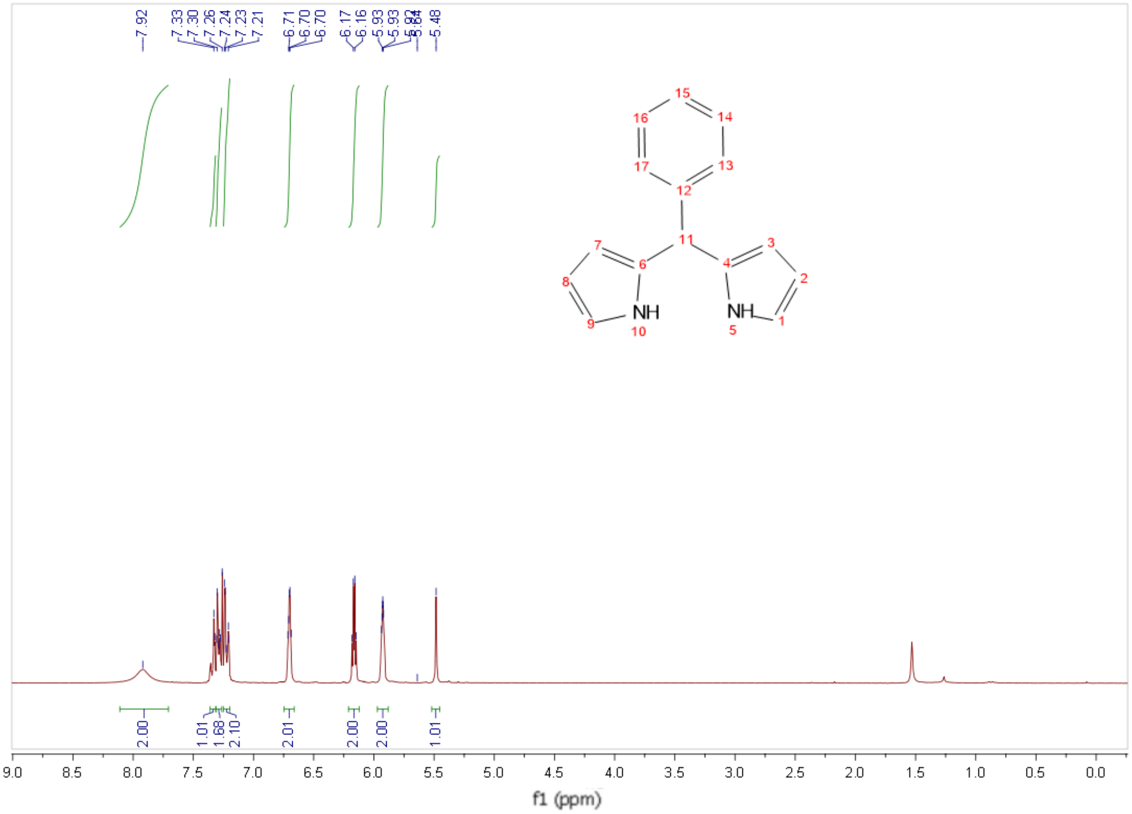

^{1}H NMR spectrum of dipyrroliphenylmethane

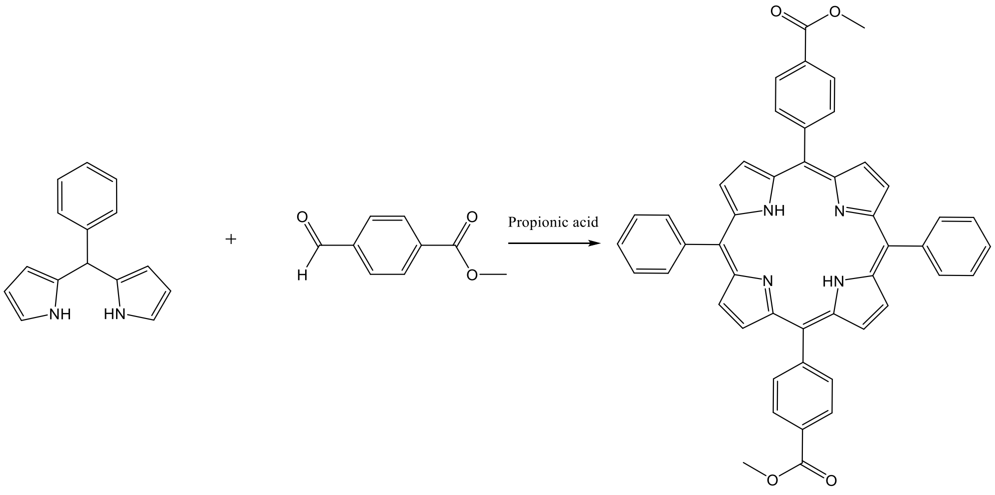
7

Dipyrroliphenylmethane (2.0 g, 9 mmol) and methyl 4-formylbenzoate (1.47 g, 9 mmol) were dissolved in 40 mL of propionic acid, and the mixture was refluxed for 1 h. The solvent was

evaporated under reduced pressure. Purification was done by silica gel column chromatography with CH_2Cl_2 to afford the molecule **7** as a purple solid (470 mg, yield 7.1%).

^{1}H NMR (360 MHz, $CDCl_3$) δ: 8.88 (d, J = 4.9 Hz, 4H, $H^{2,3,9,10}$), 8.80 (d, J = 4.9 Hz, 4H, $H^{15,16,21,22}$), 8.45 (d, J = 8.2 Hz, 4H, $H^{27,29,31,35}$), 8.31 (d, J = 8.1 Hz, 4H, $H^{26,30,32,34}$), 8.25 – 8.18 (m, 4H, $H^{40,42,45,47}$), 7.84 – 7.71 (m, 6H, $H^{39,41,43,44,46,48}$), 4.12 (s, 6H, $H^{51,55}$), -2.79 (s, 2H, $H^{12,24}$).

^{13}C NMR (91 MHz, $CDCl_3$) δ: 167.33 (s, $C^{49,52}$), 146.93 (d, J = 2.2 Hz, $C^{1,11,14,23}$), 146.91 (d, J = 2.2 Hz, $C^{4,8,17,20}$), 141.93 (d, J = 2.2 Hz, $C^{7,19}$), 141.91 (d, J = 2.2 Hz, $C^{28,36}$), 134.58 (d, J = 3.1 Hz, $C^{37,38}$), 134.54 (d, J = 3.1 Hz, $C^{25,33}$), 129.63 (s, $C^{26,30,32,34}$), 127.95 (d, J = 6.4 Hz, $C^{27,29,31,35}$), 127.88 (d, J = 6.4 Hz, $C^{39,43,44,48}$), 126.78 (s, $C^{41,46}$), 120.81 (d, J = 17.7 Hz, $C^{40,42,45,47}$), 120.62 (d, J = 17.7 Hz, $C^{15,22,2,10}$), 118.96 (d, J = 16.8 Hz, $C^{3,9,16,21}$), 118.77 (d, J = 16.8 Hz, $C^{6,13}$), 52.47 (s, $C^{51,55}$).

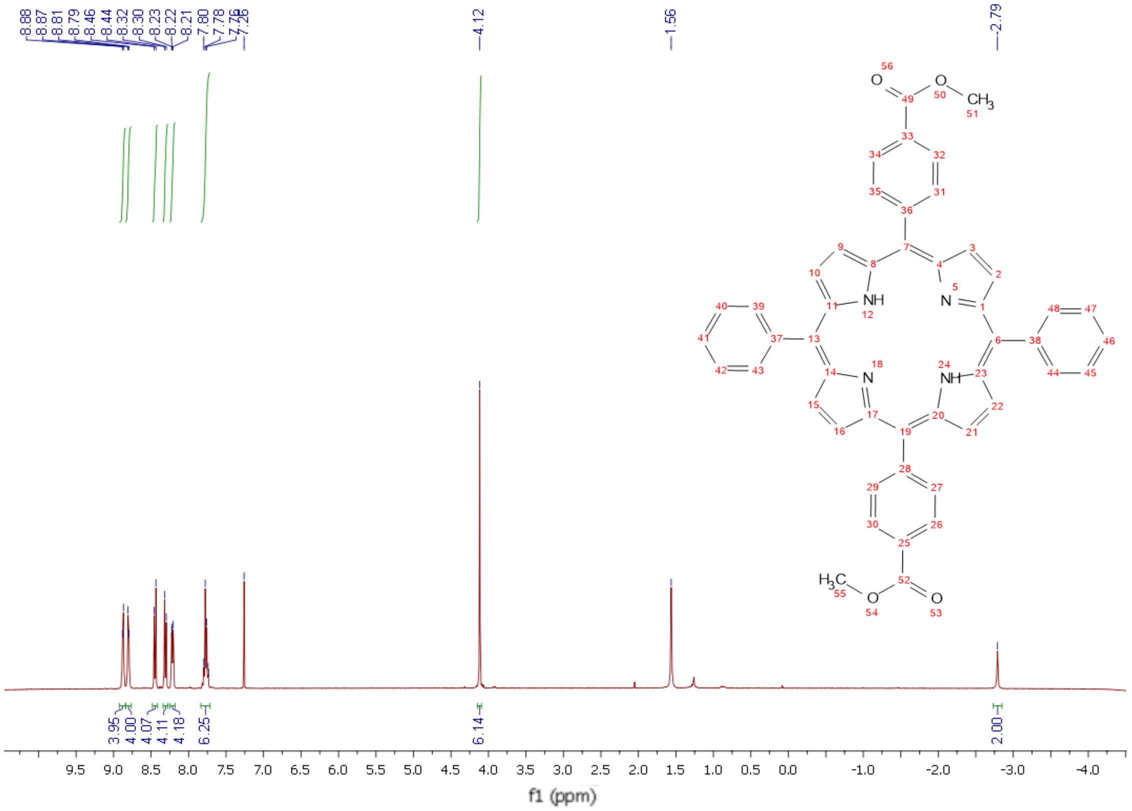

^{1}H NMR spectrum of **7**

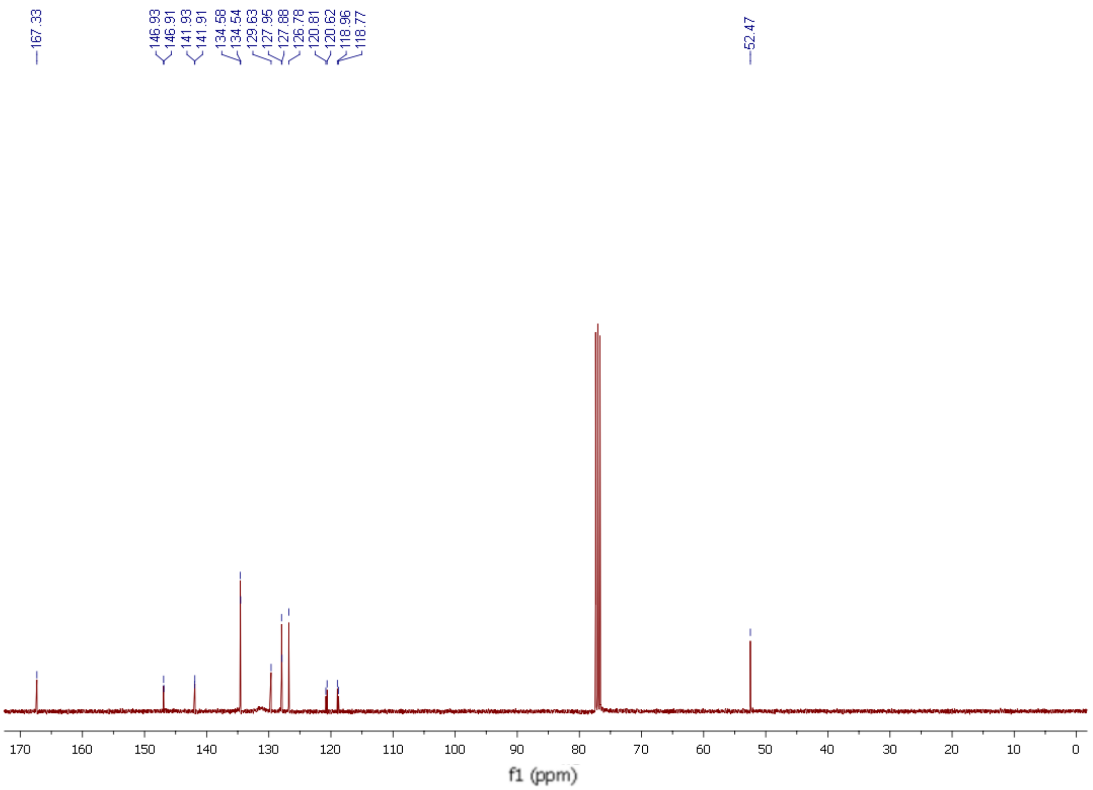

^{13}C NMR spectrum of **7**

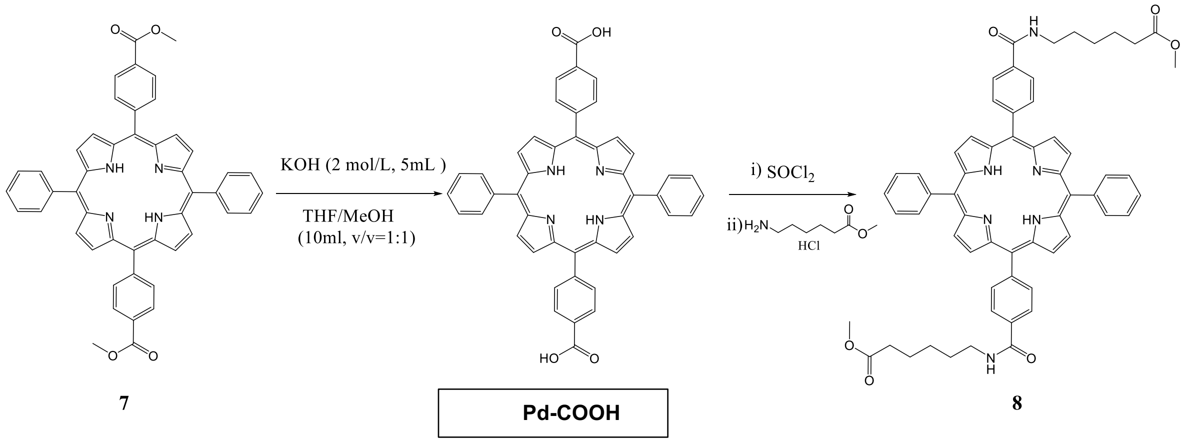

Compound **7** (40 mg, 0.055mmol) was dissolved in 5 mL THF and 5 mL MeOH. KOH solution (2 M, 5 mL) was added to the previous solution refluxing for 6 h. After cooling to room temperature, the mixture was acidified with HCl (2 M) to pH 2. The compound was collected by filtration to afford a dark-green solid (28 mg, yield 72.8%). **Pd-COOH** (450mg, 0.64mmol) was placed in 100 mL 2-necked flask and 50mL of benzene were added under N_2 atmosphere. Then 10 mL of thionyl chloride was added to the suspension and refluxed for 5 h with catalytic quantity of dehydrated DMF. After removal of the solvent and redundant thionyl chloride under the reduced pressure, the

residue was dissolved in 20 mL dehydrate CH_2Cl_2 and then added to the mixture of methyl 6-aminohexanoate hydrochloride (581 mg, 3.2 mmol) and triethylamine (323mg, 3.2mmol) which were previously dissolved in 20 mL CH_2Cl_2. After removal of the solvents under reduced pressure, the resulting mixture was purified by silica gel column chromatography with CH_2Cl_2 / MeOH, 100:1 v/v as eluent mixture, to afford the molecule **8** as a purple solid (436 mg, 71.1%).

^{1}H NMR (360 MHz, $CDCl_3$) δ: 8.83 (dd, J = 24.8, 4.6 Hz, 8H, $H^{2,3,9,10,15,16,21,22}$), 8.26 (d, J = 7.9 Hz, 4H, $H^{27,29,31,35}$), 8.19 (d, J = 6.8 Hz, 4H, $H^{26,30,32,34}$), 8.13 (d, J = 7.9 Hz, 4H, $H^{40,42,45,47}$), 7.85 – 7.68 (m, 6H, $H^{39,41,43,44,46,48}$), 6.58 (t, J = 5.6 Hz, 2H, $H^{50,53}$), 3.71 (s, 6H, $H^{71,72}$), 3.64 (dd, J = 13.0, 6.6 Hz, 4H, $H^{55,63}$), 2.42 (t, J = 7.3 Hz, 4H, $H^{59,67}$), 1.82 – 1.73 (m, 8H, $H^{56,58,64,66}$), 1.56 (dd, J = 15.2, 8.1 Hz, 4H, $H^{57,65}$), -2.79 (s, 2H, $H^{12,24}$).

^{13}C NMR (91 MHz, $CDCl_3$) δ: 174.21 (s, $C^{60,68}$), 167.63 (s, $C^{49,51}$), 145.32 (s, $C^{1,11,14,23,4,8,17,20}$), 141.89 ($s^{7,19,28,36}$), 134.57 (d, J = 12.5 Hz, $C^{37,38}$), 134.12 (s, $C^{25,33}$), 127.86 (s, $C^{26,30,32,34}$), 126.76 (s, $C^{41,46}$), 125.31 (s, $C^{40,42,45,47}$), 120.55 (s $C^{15,22,2,10}$), 118.98 (s, $C^{3,9,16,21,6,13}$), 51.63 (s, $C^{71,72}$), 39.99 (s, $C^{55,63}$), 33.91 (s, $C^{59,67}$), 29.42 (s, $C^{56,64}$), 26.51 (s, $C^{57,65}$), 24.49 (s, $C^{58,66}$).

MS-ESI (*m/z*): $[M]^+$ Calcd. for $C_{60}H_{56}N_6O_6^+$ 957.4, found 957.4.

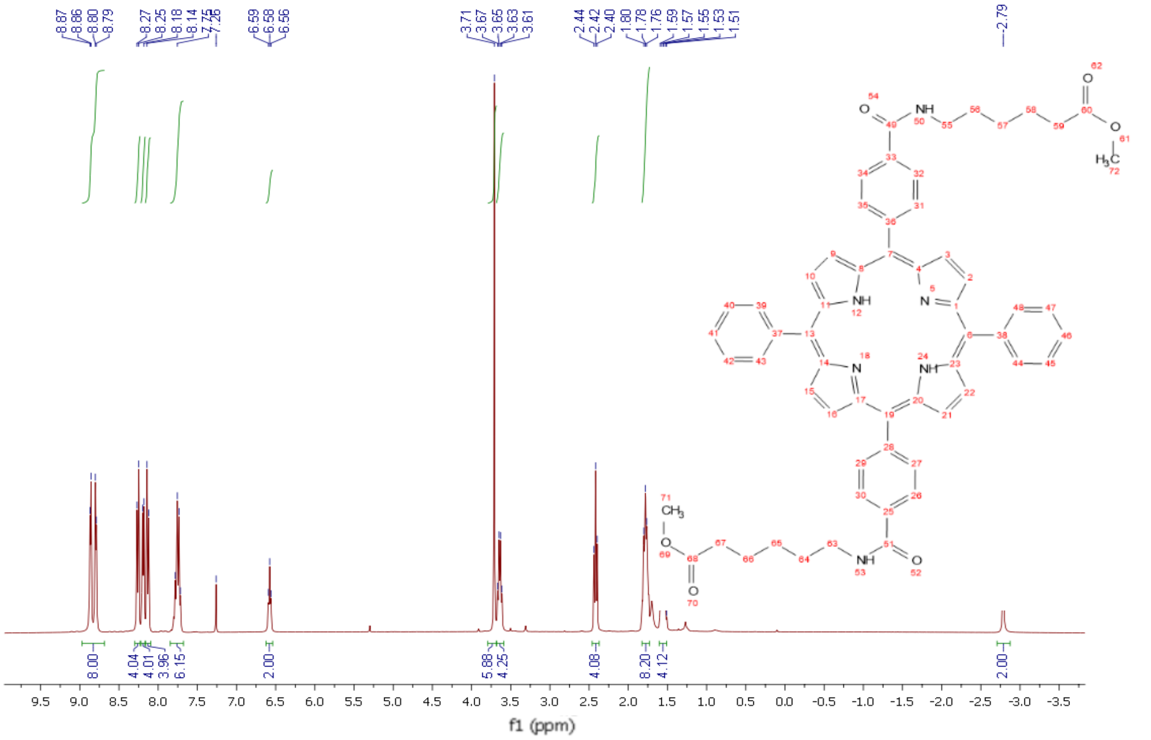

^{1}H NMR spectrum of **8**

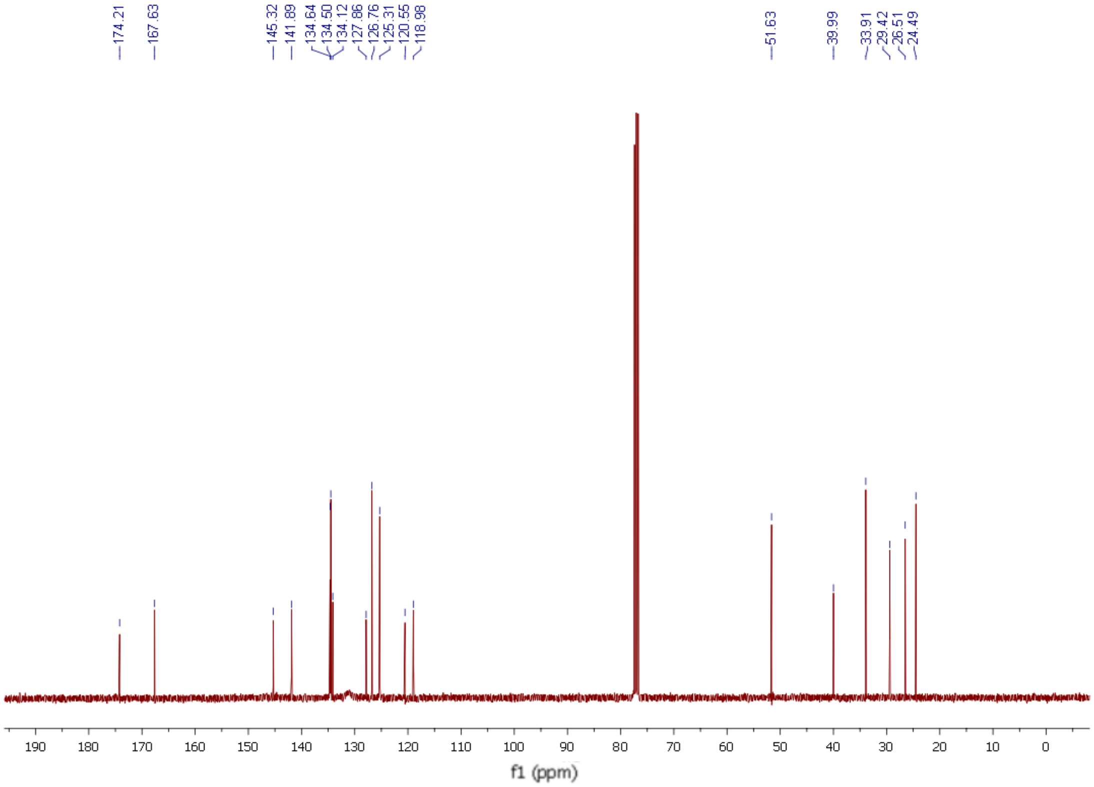

^{13}C NMR spectrum of **8**

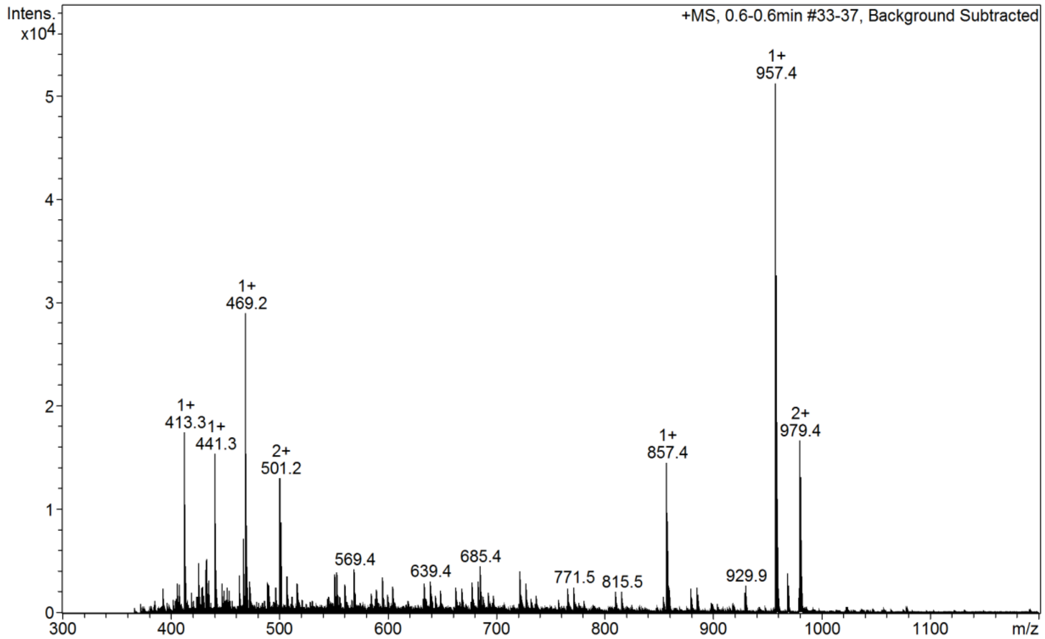

Mass spectrometry of **8**

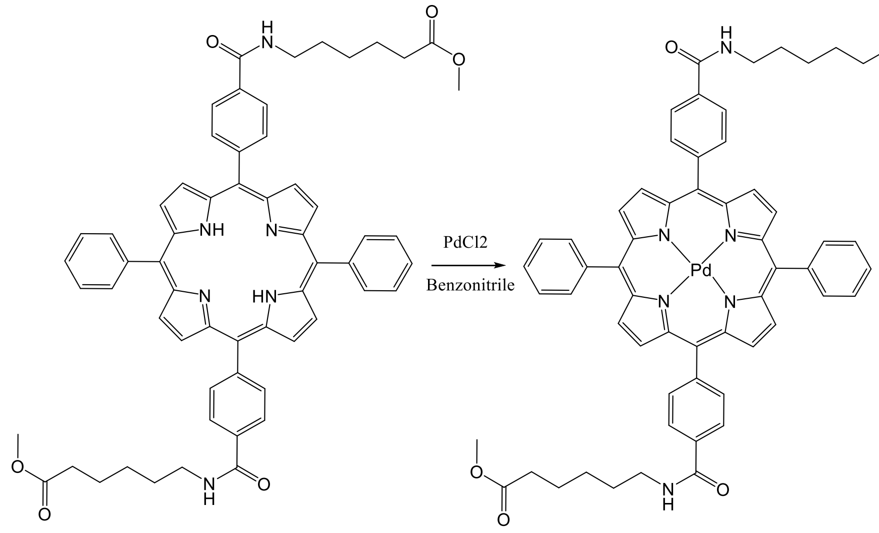

8 **9**

Compound **8** (70 mg, 0.073 mmol) and $PdCl_2$ (16 mg, 0.087 mmol) were dissolved in benzonitrile (30 mL) and refluxed for 4.5 h. After removal of the solvents under reduced pressure, the residue was purified by column chromatography on a silica gel using CH_2Cl_2 / MeOH, 100:1 v/v as eluent mixture, to obtain the molecule **9** (56 mg, 0.064 mmol, 72.2%) as an orange solid.

^1H NMR (360 MHz, $CDCl_3$) δ: 8.81 (d, J = 5.0 Hz, 4H, $H^{2,3,9,10,'}$), 8.74 (d, J = 5.0 Hz, 4H, $H^{15,16,21,22}$), 8.21 (d, J = 8.1 Hz, 4H, $H^{26,30,32,34}$), 8.11 (t, J = 9.1 Hz, 8H, $H^{26,30,32,34,40,42,45,47}$), 7.74 (dd, J = 11.4, 7.2 Hz, 6H, $H^{39,41,43,44,46,48}$), 6.52 (t, J = 5.7 Hz, 2H, $H^{50,53}$), 3.70 (s, 6H, $H^{71,72}$), 3.62 (dd, J = 13.0, 6.6 Hz, 4H, $H^{55,63}$), 2.41 (t, J = 7.3 Hz, 4H, $H^{59,67}$), 1.76 (dd, J = 14.9, 7.4 Hz, 8H, $H^{56,58,64,66}$), 1.55 (dd, J = 15.2, 8.3 Hz, 4H, $H^{57,65}$).

^{13}C NMR (91 MHz, $CDCl_3$) δ: 174.19 (s, $C^{60,68}$), 167.58 (s, $C^{49,51}$), 144.91 (s, $C^{1,11,14,23}$) 141.69 (s, $C^{4,8,17,20}$), 141.49 (s, $C^{28,36}$), 141.15 (s, $C^{37,38}$) 134.20 (s, $C^{15,22,2,10}$), 134.04 (s, $C^{26,30,32,34}$), 131.40 (s, $C^{41,46}$), 130.73 (s, $C^{40,42,45,47}$) 129.09 (s, $C^{25,33}$), 127.90 (s, $C^{3,9,16,21}$), 126.79 (s, $C^{29,27,31,35}$), 125.33 (s, $C^{3,9,16,21}$), 122.11 (s, $C^{7,19}$), 120.62 (s, $C^{6,13}$), 51.62 (s, $C^{71,72}$), 39.96 (s, $C^{55,63}$), 33.90 (s, $C^{59,67}$), 29.41 (s, $C^{56,64}$), 26.49 (s, $C^{57,65}$), 24.47 (s, $C^{58,66}$).

DEPT135 (91 MHz, $CDCl_3$) δ134.19 (s, $C^{15,22,2,10}$), 134.04 (s, $C^{26,30,32,34}$), 131.40 (s, $C^{41,46}$), 130.73 (s, $C^{40,42,45,47}$) 127.90 (s, $C^{3,9,16,21}$), 126.79 (s, $C^{29,27,31,35}$), 125.33 (s, $C^{3,9,16,21}$), 51.62 (s, $C^{71,72}$), 39.96 (s, $C^{55,63}$), 33.90 (s, $C^{59,67}$), 29.41 (s, $C^{56,64}$), 26.49 (s, $C^{57,65}$), 24.47 (s, $C^{58,66}$).

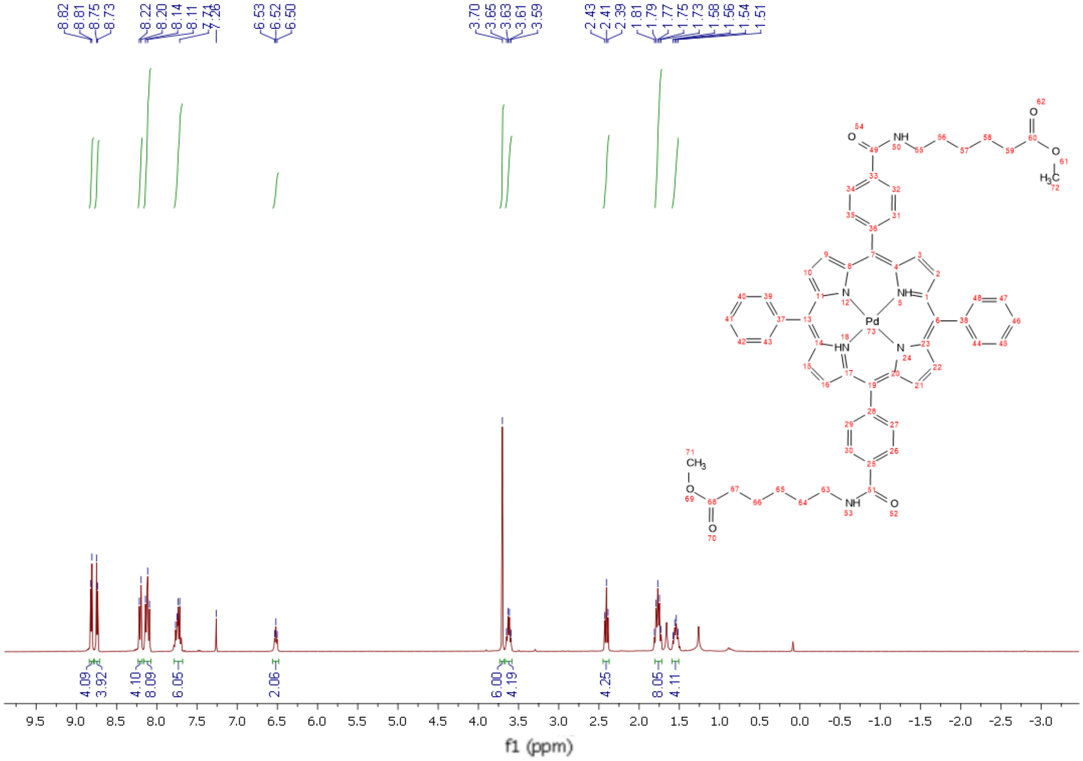

^{1}H NMR spectrum of **9**

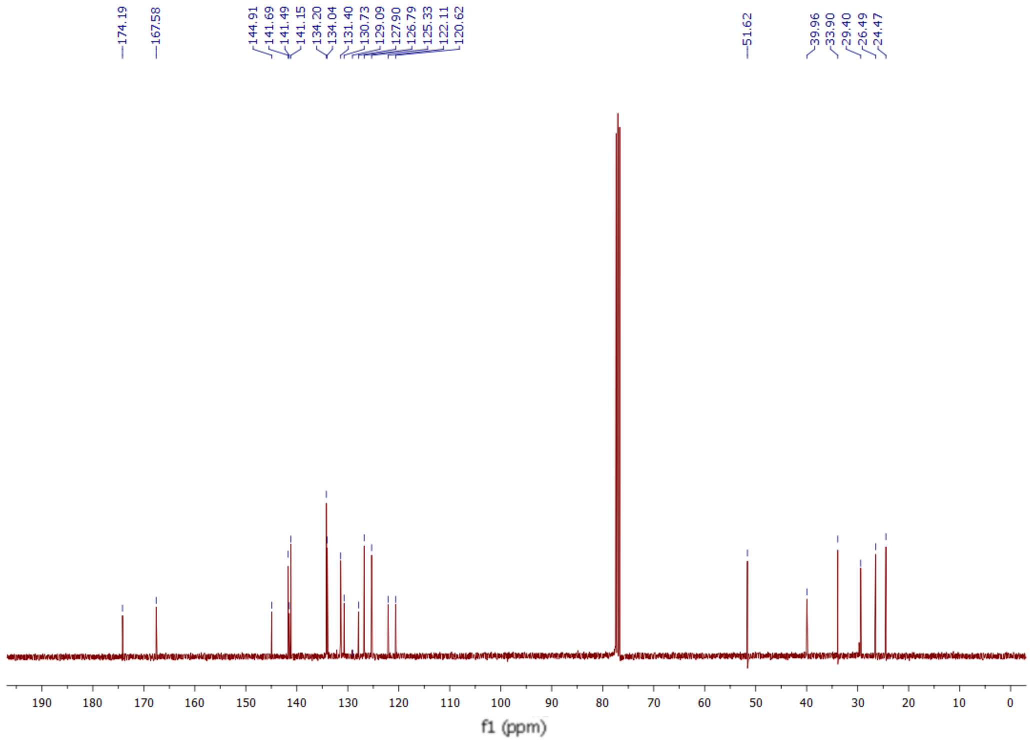

^{13}C NMR spectrum of **9**

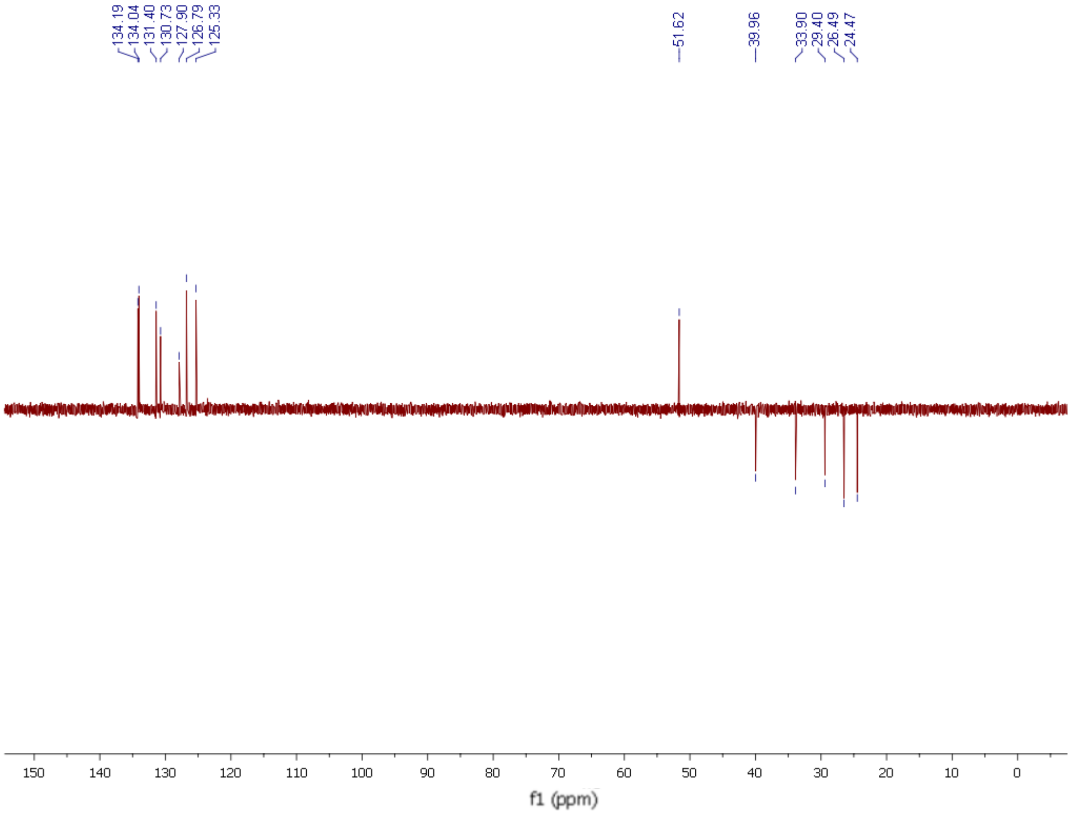

DEPT135 spectrum of **9**

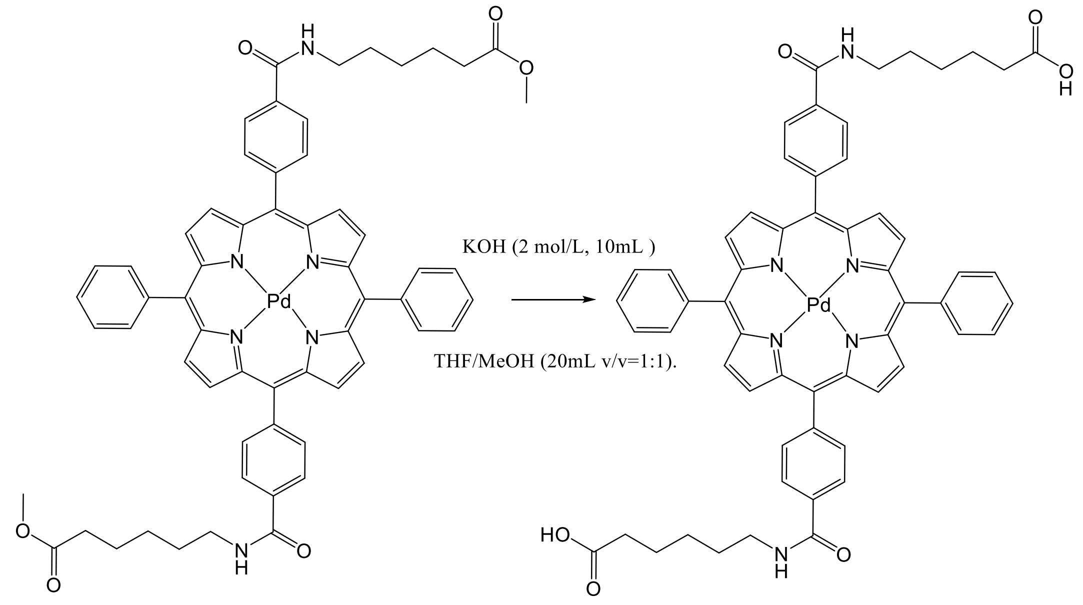

9 **10 (Pd-S-COOH)**

Compound **9** (56 mg, 0.053mmol) was dissolved in 10 mL of THF and 10mL of MeOH. KOH solution (2 mol/L, 10mL) was added to the previous solution refluxing for 5 h. After cooling to room temperature, the mixture was acidified with HCl (1 mol/L) to pH 7. The compound was collected by filtration to afford the molecule **10** as an orange solid (50 mg, yield 91.7%).

^{1}H NMR (360 MHz, $(CD_3)_2SO$) δ: 12.10 (s, 2H, $H^{61,69}$), 8.89 (s, 2H, $H^{50,53}$), 8.81 (s, 7H, Ar-H), 8.34 – 8.15 (m, 11H, Ar-H), 7.83 (t, J = 6.9 Hz, 6H, $H^{39,41,43,44,46,48}$), 3.42 (d, J = 6.2 Hz, 4H, $H^{55,63}$), 2.29 (t, J = 7.3 Hz, 4H, $H^{59,67}$), 1.73 – 1.56 (m, 8H, $H^{56,58,64,66}$), 1.51 – 1.38 (m, 4H, $H^{57,65}$).

^{13}C NMR (91 MHz, $(CD_3)_2SO$) δ: 175.00 (s, $C^{60,68}$), 166.47 (s, $C^{49,51}$), 143.76 (s, $C^{1,11,14,23}$) 141.38 (s, $C^{4,8,17,20}$), 141.11 (s, $C^{28,36}$), 141.03 (s, $C^{37,38}$) 134.80 (s, $C^{25,33}$), 134.25 (s, $C^{26,30,32,34}$), 131.81 (s, $C^{41,46}$), 131.94 (s, $C^{40,42,45,47}$) 131.69 (s, $C^{15,22,2,10}$), 128.70 (s, $C^{3,9,16,21}$), 127.55 (s, $C^{29,27,31,35}$), 126.34 (s, $C^{3,9,16,21}$), 122.38 (s, $C^{7,19}$),121.52 (s, $C^{6,13}$), 39.95 (s, $C^{55,63}$), 34.16 (s, $C^{59,67}$), 29.46 (s, $C^{56,64}$), 26.62 (s, $C^{57,65}$), 24.82 (s, $C^{58,66}$).

MS-ESI (*m/z*): $[M]^+$ Calc. for $C_{58}H_{50}N_6O_6Pd^-$ 1031.3, found 1031.3

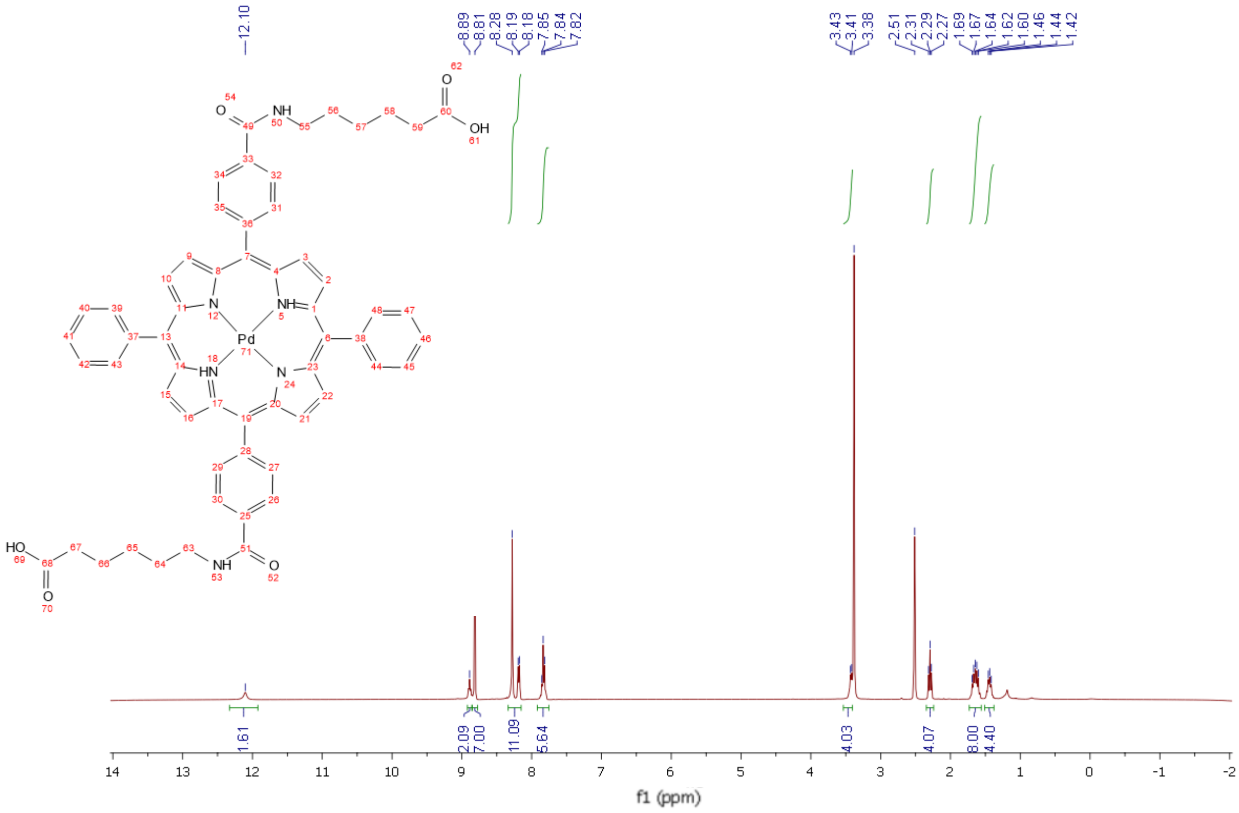

^{1}H NMR spectrum of **10** (**Pd-S-COOH**)

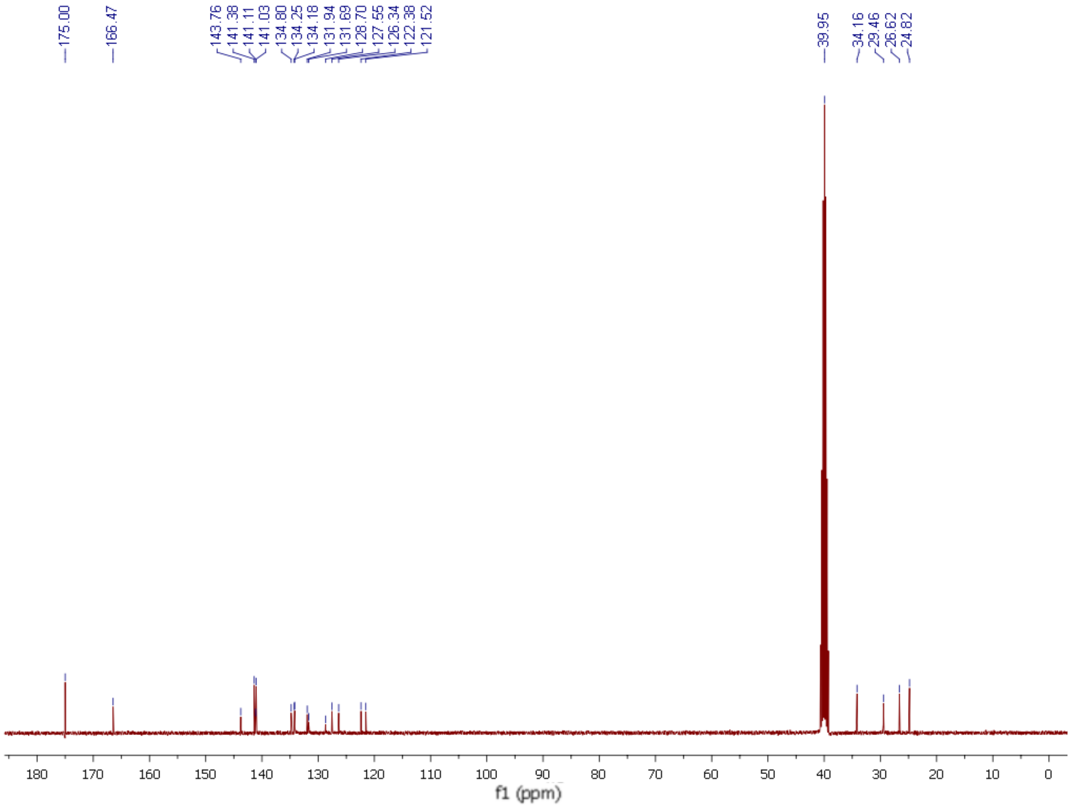

^{13}C NMR spectrum of **10** (**Pd-S-COOH**)

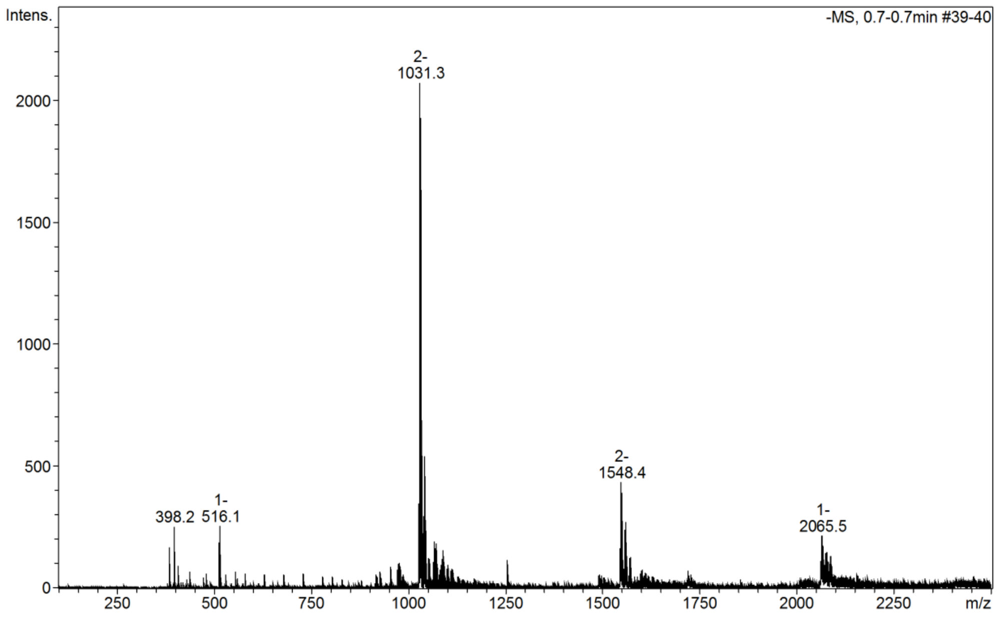

Mass spectrometry of **10** (**Pd-S-COOH**)

Section 3. Synthesis and characterization data of CAEBD-S-COOH and intermediates to obtain it

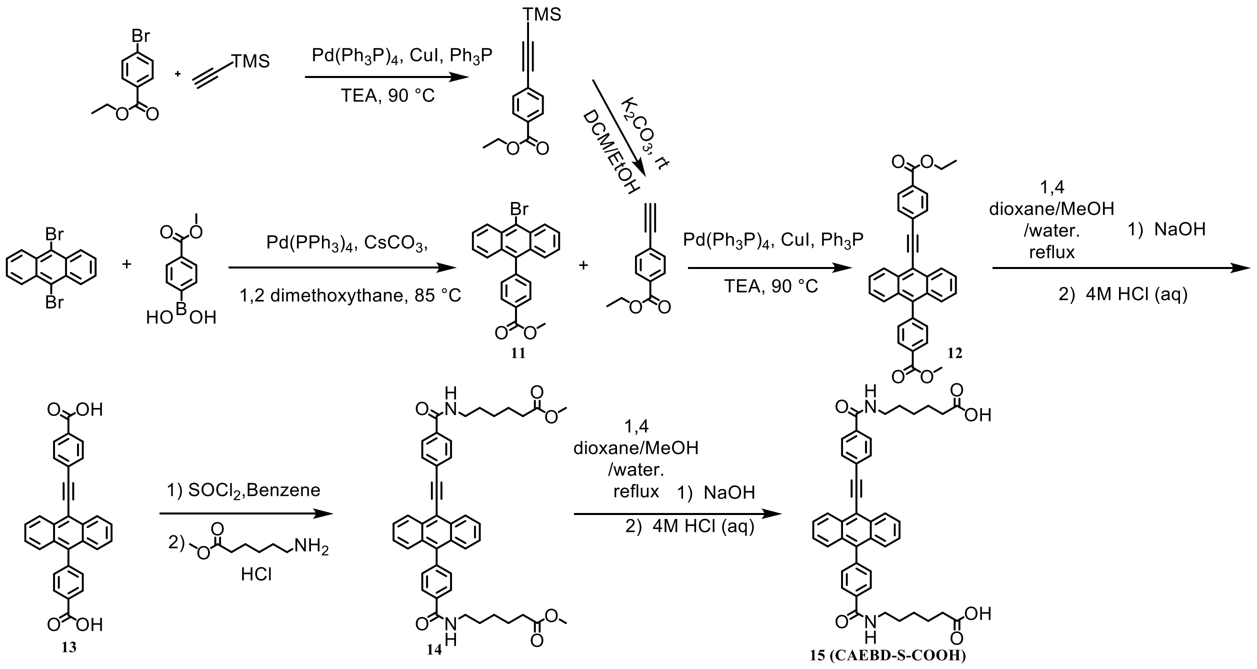

Synthetic route of **15 (CAEBD-S-COOH)**

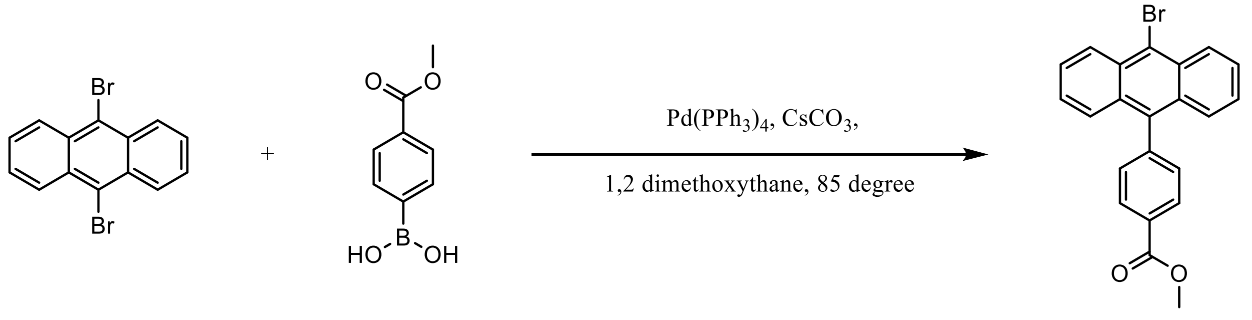

11

The mixture of 9,10-dibromoanthracene (4.0 g, 11.90 mmol), (4 - (methoxycarbonyl) phenyl) boronic acid (0.85 g, 4.76 mmol), $CsCO_3$ (4.0 g, 11.90 mmol)and a catalytic amount of $Pd(PPh_3)_4$ (192 mg, 0.016 mmol) were dissolved in 250 mL 1,2 CH_2Cl_2. The reaction was heated to 85 ℃ for 22 h under N_2 atmosphere. The crude product was purified by column chromatography using $CHCl_3$:hexane=8:2 to afford the molecule **11** as a yellowish solid after solvent evaporation (1.5 g, 45.7 %).

^{1}H NMR (400 MHz, $CDCl_3$) δ: 8.62 (d, J = 8.9, 0.7 Hz, 2H, $H^{3,11}$), 8.36 – 8.23 (m, 2H, $H^{6,14}$), 7.66 – 7.55 (m, 4H, $H^{16,17,19,20}$), 7.54 – 7.46 (m, 2H, $H^{2,12}$), 7.38 (m, J = 8.8, 6.5, 1.1 Hz, 2H, $H^{1,13}$), 4.02 (s, J = 1.8 Hz, 3H, H^{25}).

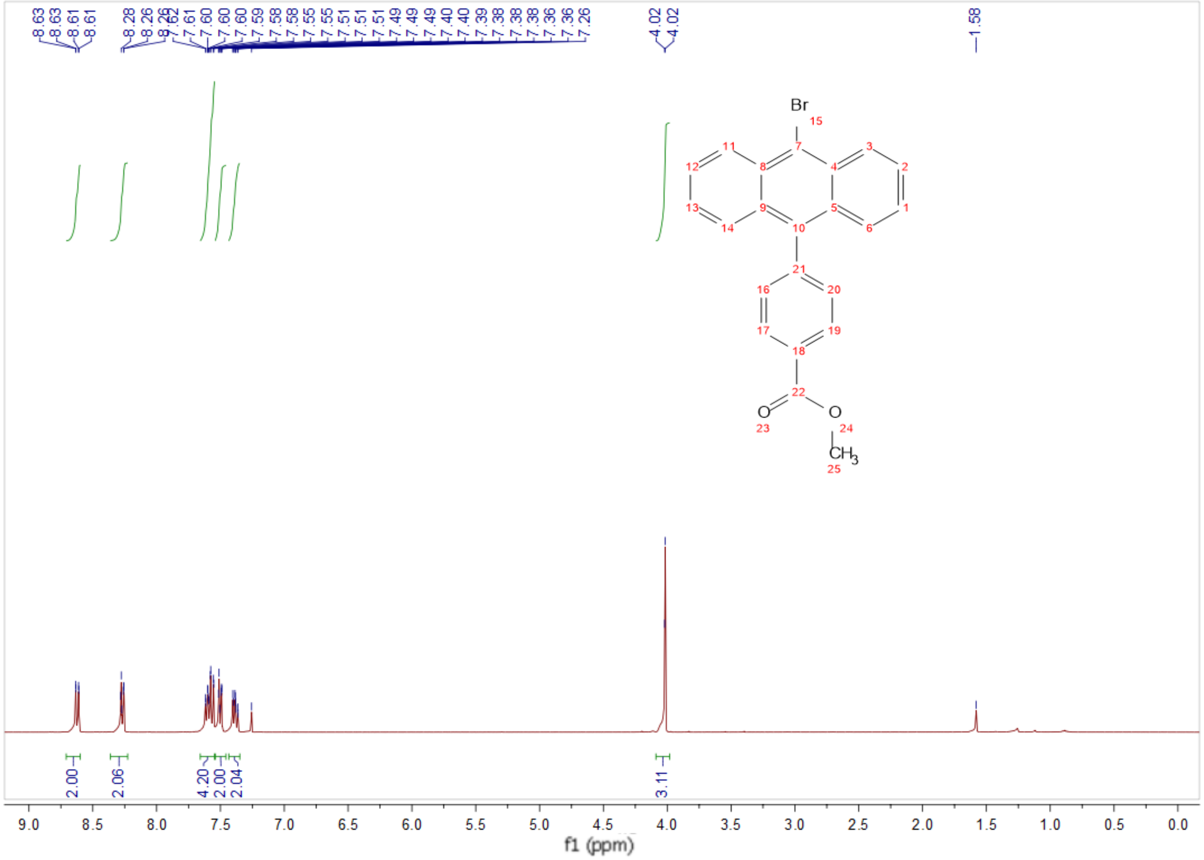

^{1}H NMR spectrum of **11**

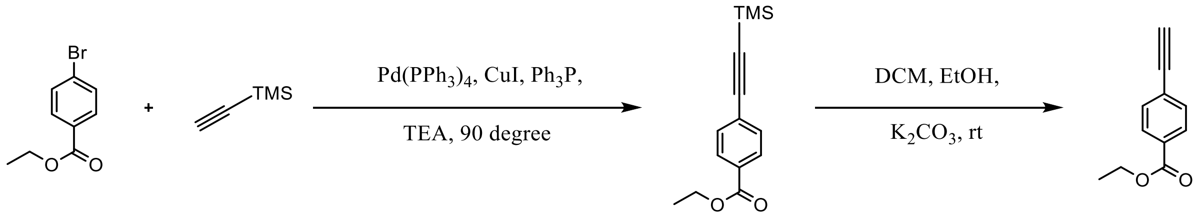

A mixture of ethyl 4-bromobenzoate (2.01 g, 8.77 mmol, 1.0 equiv.), Pd(PPh3)4 (504 mg, 0.44 mmol, 0.05 equiv.), CuI (83 mg, 0.44 mmol, 0.05 equiv.) and PPh_3 (137 mg, 0.53 mmol, 0.06 equiv.) was dissolved in triethylamine (60 ml) under argon atmosphere. Ethynyltrimethylsilane (1.03 g 10.49 mmol, 1.2 equiv.) was added and the reaction mixture was heated at 90 °C for 17

h. After cooling to room temperature, the mixture was filtered through CELITE and evaporated to dryness.

In a second step, to a solution of K_2CO_3 (2.33 g, 16.8 mmol, 2.0 equiv.) in EtOH/CH_2Cl_2 (50 mL/10 mL) a mixture of the obtained ethyl-4-((trimethylsilyl)ethynyl)benzoate (2.08 g, 8.4 mmol, 1.0 equiv.) was added under argon atmosphere. The reaction mixture was stirred for 4 h at room temperature, then filtered and evaporated to dryness. The crude was purified by flash chromatography to afford 1.27 g (86.7%). R_f = 0.40 (hexanes: CH_2Cl_2= 1:1).

^{1}H NMR (360 MHz, $CDCl_3$) δ: 8.02 – 7.95 (m, 2H, $H^{2,4}$), 7.54 (d, J=8.3, 2H, $H^{1,5}$), 4.37 (q, J=7.1, 2H, H^{11}), 3.22 (s, 1H, H^{12}), 1.39 (t, J=7.1, 3H, H^{13}).

^{13}C NMR (91 MHz, $CDCl_3$) δ: 165.93 (s, C^8), 132.02 (s, $C^{1,5}$), 130.48 (s, C^3), 129.41 (s, $C^{2,4}$), 126.61 (s, C^6), 82.84 (s, C^7), 79.97 (s, C^{12}), 61.20 (s, C^{77}), 14.29 (s, C^{13})

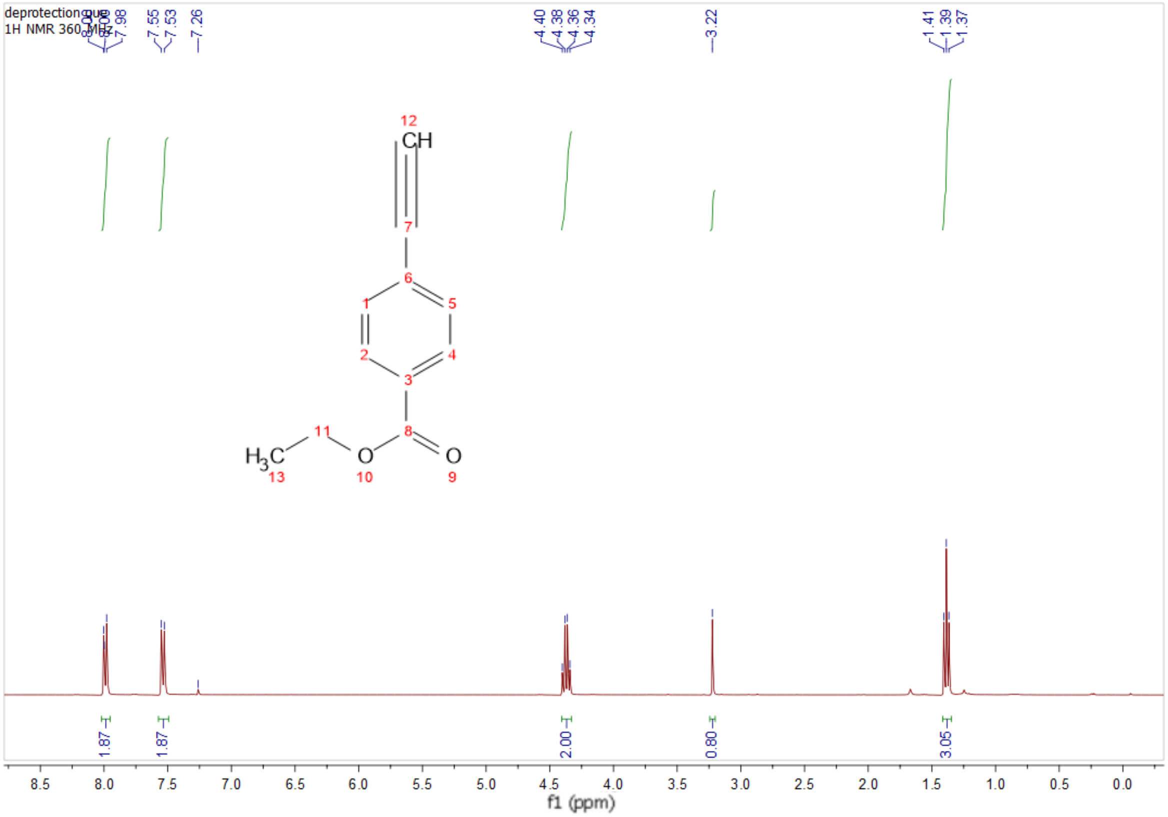

^{1}H NMR spectrum of ethyl 4-ethynylbenzoate

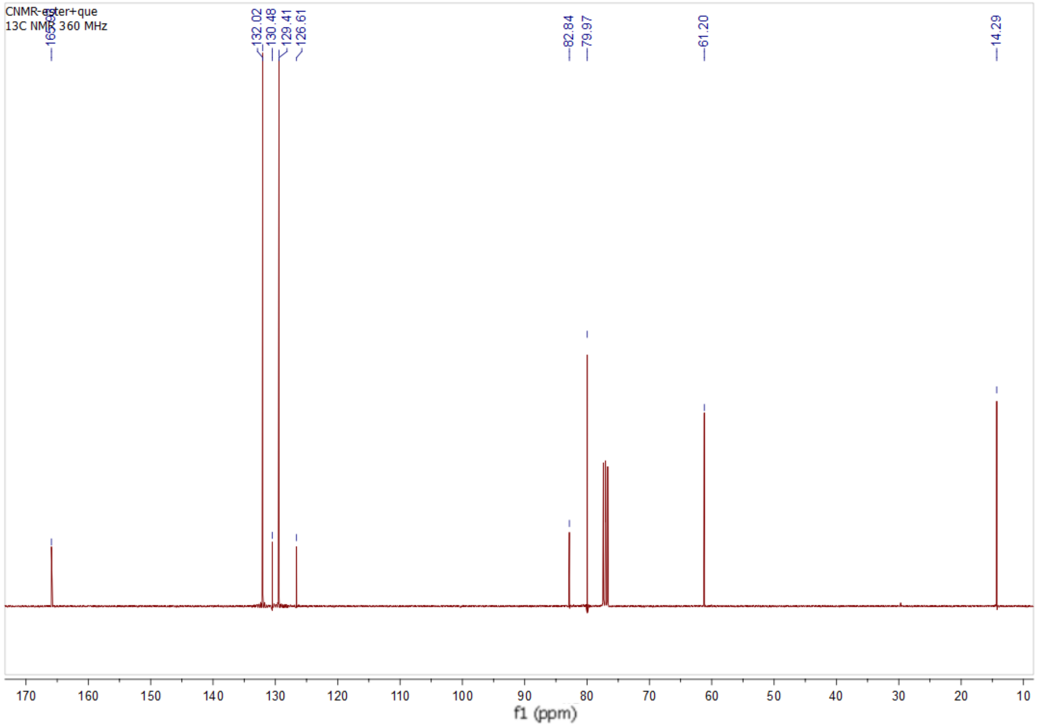

^{13}C NMR spectrum of ethyl 4-ethynylbenzoate

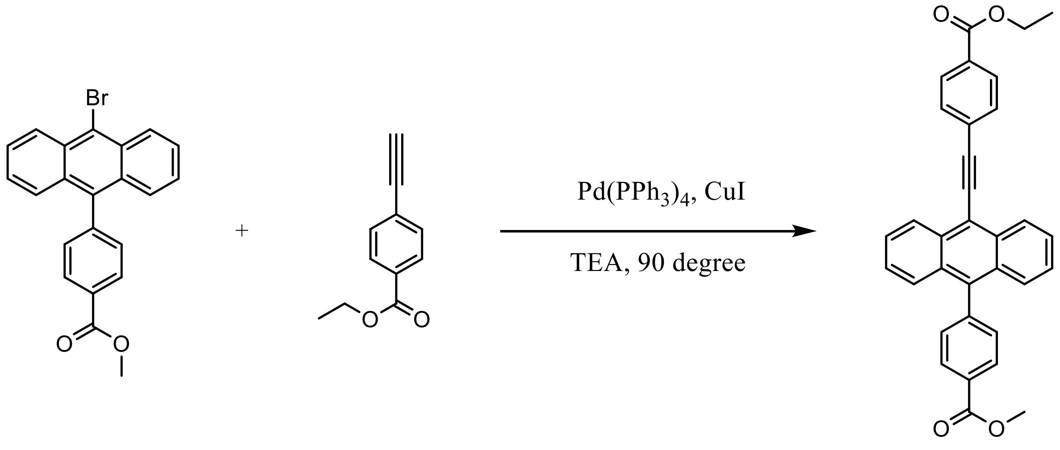

12

11 (1.5 g, 3.84 mmol), ethyl 4-ethynylbenzoate (0.855 g, 5.34 mmol), $Pd(PPh_3)_4$ (250 mg, 0.22 mmol) and CuI (55 mg, 0.28 mmol) were mixed in 60 mL THF and 60 mL di-isopropyl amine under N_2 atmosphere. The reaction was refluxed for 24h. The solvent was removed, and the reaction mixture was washed with H_2O and $CHCl_3$. The organic phase was dried, evaporated under vaccum and finally purified by column chromatography (hexane: CH_2Cl_2 = 2:1) to afford the

molecule **12** as a pale yellow solid after solvent evaporation. The solid was recrystallized from hot acetone. Yield: 1.3 g (73%).

^{1}H NMR (400 MHz, $CDCl_3$) δ: 8.73 (d, J = 8.7 Hz, 2H, $H^{4,14}$), 8.28 (d, J = 8.1 Hz, 2H, $H^{3,11}$), 8.14 (d, J = 8.3 Hz, 2H, $H^{16,18}$), 7.85 (d, J = 8.1 Hz, 2H, $H^{25,27}$), 7.62 (dd, J = 12.4, 5.6 Hz, 4H, $H^{15,19,24,28}$), 7.53 (d, J = 8.1 Hz, 2H, $H^{2,12}$), 7.42 (dd, J = 8.1, 7.1 Hz, 2H, $H^{1,13}$), 4.44 (q, J = 7.1 Hz, 2H, H^{34}), 4.02 (s, 3H, H^{37}), 1.45 (t, J = 7.1 Hz, 3H, H^{36}).

^{13}C NMR (101 MHz, $CDCl_3$) δ: 167.12 (s, C^{31}), 165.98 (s, C^{21}), 143.61, 137.82, 131.49 (s, $C^{24.28}$), 131.31 (s, $C^{16,18}$), 129.70, 129.41, 126.69 (s, $C^{25,27}$), 117.63, 100.64 (s, C^{35}), 89.43 (s, C^{30}), 61.50 (s, C^{34}), 52.36 (s, C^{37}), 14.22 (s, C^{36}).

DEPT135 (101 MHz, $CDCl_3$) δ: 131.49 (s, $C^{24.28}$), 131.31 (s, $C^{16,18}$), 129.69 (s, $C^{25,27}$), 127.00 (s, $C^{15,19}$), 126.85 (s, $C^{2,12}$), 126.69 (s, $C^{1,13}$), 126.04 (s, $C^{6.14}$), 109.33 (s, $C^{3,11}$), 61.24 (s, C^{34}), 52.32 (s, C^{37}), 14.39 (s, C^{36}).

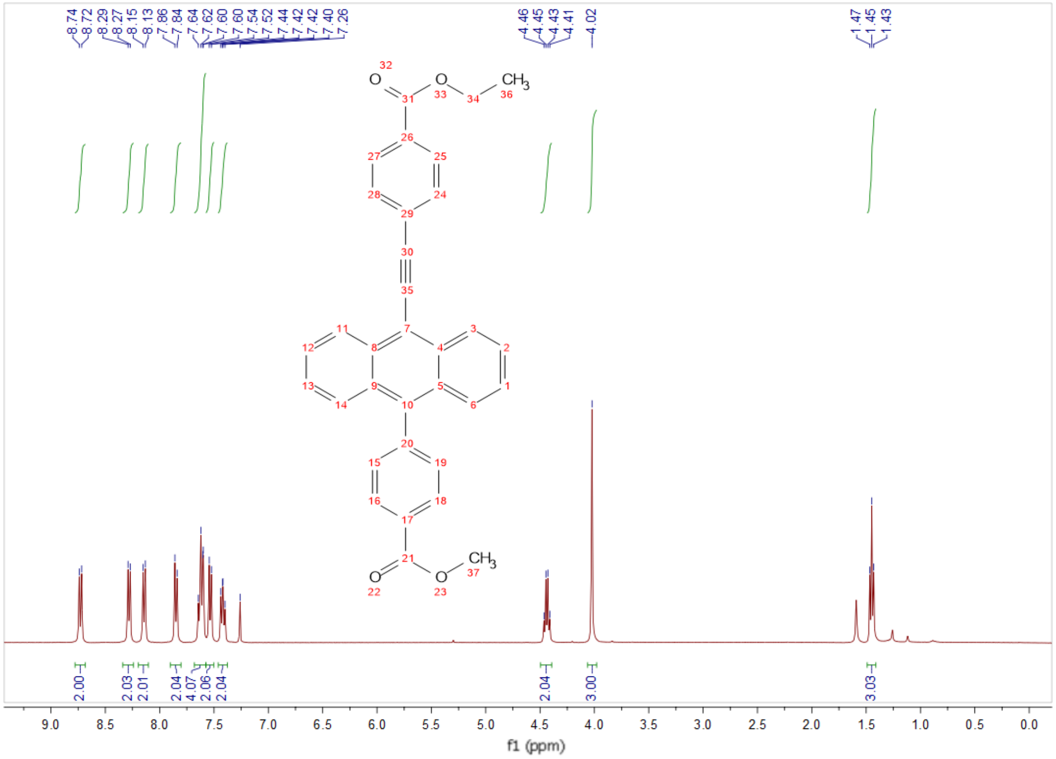

^{1}H NMR spectrum of **12**

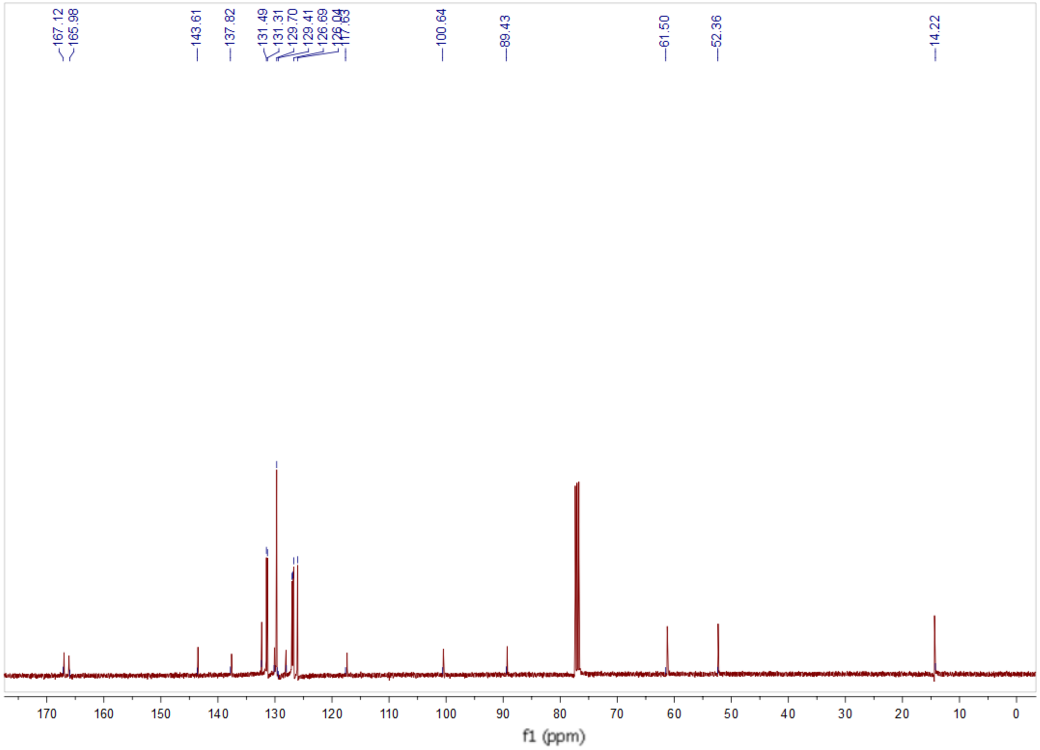

^{13}C NMR spectrum of **12**

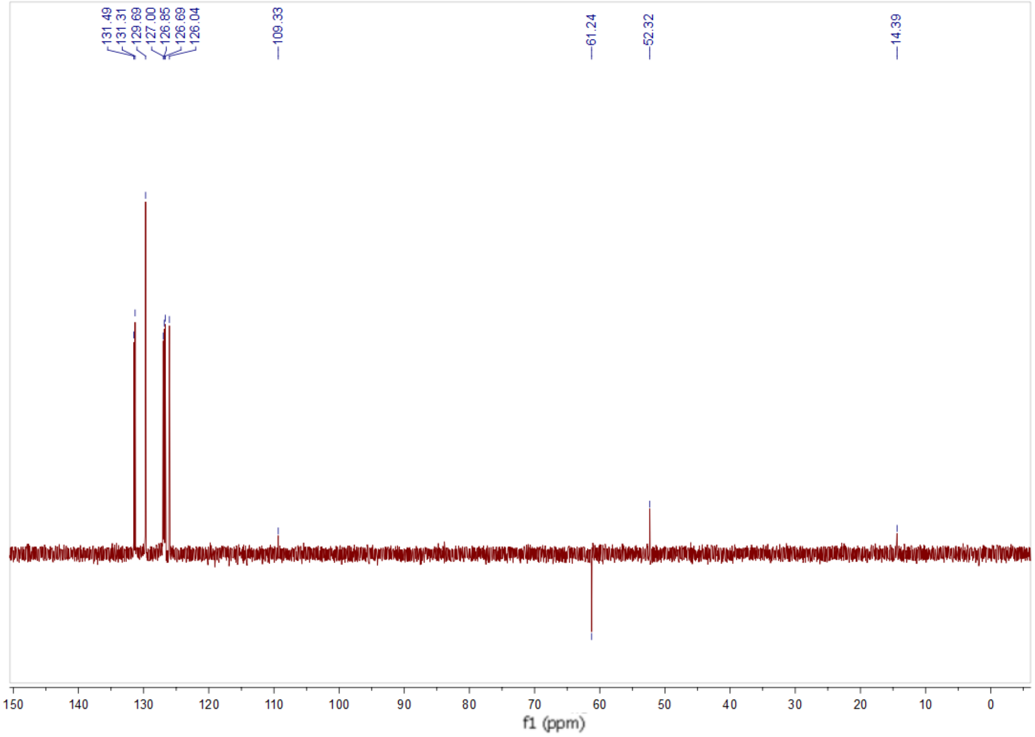

DEPT135 spectrum of **12**

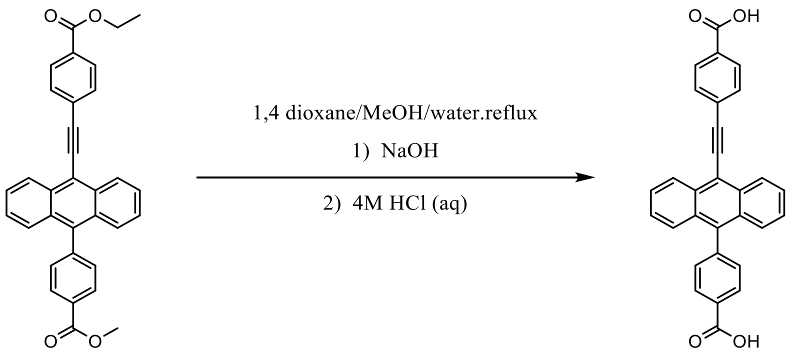

12 13

12 (1.0 g, 2.12 mmol) was suspended in 100 mL 1,4- dioxane/MeOH/water (v:v = 2:5:3) in a 250 mL flask. NaOH (8.0 mg, 200 mmol) was added and refluxed for 10 h at 60 ℃. The reaction was monitored by thin layer chromatography and when starting material was completed consumed, the pH was adjusted to pH 2 by adding HCl (2 M). Yellowish precipitate corresponding to molecule **13** was collected by filtration. Yield: 0.85 mg (91.0%).

^{1}H NMR (400 MHz, $(CD_3)_2SO$) δ: 13.21 (s, 2H, $H^{23,33}$), 8.70 (d, J = 8.6 Hz, 2H, $H^{6,14}$), 8.21 (d, J = 7.4 Hz, 2H, $H^{3,11}$), 8.07 (d, J = 7.5 Hz, 2H, $H^{16,18}$), 7.98 (d, J = 7.6 Hz, 2H, $H^{25,27}$), 7.73 (s, 2H, $H^{15,19}$), 7.55 (d, J = 12.7 Hz, 6H, $H^{1,2,12,13,24,28}$).

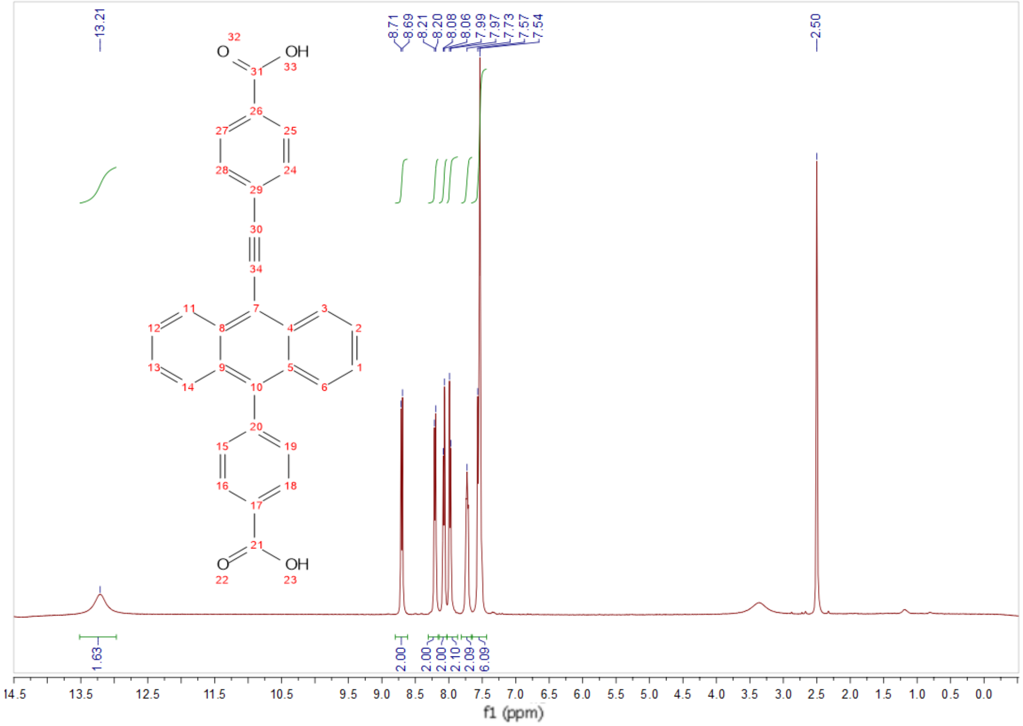

^{1}H NMR spectrum of **13**

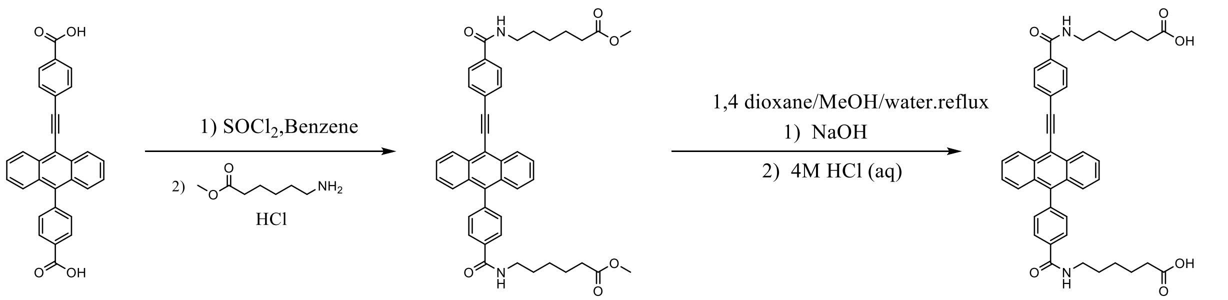

13 14 15 (CAEBD-S-COOH)

Compound **13** (70 mg, 0.167 mmol) was placed in 10 mL 2-necked flask and benzene (5 mL) was added under N_2 atmosphere. Then thionyl chloride was added to the suspension and refluxed for 5 h with catalytic amount of dehydrated DMF. After removing the redundant thionyl chloride and benzene, the residue was redispersed by anhydrous CH_2Cl_2 and used directly for the next step affording white solid **14** (85 mg, 75.9%). The **14** (85 mg, 0.13 mmol) was suspended in MeOH (15 mL), 1,4-dioxane (15ml) and KOH aqueous (2M, 15 mL). The mixture was refluxed at 60 ℃ for 20h. After cooling to room temperature, the mixture was adjusted to pH 2 using 4 M HCl. The precipitate was collected by filtration, achieving the molecule **15** as white solid after solvent evaporation (72.5 mg, 89.0%).

^{1}H NMR (400 MHz, *N, N*-dimethyl-formamide-d_7) δ: 12.21 (s, 2H, $H^{41,49}$), 8.84 (d, J = 8.7 Hz, 2H, $H^{6,14}$), 8.65 (dd, J = 25.4, 5.1 Hz, 2H, $H^{23,33}$), 8.28 (d, J = 8.2 Hz, 2H, $H^{3,11}$), 8.15 (d, J = 8.3 Hz, 2H, $H^{16,18}$), 8.06 (d, J = 8.3 Hz, 2H, $H^{25,27}$), 7.83 – 7.72 (m, 2H, $H^{15,19}$), 7.71 – 7.53 (m, 6H, $H^{1,2,12,13,24,28}$), 3.46 (d, J = 9.6 Hz, 4H, $H^{35,43}$), 2.42 – 2.21 (m, 4H, $H^{39,47}$), 1.80 – 1.58 (m, 8H, $H^{36,38,44,46}$), 1.55 – 1.41 (m, 4H, $H^{37,45}$).

^{13}C NMR (101 MHz, *N, N*-dimethyl-formamide-d_7) δ: 174.66 (s, C^{48}), 174.64 (s, C^{40}), 166.33 (s, C^{31}), 165.89 (s, C^{21}), 141.11 (s, C^{20}), 138.46 (s, C^{10}), 135.46 (s, C^{17}), 135.01 (s, C^{25}), 132.35 (s, $C^{4,8}$), 131.81 (s, $C^{5,915,19}$), 131.26 (s, $C^{24,28}$), 129.80 (s, $C^{5,9}$), 127.94 (s, $C^{2,12}$), 127.81 (s, $C^{16,18}$), 127.47 (s, $C^{25,27}$), 127.24 (s, $C^{1,13}$), 126.84 (s, $C^{6,14}$), 126.66 (s, $C^{3,11}$), 125.75 (s, C^{29}), 116.95 (s, C^{7}), 101.21 (s, C^{34}), 88.00 (s, C^{30}), 39.77 (s, C^{43}), 39.70 (s, C^{35}), 33.95 (s, C^{45}), 33.91 (s, C^{37}), 29.54 (s, C^{46}), 29.44 (s, C^{38}), 26.74 (s, C^{44}), 26.68 (s, C^{36}), 24.93 (s, C^{47}), 24.90 (s, C^{39}).

DEPT 135 (101 MHz, *N, N*-dimethyl-formamide-d_7) δ: 131.81 (s, $C^{15,19}$), 131.26 (s, $C^{24,28}$), 127.94 (s, $C^{2,12}$), 127.81 (s, $C^{16,18}$), 127.47 (s, $C^{25,27}$), 127.24 (s, $C^{1,13}$), 126.83 (s, $C^{6,14}$), 126.66 (s, $C^{3,11}$), 39.77 (s, C^{43}), 39.70 (s, C^{35}), 33.95 (s, C^{45}), 33.91 (s, C^{37}), 29.54 (s, C^{46}), 29.44 (s, C^{38}), 26.74 (s, C^{44}), 26.68 (s, C^{36}), 24.93 (s, C^{47}), 24.90 (s, C^{39}).

MS-ESI (*m/z*): $[M]^-$ Calcd. for $C_{42}H_{40}N_2O_6^-$ 667.3, found 667.3.

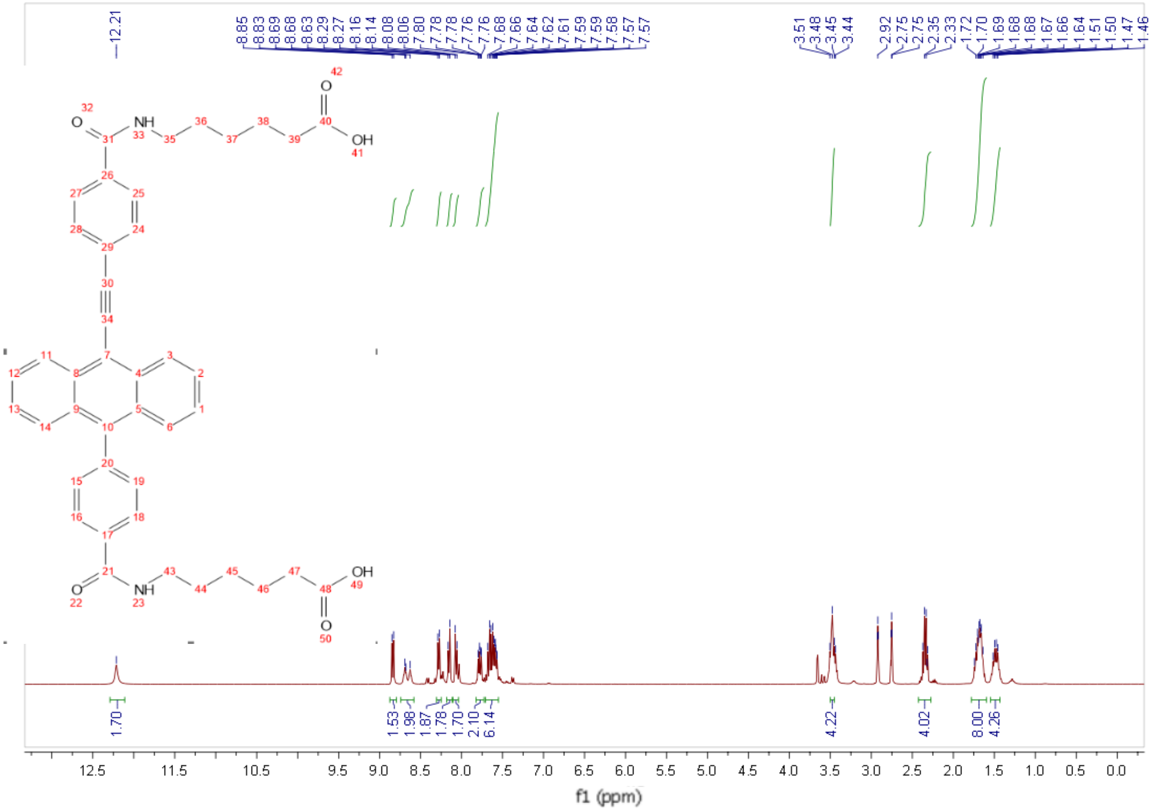

^{1}H NMR spectrum of **15** (**CAEBD-S-COOH**)

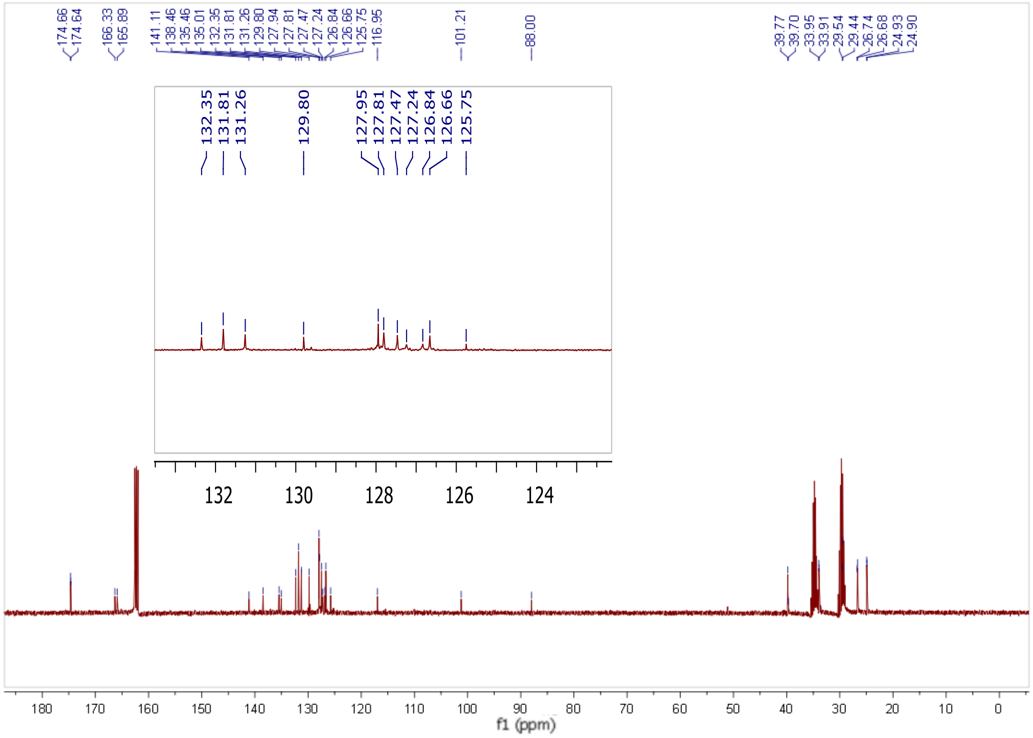

^{13}C NMR spectrum of **15** (**CAEBD-S-COOH**)

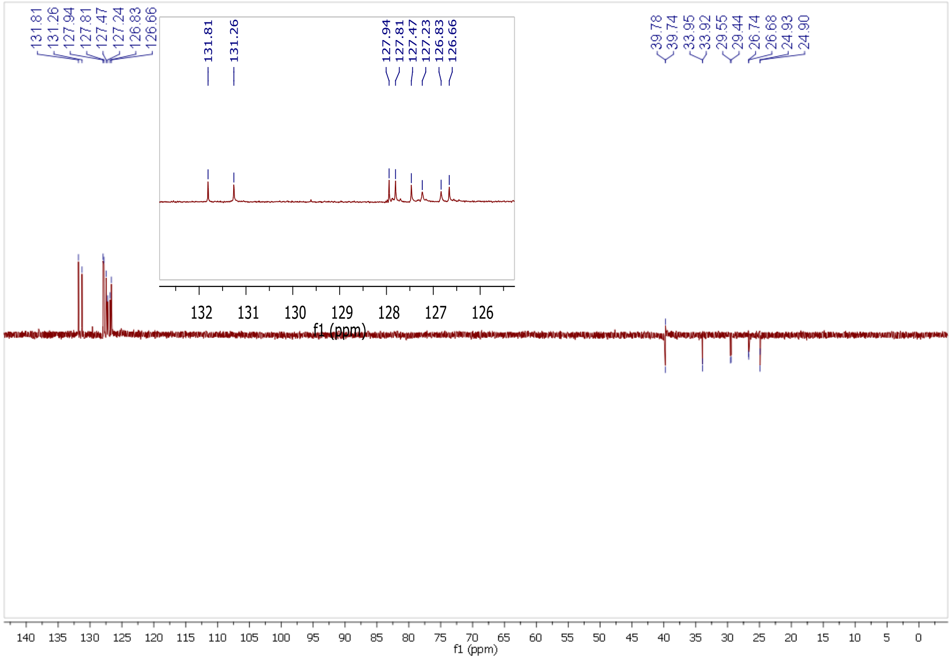

DEPT135 spectrum of **15** (**CAEBD-S-COOH**)

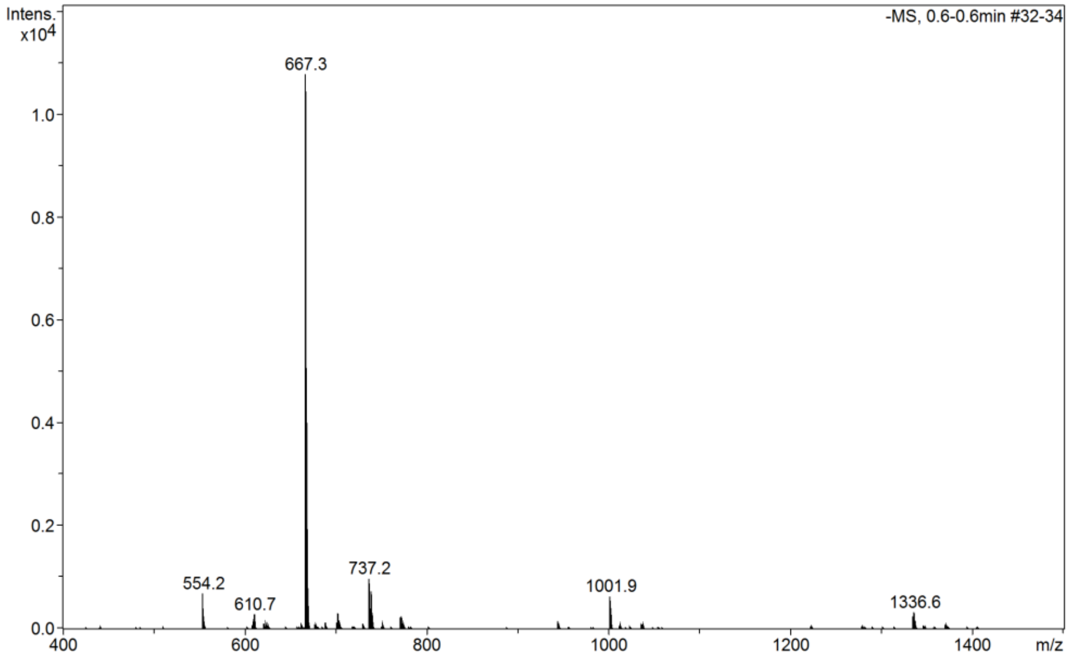

Mass spectrometry of **15** (**CAEBD-S-COOH**)

Section 4. Synthesis and characterization data of Os-S-COOH and intermediates to obtain it

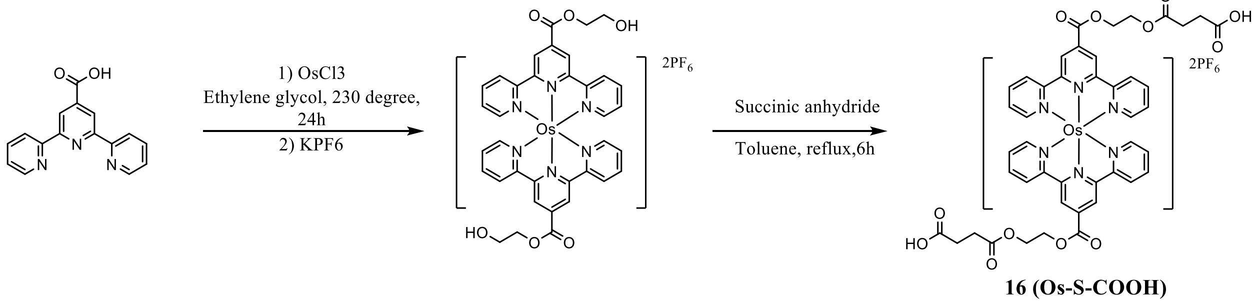

Synthetic route of **16 (Os-S-COOH)**

Osmium(III) chloride hydrate (100.0 mg, 0.34 mmol) and [2,2':6',2''-terpyridine]-4'-carboxylic acid (187.0 mg, 0.68 mmol) were refluxed in 6 mL ethylene glycol for 20 hours at 230 ˚C under continuous stirring. The black solution was obtained. After cooling to room temperature, KPF_6 in distilled water (15 mL) was added, giving black precipitates. The precipitates were filtered and washed with water and MeOH (7:3) and resuspended in toluene. To the black suspension succinic anhydride (680 mg, 6.8 mmol) was added. After refluxing for 8 hours, the solution was concentrated and the resulting solid was washed by ethyl acetate to remove the excess of succinic anhydride, affording the molecule **16** after solvent evaporation (60.0 mg, 12.6%).

^{1}H NMR (400 MHz, $(CD_3)_2SO$) δ: 12.22 (s, 2H, $H^{58,62}$), 9.42 (s, 4H, $H^{7,11,31,35}$), 9.07 (d, J = 7.7 Hz, 4H, $H^{2,16,26,40}$), 7.91 (t, J = 7.8 Hz, 4H, $H^{5,13,29,37}$), 7.43 (d, J = 5.0 Hz, 4H, $H^{6,18,24,36}$), 7.21 (d, J = 6.6 Hz, 4H, $H^{1,17,25,41}$), 4.78 (s, 4H, $H^{44,47}$), 4.59 (s, 4H, $H^{45,48}$), 2.64 (d, J = 6.4 Hz, 4H, $H^{51,54}$), 2.57 (d, J = 5.8 Hz, 4H, $H^{56,60}$).

^{13}C NMR (101 MHz, $(CD_3)_2SO$) δ: 173.91 (s, $C^{57,61}$), 172.82 (s, $C^{50,53}$), 163.74 (s, $C^{19,22}$), 159.03 (s, $C^{4,14,28,38}$), 154.54 (s, $C^{2,16,36,40}$), 153.07 (s, $C^{8.10,32,34}$), 138.88 (s, $C^{6,18,24,36}$), 135.04 (s, $C^{12,30}$), 128.58 (s, $C^{1,17,25,41}$), 125.82 (s, $C^{7,11,31,35}$), 123.01 (s, $C^{5,13,29,37}$), 64.68 (s, $C^{44,47}$), 62.59 (s, $C^{45,48}$), 29.28 (s, $C^{51,54}$), 29.24 (s, $C^{56,60}$).

DEPT 135 (101 MHz, $(CD_3)_2SO$) δ: 153.07 (s, $C^{8,10,32,34}$), 138.87 (s, $C^{6,18,24,36}$), 135.04 (s, $C^{12,30}$), 128.57 (s, $C^{1,17,25,41}$), 125.81 (s, $C^{7,11,31,35}$), 122.99 (s, $C^{5,13,29,37}$), 64.77 (s, $C^{44,47}$), 62.30 (s, $C^{45,48}$), 40.54 (s, $C^{51,54}$), 29.20 (s, $C^{56,60}$).

MS-ESI (*m/z*): $[M]^{+}$ Calcd. for $(C_{44}H_{38}N_6O_{12}Os)/2^{+}$ 517.1, found 517.1.

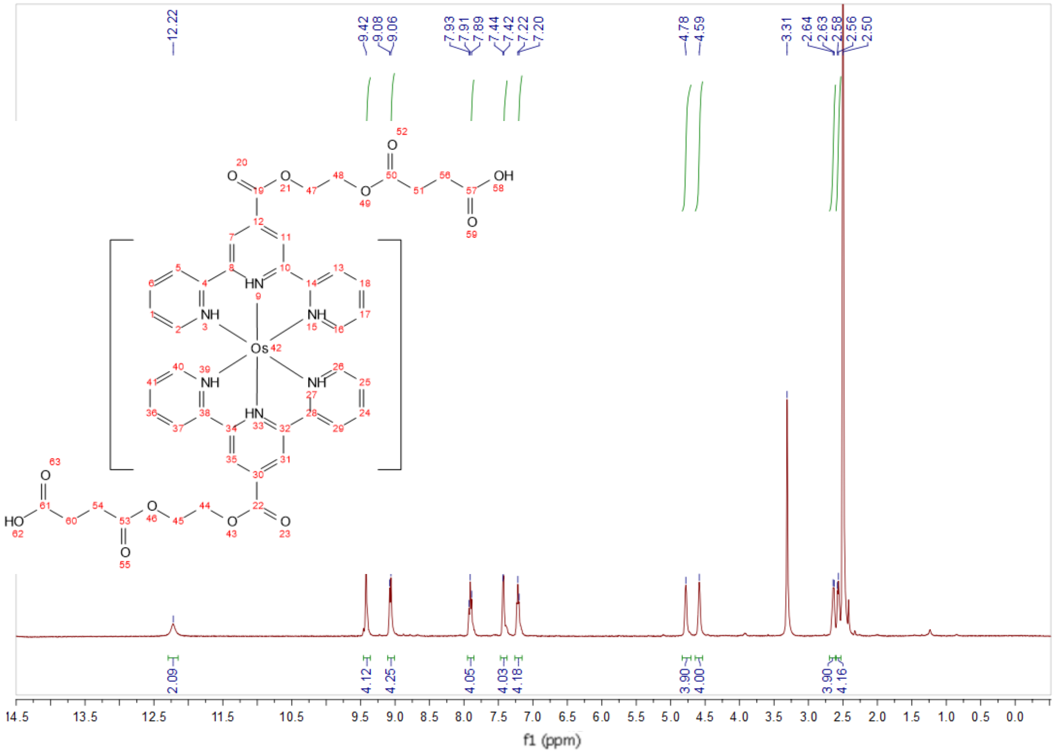

^{1}H NMR spectrum of **16** (**Os-S-COOH**)

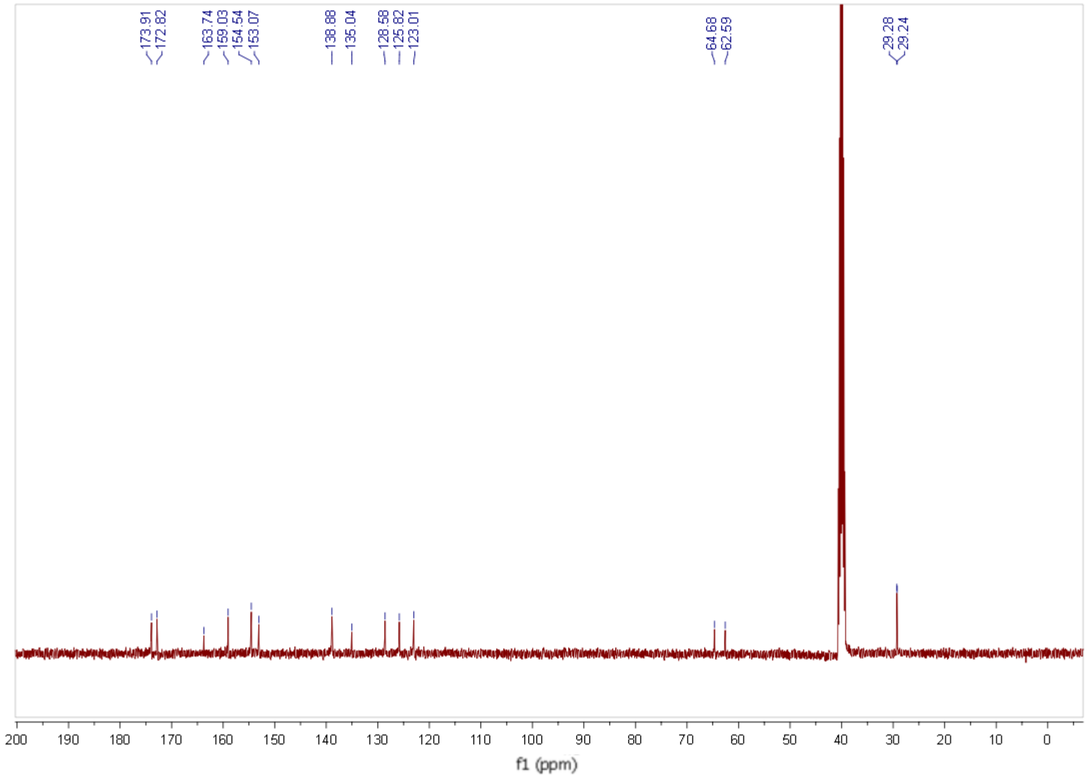

^{13}C NMR spectrum of **16** (**Os-S-COOH**)

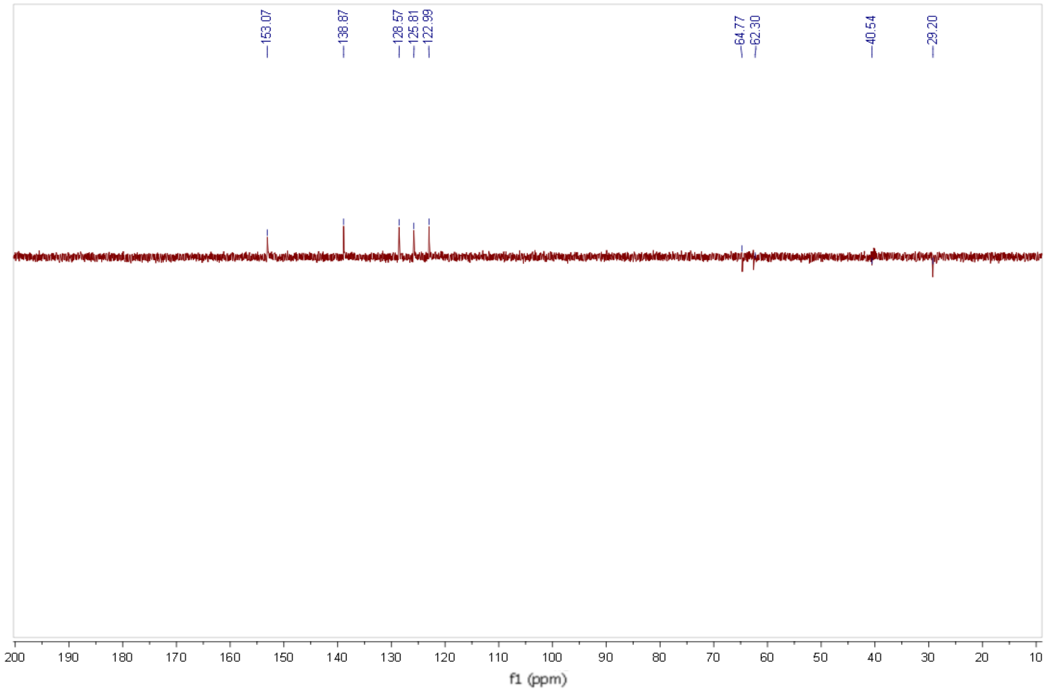

DEPT135 spectrum of **16** (**Os-S-COOH**)

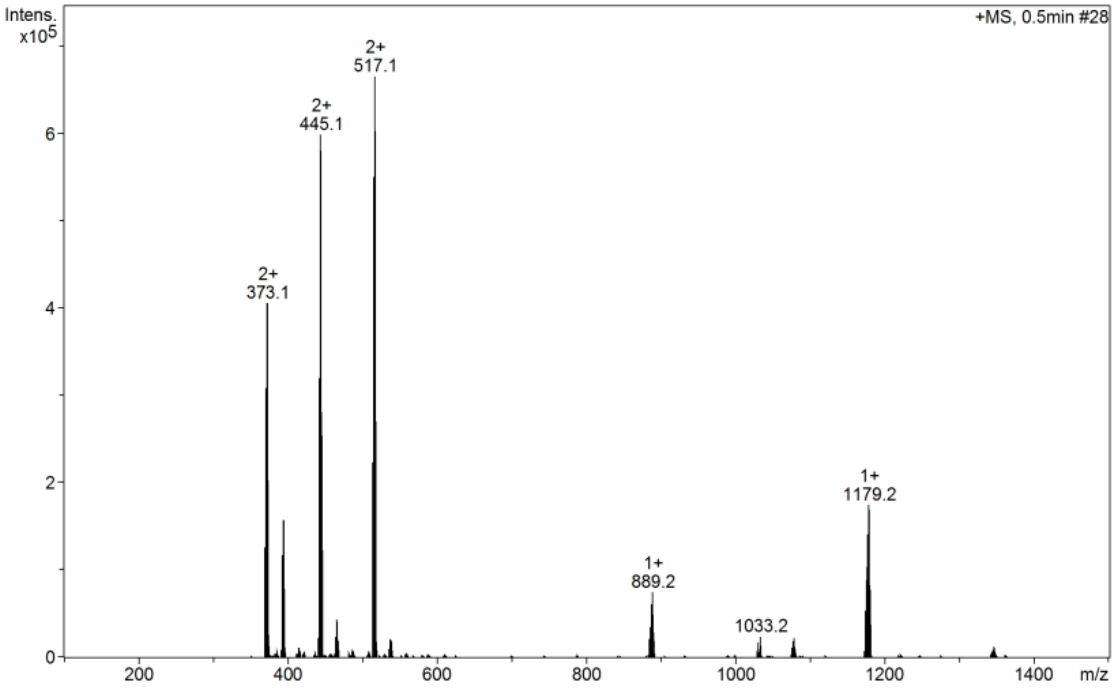

Mass spectrometry of **16** (**Os-S-COOH**)

Section 6: Supplementary Schemes, Tables, Figures and videos

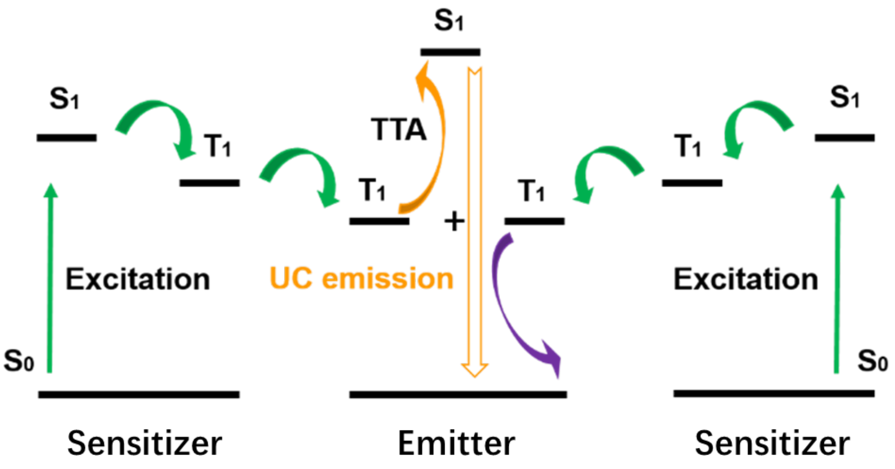

Scheme 1: Schematic illustration of the photoactivatable TTA upconversion

Table S1: Amount of **Pd-S-COOH** and **DPA-S-COOH** used for the reactions and quantification of the amount of each component within the particles in **1-5** batches, using HPLC for **DPA-S-COOH** and ICP-AES for **Pd-S-COOH** and Zr^{2+} amount. Dyes (**DPA-S-COOH** & **Pd-S-COOH**) concentrations and ratios in the different batches: **1**: 1 mg/mL & 0.3125 µg/mL, **2**: 1 mg/mL & 0.625 µg/mL, **3**: 1 mg/mL & 1.25 µg/mL, **4**: 1 mg/mL & 2.5 µg/mL, **5**: 1 mg/mL & 5 µg /mL. $ZrOCl_2$ used in the reaction was always 6 mg.

Entry	DPA-S-COOH/Pd-S-COOH before reaction (molar ratio)	DPA-S-COOH/Pd-S-COOH within CPNs (molar ratio)	Molar ratio (Zr/Pd)	Weight ratio (Zr/Pd)	UC/Ph (Integral emission intensity)
1	5127	9974	22247	19070	5.00
2	2563	6151	11776	10094	5.53
3	1281	2902	5703	4889	6.54
4	640	1262	2479	2125	4.27
5	320	655	1327	1138	2.64

Table S2: Amount of **Os-S-COOH** and **CAEBD-S-COOH** used for the reactions and quantification of the amount of each component within the particles in **1-3** batches, using HPLC for **CAEBD-S-COOH** and ICP-AES for **Os-S-COOH** and Zr^{2+}. Dyes (**CAEBD-S-COOH** & **Os-S-COOH**) concentrations and ratios in the different batches: **1**, 1.5 mg/mL & 12.5 µg/mL, **2**, 1.5 mg/mL & 25 µg/mL, **3**, 1.5 mg/mL & 50 µg/mL. $ZrOCl_2$ used in the reaction was always 6 mg.

Entry	DAEBD-S-COOH/Os-S-COOH before reaction (molar ratio)	DAEBD-S-COOH/Os-S-COOH within CPNs (molar ratio)	Molar ratio (Zr/Os)	Weight ratio (Zr/Os)	UC/Ph (Integral emission intensity)
1	240	208	565	271	2.24
2	120	89	235	113	7.32
3	60	51	141	68	1.98

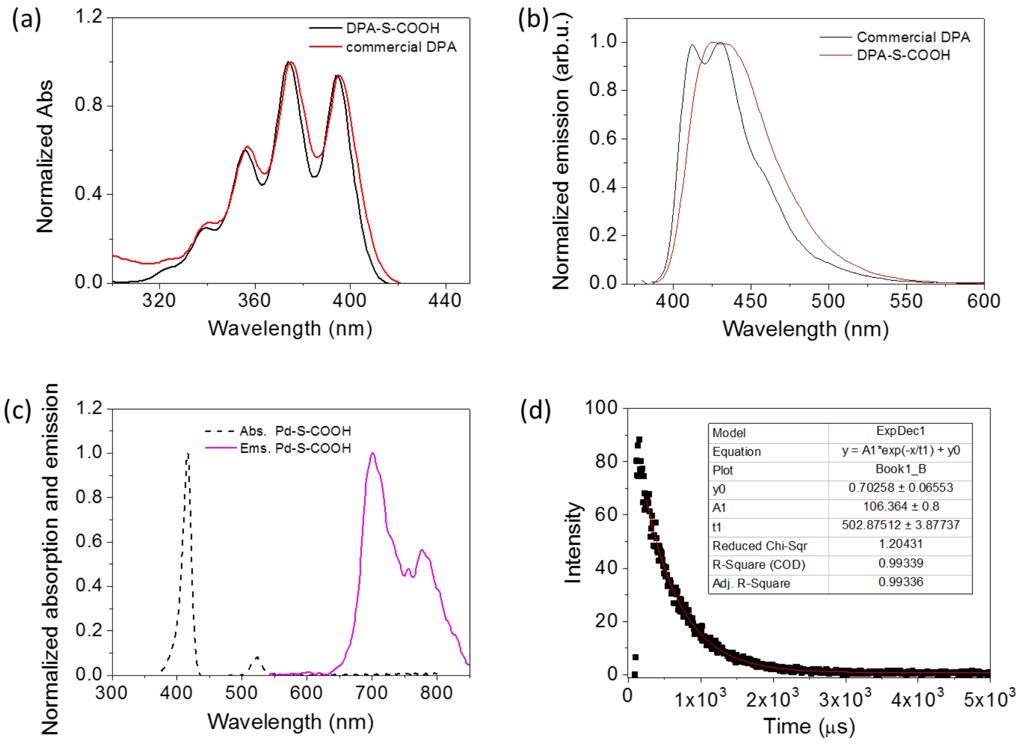

Figure S1: (a) Absorption comparison between commercial **DPA** (4.0 * 10^{-5} M) & **DPA-S-COOH** (4.0 * 10^{-5} M) in DMF; (b) Emission comparison between commercial **DPA** (4.0 * 10^{-5} M) & **DPA-S-COOH** (4.0 * 10^{-5} M) (λ_{exc} = 375 nm) in DMF; (c) absorption (3.3 * 10^{5} M^{-1} cm^{-1})/emission and (d) phosphorescence lifetime of **Pd-S-COOH** (1.0 * 10^{-6} M) in DMF (λ_{exc} = 532 nm). Fitting was done with monoexponential decay function.

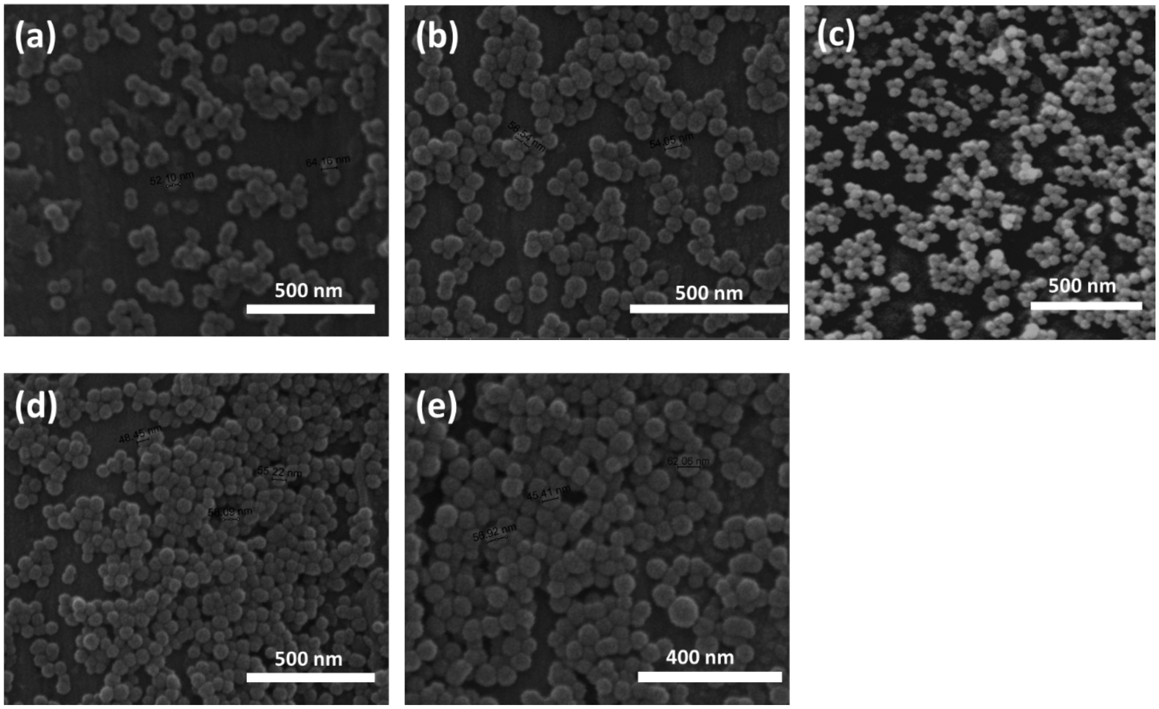

Figure S2: SEM images of (a)**1**, (b)**2**, (c)**3**, (d)**4**, (e)**5** (CPNs made by these ratio **1**: 1 mg/mL & 0.3125 μg/mL, **2**: 1 mg/mL & 0.625 μg/mL, **3**: 1 mg/mL & 1.25 μg/mL, **4**: 1 mg/mL & 2.5 μg/mL, **5**: 1 mg/mL & 5 μg /mL).

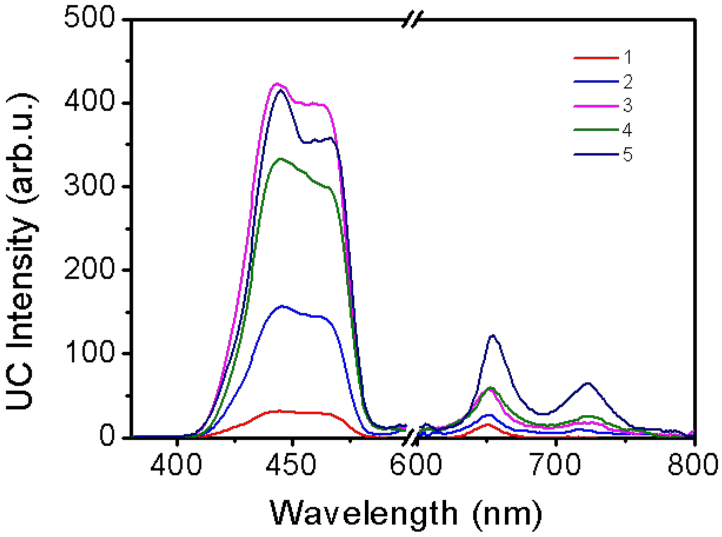

Figure S3: Upconversion emission of five different **VVUC1-CPNs** water suspensions (λ_{exc} = 532 nm) obtained by using different **DPA-S-COOH/Pd-S-COOH** ratio (**1**: 1 mg/mL & 0.3125 μg/mL, **2**: 1 mg/mL & 0.625 μg/mL, **3**: 1 mg/mL & 1.25 μg/mL, **4**: 1 mg/mL & 2.5 μg/mL, **5**: 1 mg/mL & 5

µg /mL). Magenta subtractive dichroic color filter, filter was used to cut the 532 nm excitation beam.

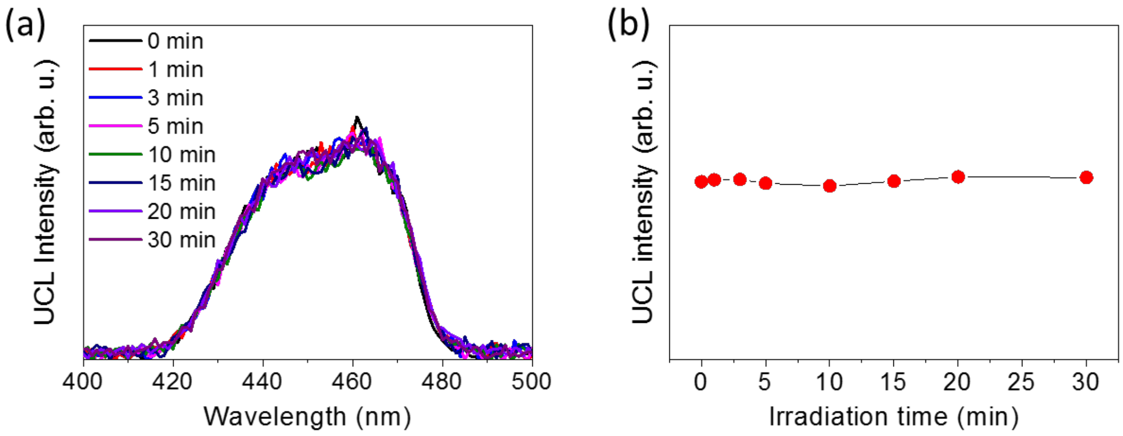

Figure S4: (a) UC emission spectra and (b) integrated UC intensities of the **VVUC1-CPNs** (1.0 mg/mL) upon prolonged irradiation (up to 30 min) with CW 532 nm laser (1.2 W/cm^2).

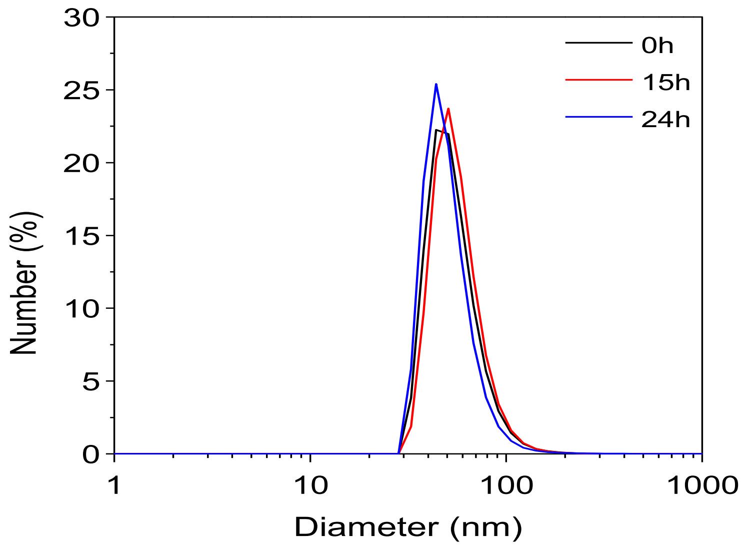

Figure S5: DLS of **VVUC1-CPNs** (1.0 mg/mL) suspension in MilliQ® water during 24h, for the colloidal stability study.

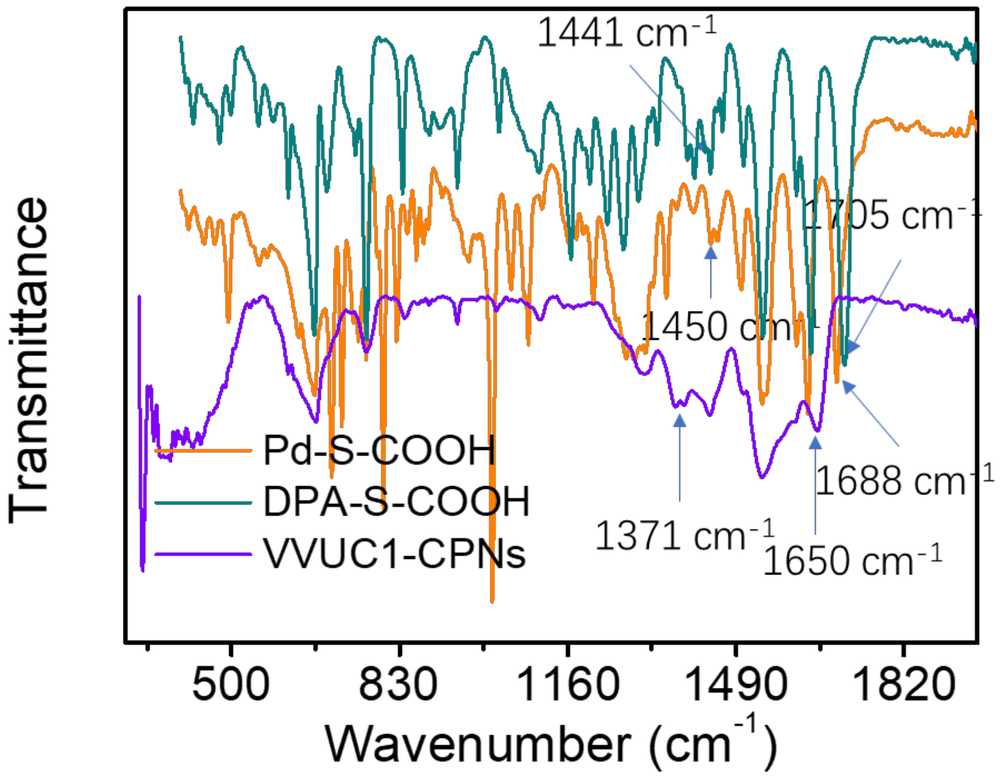

Figure S6: FTIR of **VVUC1-CPNs**.

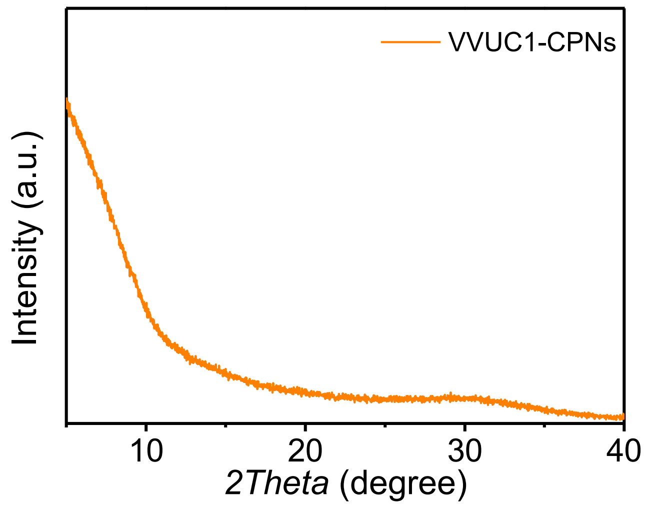

Figure S7: XRD of **VVUC1-CPNs**.

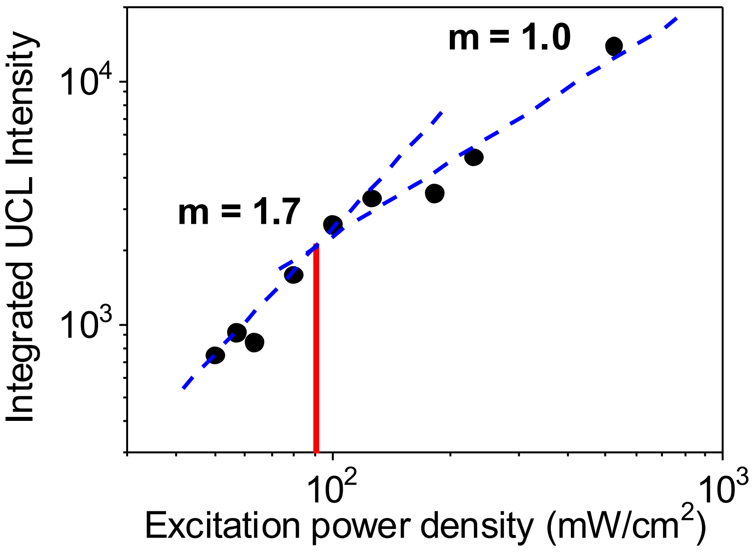

Figure S8: Logarithm plot of integrated UCL intensity against excitation power density. Excitation intensity threshold (I_{th}) was determined from the interception of the two fitting lines in the quadratic and linear regimes.

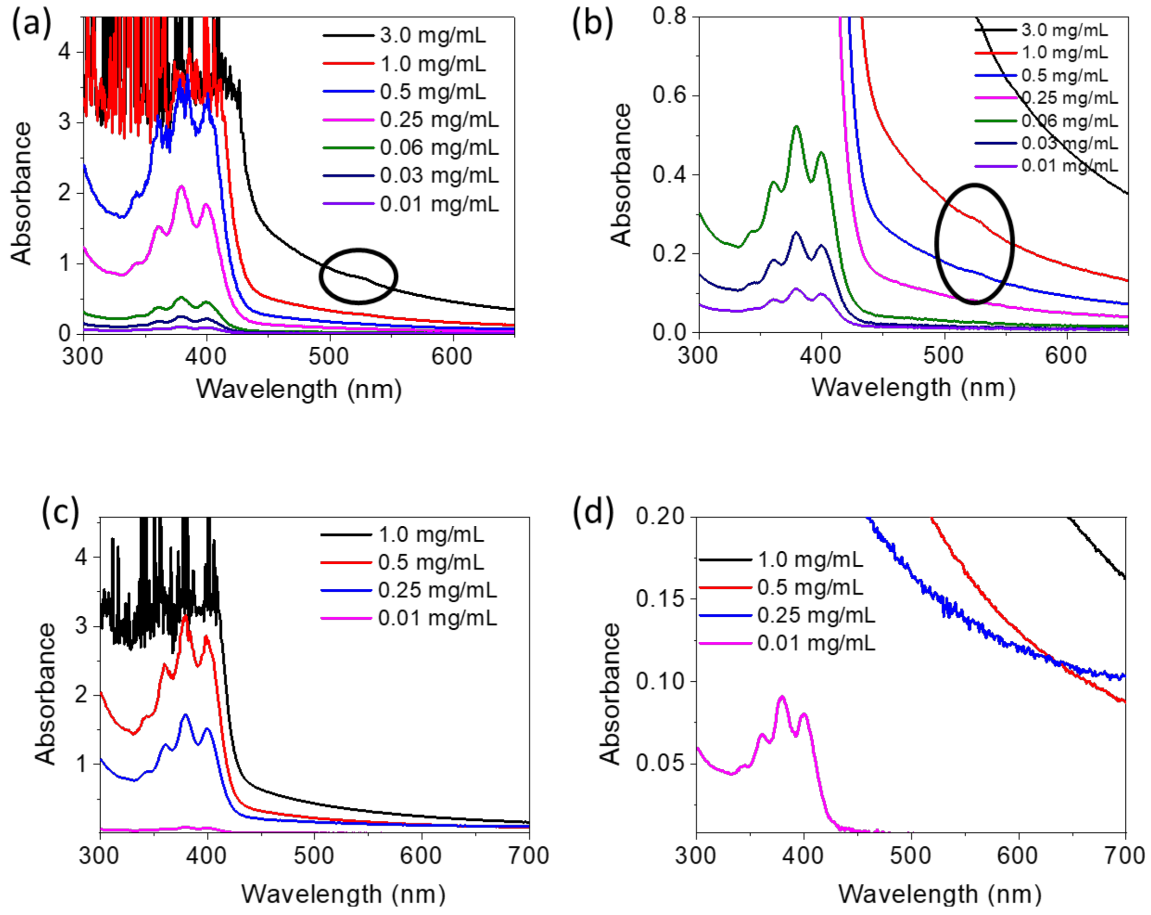

Figure S9: Absorption spectra of (a-b) **VVUC1-CPNs** (0.01, 0.03, 0.06, 0.25, 0.50 1.0, and 3.0 mg/mL) and (c-d) **DPA-S-CPNs** aqueous suspensions at different concentrations (0.01, 0.25, 0.5, 1.0 mg/mL). Plots (b) and (d) are zoom of (a) and (c), respectively, made to show the scattering, the absorption band of the **Pd-S-COOH** and of the **DPA-S-COOH** of the lower concentrated CPNs.

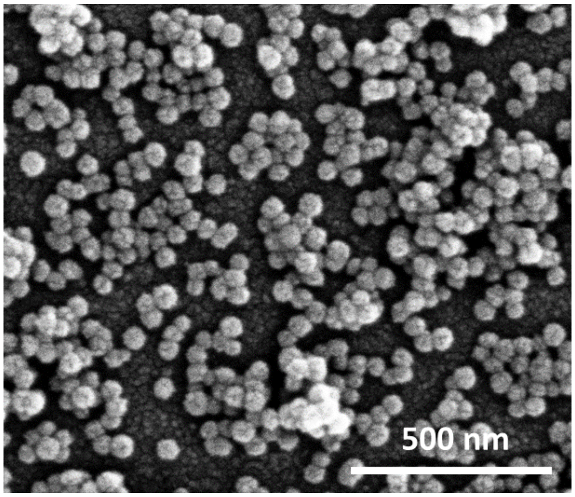

Figure S10: SEM image of **DPA-S-CPNs**.

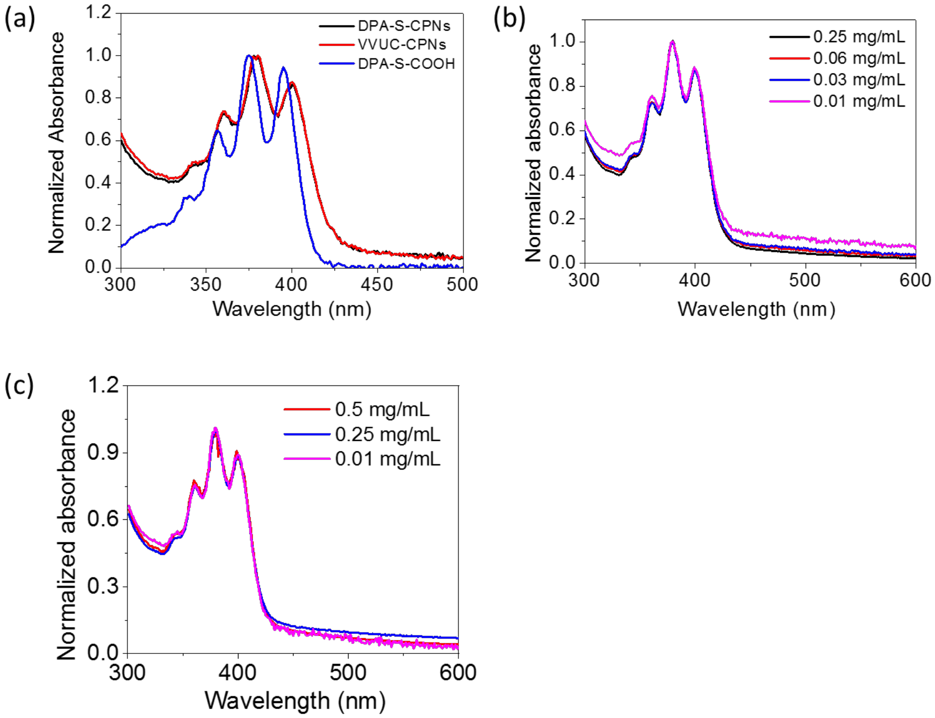

Figure S11: (a) Normalized absorption spectra of **DPA-S-COOH** in DMF solution (1 * 10^{-5} M), **VVUC1-CPNs** (0.01 mg/mL) and **DPA-S-CPNs** (0.01 mg/mL); normalized absorption spectra of (b) **VVUC1-CPNs** (0.01, 0.03, 0.06, and 0.25 mg/mL) and (c) **DPA-S-CPNs** (0.01, 0.25, and 0.5 mg/mL) suspensions at different concentrations.

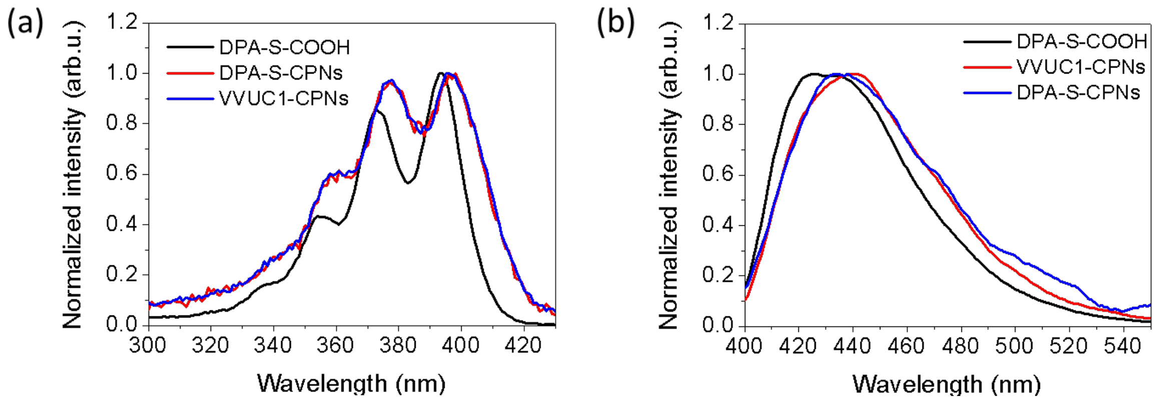

Figure S12: (a) Excitation (λ_{emi} = 450 nm) and (b) emission (λ_{exc} = 375 nm) spectra of a DMF solution of **DPA-S-COOH** (1.0 * 10^{-5} M) and aqueous suspensions of **VVUC1-CPNs** (1.0 mg/mL) and **DPA-S-CPNs** (1.0 mg/mL).

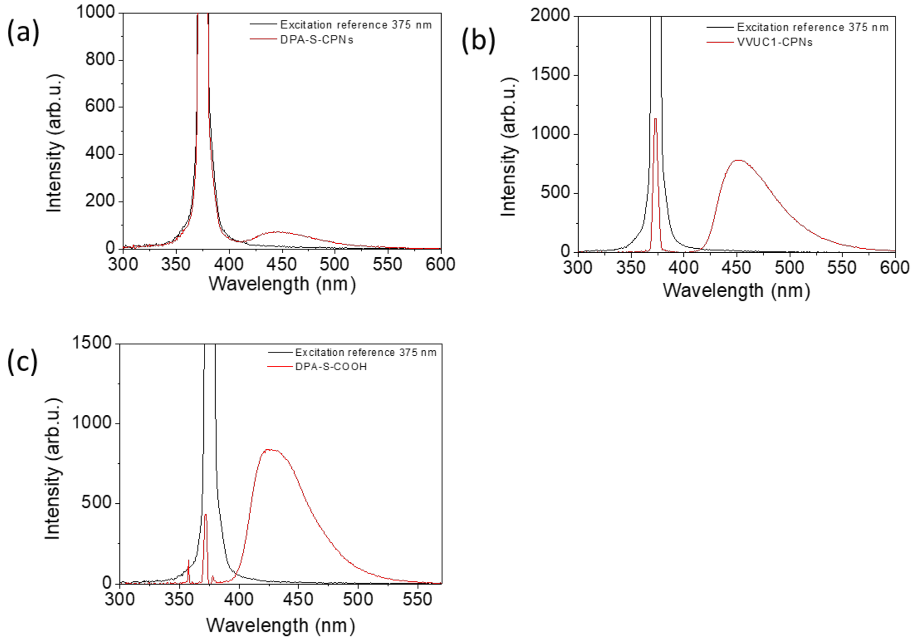

Figure S13: Fluorescence spectra recorded to determine the absolute emission quantum yield of **DPA-S-COOH** in (a) **DPA-S-CPNs** (0.01 mg/, 18.5%), (b) **VVUC1-CPNs** (0.01 mg/mL, 34.2%), and (c) DMF (1*10^{-5} M, 84.2%), exciting at λ_{exc} = 375 nm.

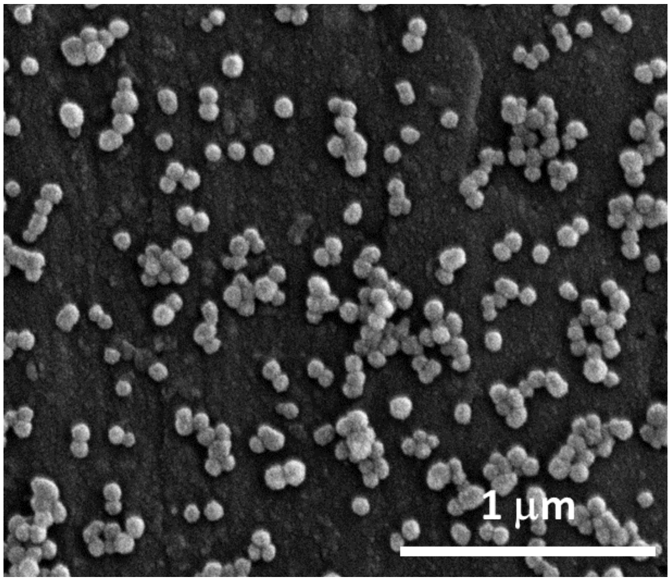

Figure S14: SEM image of **Pd-S-CPNs**.

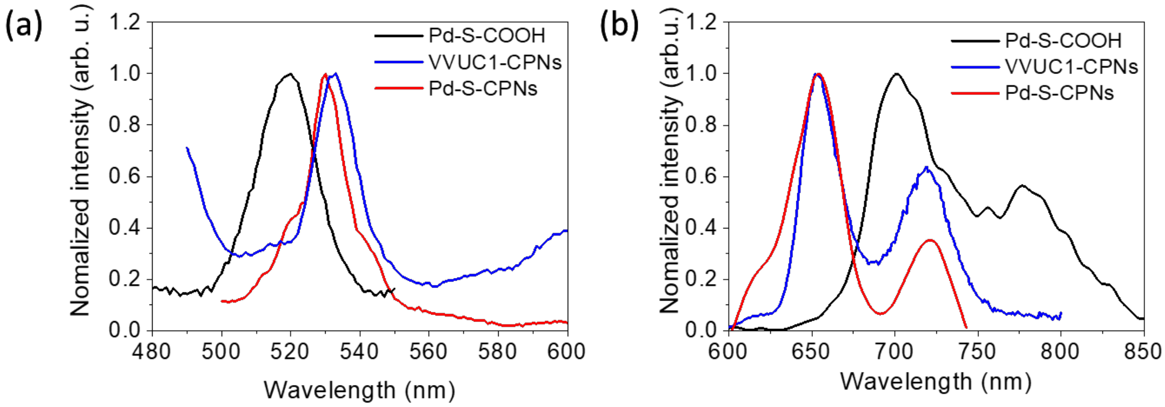

Figure S15: (a) Excitation (λ_{emi} = 650 nm) and (b) emission (λ_{exc} = 532 nm) spectra of a DMF solution of **Pd-S-COOH** (1.0 * 10^{-5} M) and aqueous suspensions of **VVUC1-CPNs** (1.0 mg/mL) and **Pd-S-CPNs** (1.0 mg/mL).

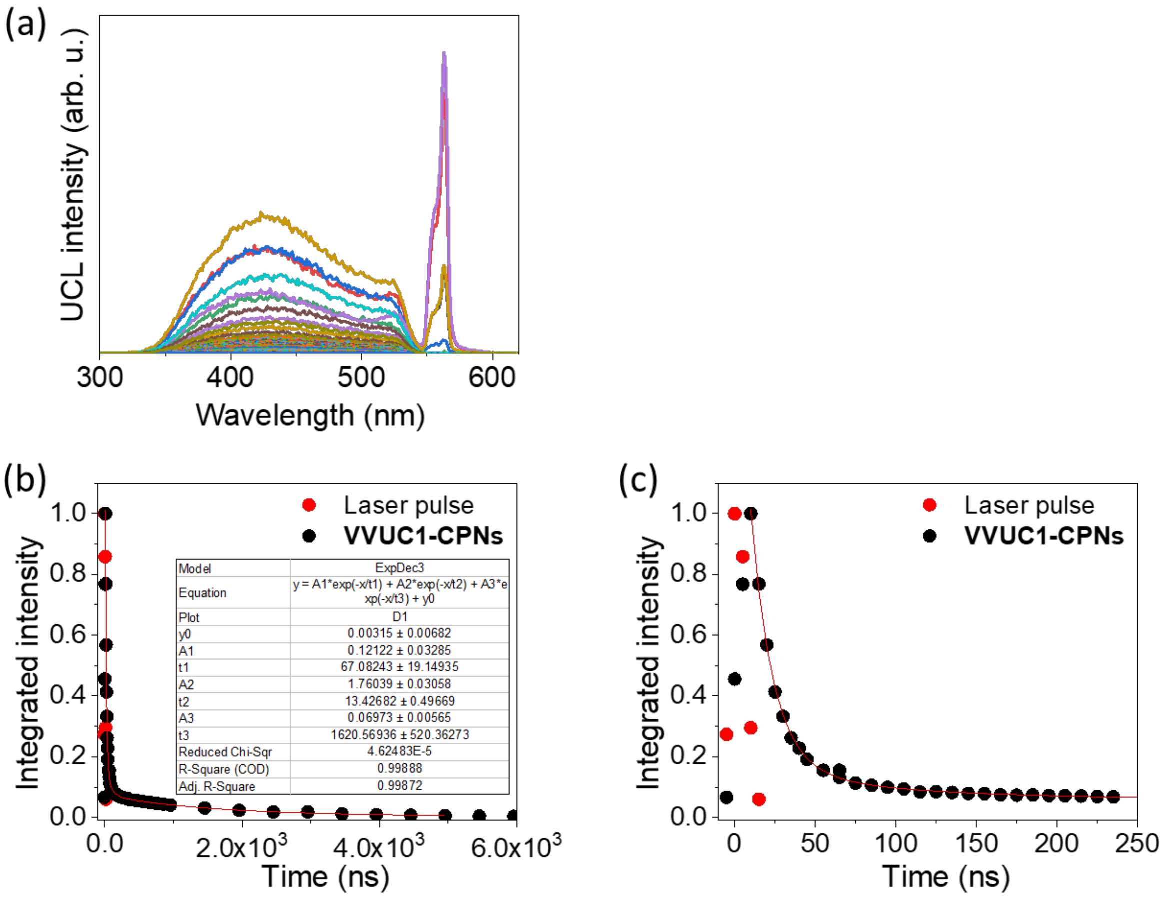

Figure S16: (a) UC emission spectra of **VVUC1-CPNs** obtained upon irradiation with a nanosecond pulsed laser (532 nm, 2 Hz, 10 mW), recorded at different delays after the laser pulse. (b) Decay profile of the UC emission of **VVUC1-CPNs** upon excitation at λ_{exc} = 532 nm with nanoseconds pulsed laser. Each point was obtained from the integrated spectra recorded at different time delays respect to the pulse (t = 0). The rise and decay of the scattering peak of the laser is also reported and was used as reference for the t = 0 ns. (c) Zoom of the decay of the UC emission during the first 250 ns.

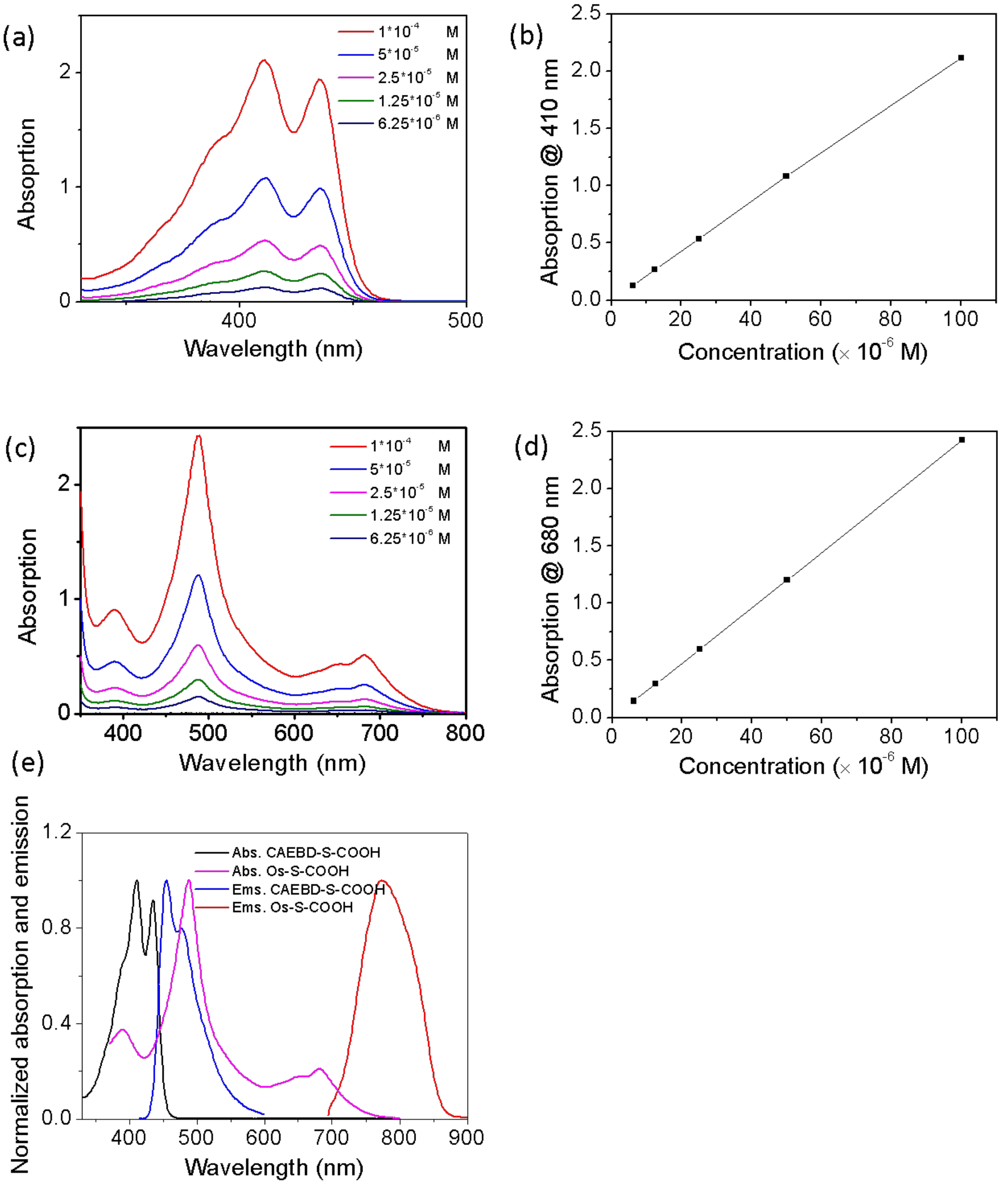

Figure S17: (a,c) UV-Vis absorption spectra of **CAEBD-S-COOH** ($6.25 * 10^{-6}$, $1.25 * 10^{-5}$, $2.5 * 10^{-5}$, $5 * 10^{-5}$, and $1.0 * 10^{-4}$ M) and **Os-S-COOH** ($6.25 * 10^{-6}$, $1.25 * 10^{-5}$, $2.5 * 10^{-5}$, $5.0 * 10^{-5}$, and $1.0 * 10^{-4}$ M) at various concentrations in DMF and (b,d) linear fitting of the absorbance at 410 nm (ε^{410nm} = 2.12 X 10^4 M^{-1} cm^{-1}) and 680 nm (ε^{680nm} = 2.40 X 10^4 M^{-1} cm^{-1}), respectively. (e) Absorption and emission (λ_{exc} = 410 nm, λ_{exc} = 680 nm, respectively) spectra of **CAEBD-S-COOH** ($5.0 * 10^{-4}$ M) and **Os-S-COOH** ($1.0 * 10^{-3}$ M) in DMF.

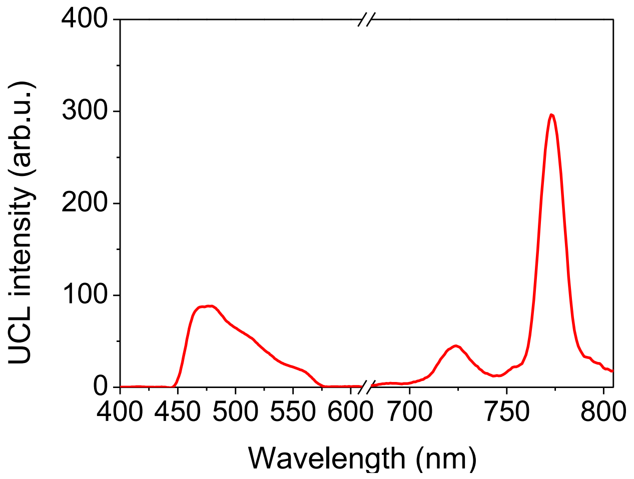

Figure S18: UC emission of a degassed DMF solution of the **Os-S-COOH** ($5.0 * 10^{-4}$ M) and **CAEBD-S-COOH** (10^{-3} M), irradiated with 650 nm CW laser. Subtractive cyan dichroic filter was used.

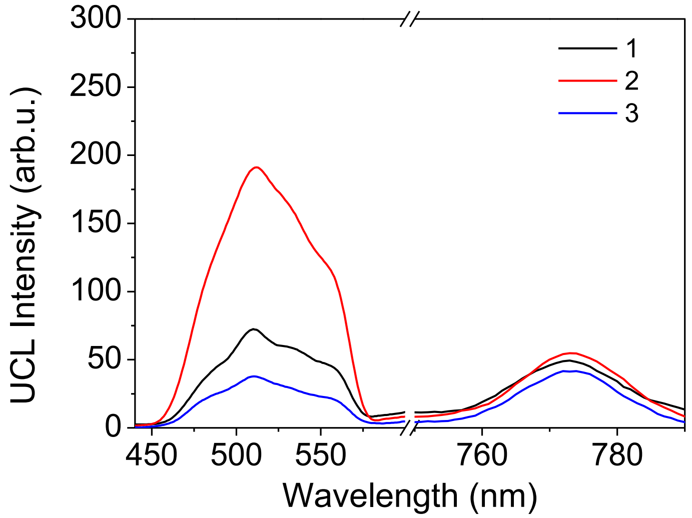

Figure S19: UC emission of five different **VVUC2-CPNs** obtained by using different **CAEBD-S-COOH** and **Os-S-COOH** ratio (**1**, 1.5 mg/mL & 12.5 µg/mL, **2**, 1.5 mg/mL & 25 µg/mL, **3**, 1.5 mg/mL & 50 µg/mL) upon 650 nm laser irradiation. Subtractive cyan dichroic filter was used.

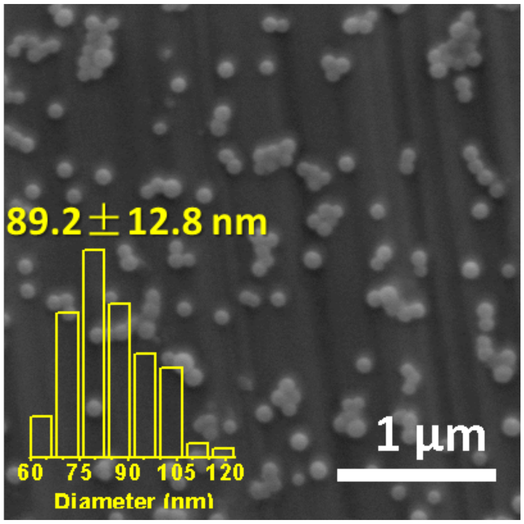

Figure S20: SEM image of **VVUC2-CPNs** (Table S2, entry 2).

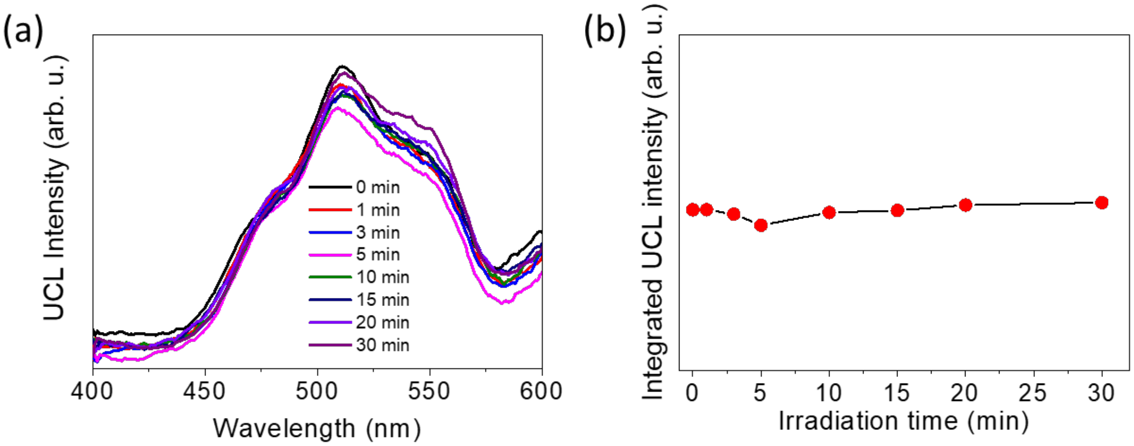

Figure S21: (a) UC emission spectra and (b) integrated UC emission intensity of the **VVUC2-CPNs** (1.0 mg/mL) upon prolonged irradiation (up to 30 min) with CW 650 nm laser (6.6 W/cm^2).

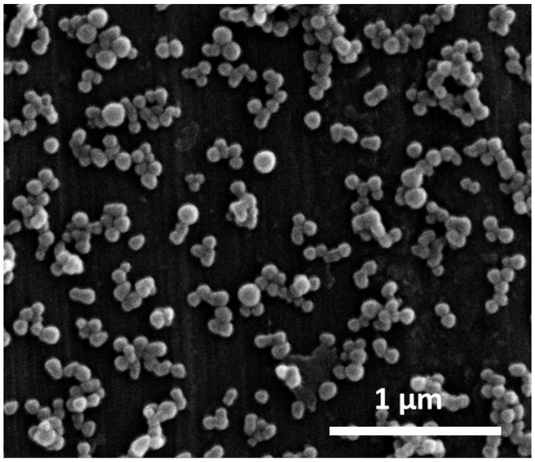

Figure S22: SEM image of the **VVUC2-CPNs** after 30 min irradiation with CW 650 nm laser.

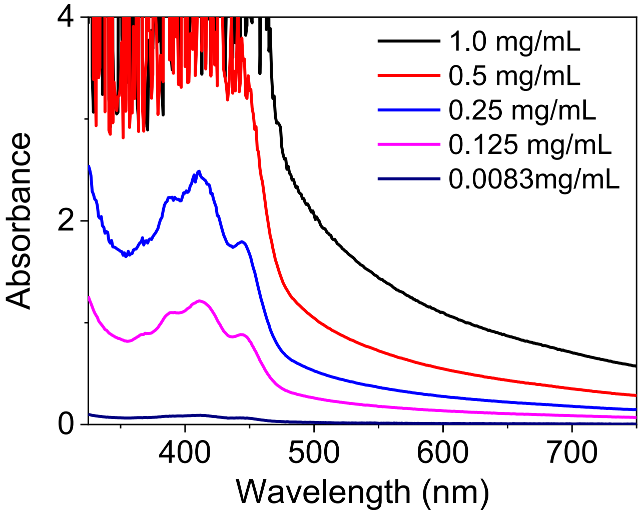

Figure S23: Absorption spectra of aquesous suspensions of **VVUC2-CPNs** of different concentrations (0.0083, 0.125, 0.25, 0.5, 1.0 mg/mL).

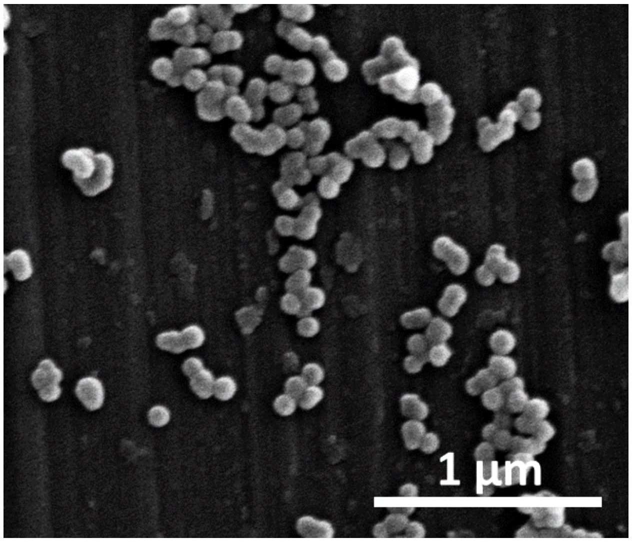

Figure S24: SEM image of the **CAEBD-S-CPNs**.

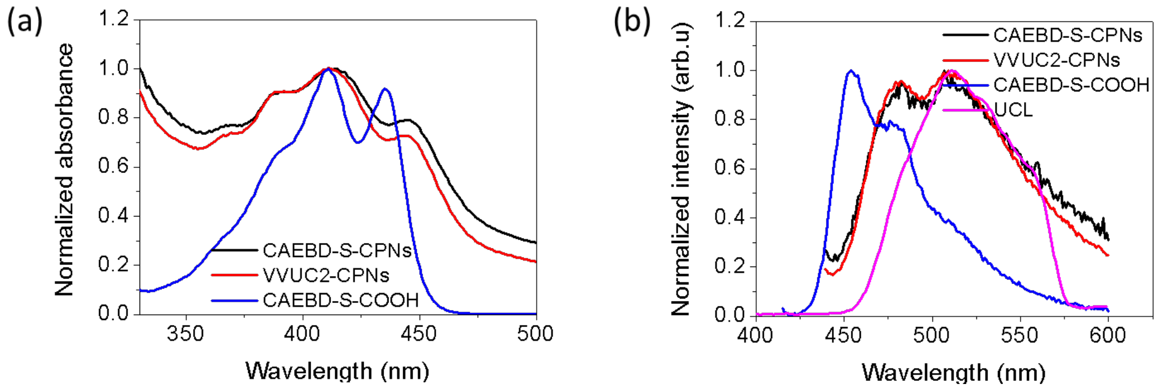

Figure S25: Normalized (a) absorption and (b) emission spectra (λ_{exc} = 410 nm) of **CAEBD-S-COOH** solution (1.0 * 10^{-5} M), and aqueous suspensions of **VVUC2-CPNs** (1.0 mg/mL) and **CAEBD-S-CPNs** (1.0 mg/mL).

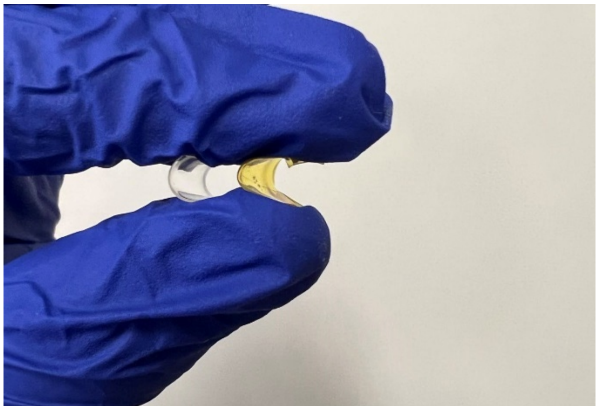

Figure S26: **VVUC1-CPNs@PVA** and **VVUC2-CPNs@PVA** films bended with fingers, to show flexibility.

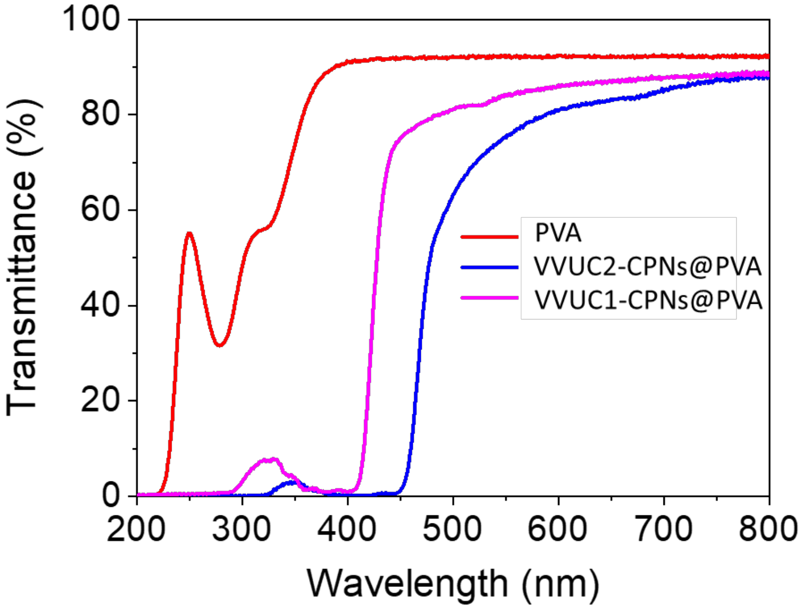

Figure S27: Transmittance of plain PVA, **VVUC2-CPNs@PVA** and **VVUC1-CPNs@PVA** films.

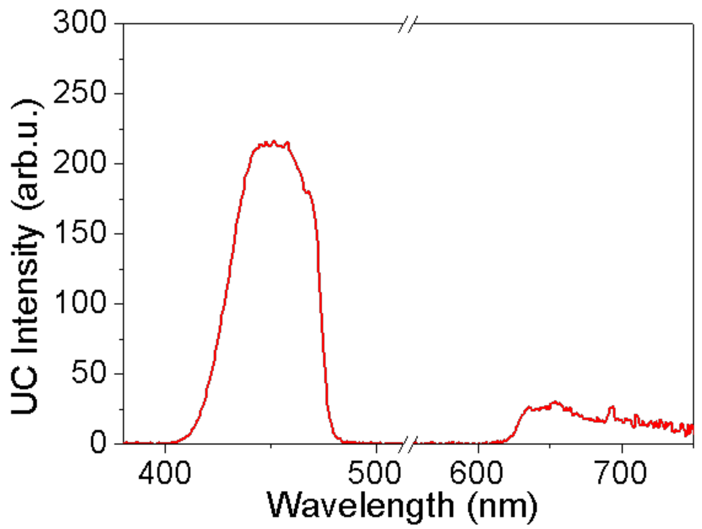

Figure S28: UC emission of **VVUC1-CPNs@PVA** film (λ_{exc} = 532 nm). Magenta subtractive dichroic color filter, filter was used to cut the 532 nm excitation beam.

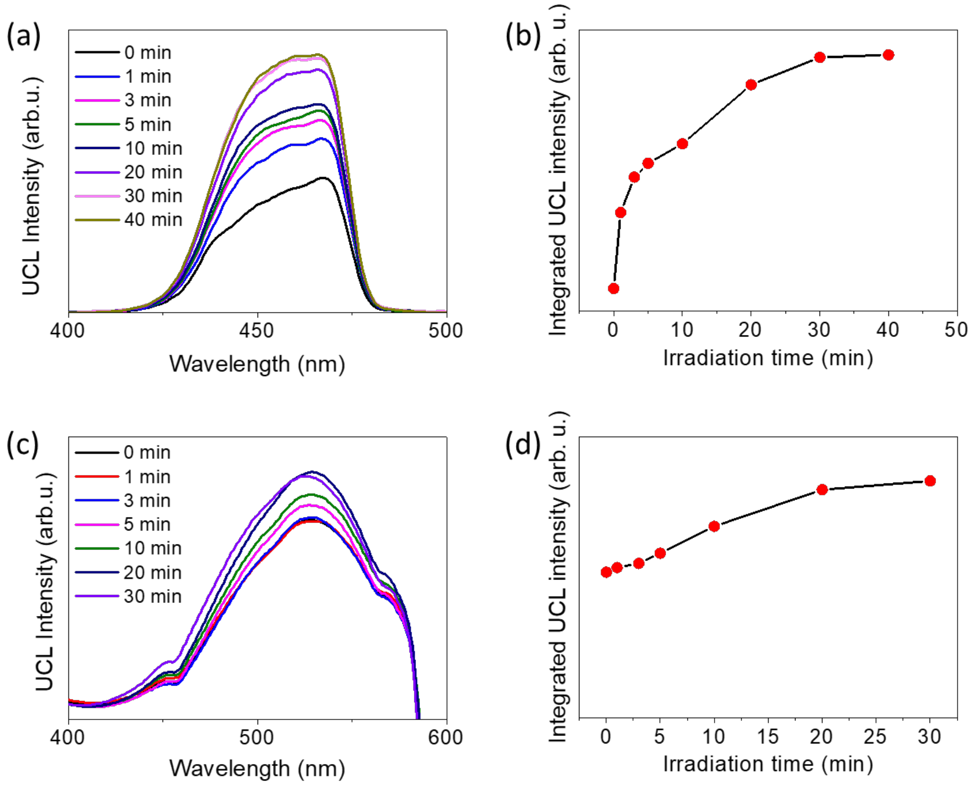

Figure S29: (a, c) UC emission spectra and (b, d) integrated UC intensities of the PVA films embedding (a, b) **VVUC1-CPNs** (1.0 mg/mL) and (c, d) **VVUC2-CPNs**, upon prolonged irradiation (30-40 min) with CW 532 (1.2 W/cm^2) and 650 nm lasers (6.6 W/cm^2), respectively.

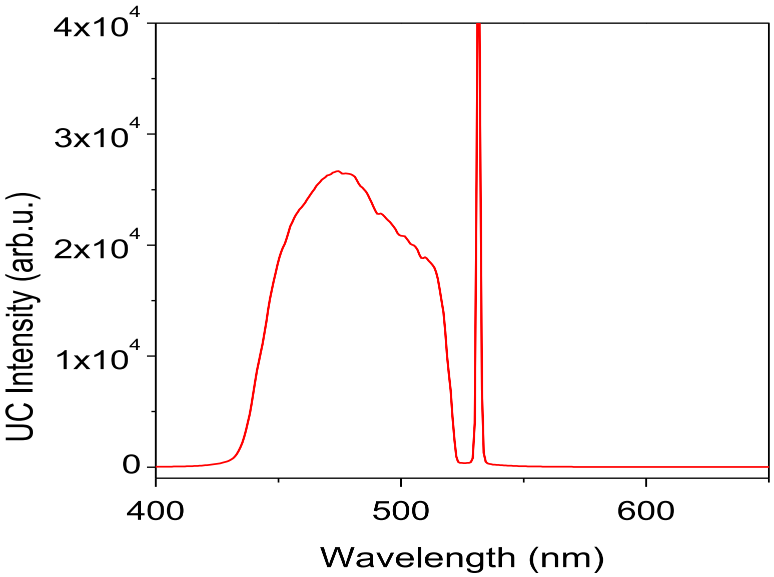

Figure S30: UC emission recorded at the edge of **VVUC1-CPNs@PVA** film upon laser irradiation (λ_{exc} = 532 nm) at the center of the film. Short-pass filter ($\lambda_{cut\text{-}off}$ = 532 nm) was used.

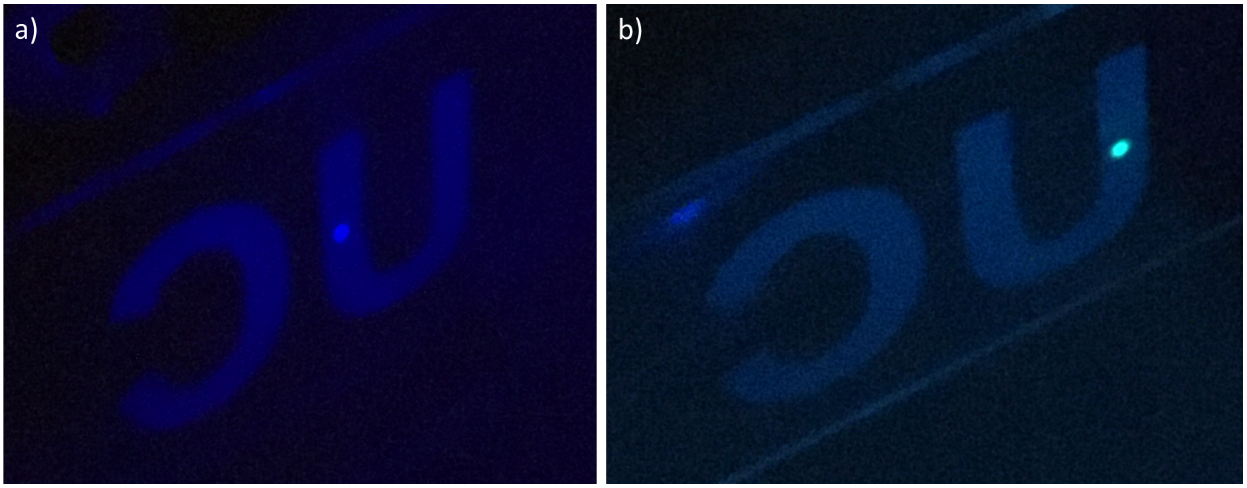

Figure S31: Photo of the UC emission of **VVUC1-CPNs** and **VVUC2-CPNs**-based “UC” patterns, irradiated with *a)* 532 nm and *b)* 650 nm CW laser, respectively.

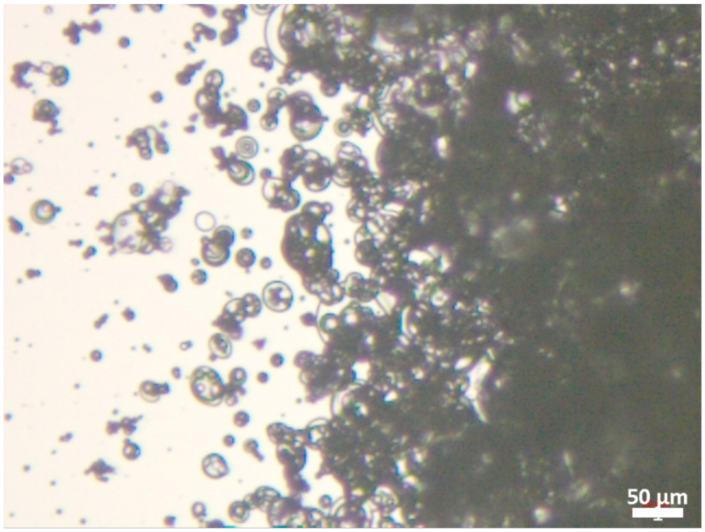

Figure S32: Optical microscope image of PVA microparticles containing the UC-CPNs.

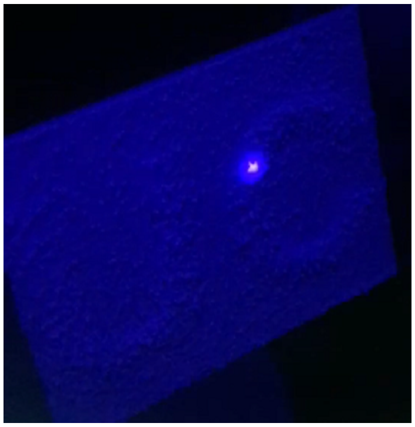

Figure S33: Screenshot of the video S2 showing the scanning with 532 nm CW laser irradiation over spray-coated **VVUC1-CPNs**-based patterns.

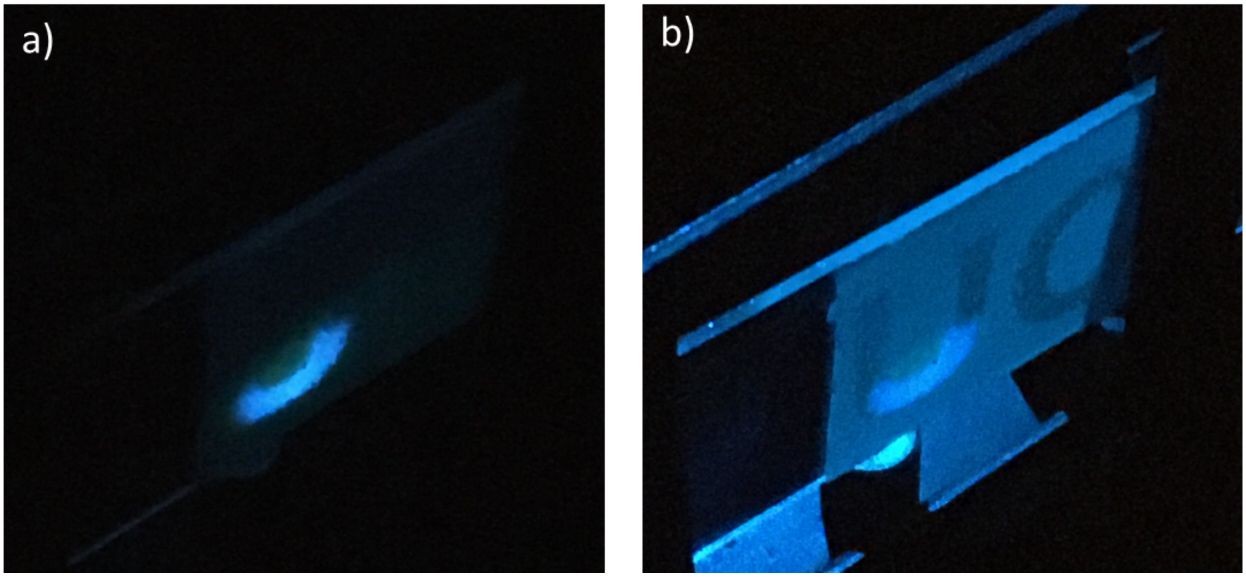

Figure S34: Screenshot of the video S4 showing the UC emission of the printed label upon irradiation with a 532 nm nanoseconds pulsed Nd:YAG laser (10 Hz), with spot size of 1 cm diameter. The large beam spot area allows irradiating simultaneously regions with and without UC printed ink, around the “U” of the “UC” pattern. The emission (UC) is selectively observed in the “U” of the “UC” pattern (bottom part) as it is where the printed UC ink is deposited. The background does not show UC. Images are taken *a)* in the absence and *b)* in the presence of some white flashlight to show the emitting area is part of the “UC” label.

Video S1: Video showing the scanning with a UV beam over the **VVUC1-CPNs**-based spray-coated pattern deposited onto **DPA-S-CPNs** background. The emission does not change all over the scanning as **DPA-S-COOH** emission is always activated.

Video S2: Video showing the scanning with the 532 nm CW laser over the **VVUC1-CPNs**-based spray-coated pattern deposited onto **DPA-S-CPNs** background. The UC emission only appears when the beam crosses the “UC” pattern.

Video S3: Video showing the scanning with the 650 nm CW laser over the **VVUC2-CPNs**-based spray-coated pattern deposited onto **CAEBD-S-CPNs** background. The UC emission only appears when the beam crosses the “UC” pattern.

Video S4: video showing the scanning with the 532 nm Nd:YAG nanoseconds pulsed laser over the **VVUC1-CPNs**-based spray-coated pattern deposited onto **DPA-S-CPNs**. The UC emission only appears from the "UC" pattern.

[1] K. Suzuki, A. Kobayashi, S. Kaneko, K. Takehira, T. Yoshihara, H. Ishida, Y. Shiina, S. Oishic, S. Tobita, *Phys. Chem. Chem. Phys.* **2009**, *11*, 9850-9860.
